# Passive Color Imaging Through Macroscopic Strong Scattering Media

**DOI:** 10.1002/advs.77876

**Published:** 2026-10-29

**Authors:** Renrui Wang, Xiaoyu Wang, Jianyu Wang, Zhiyu Yang, Xiangyang Ji

**Affiliations:** ^1^ Shenzhen International Graduate School Tsinghua University Shenzhen P.R. China; ^2^ Department of Automation Tsinghua University Beijing P.R. China; ^3^ Yau Mathematical Sciences Center Tsinghua University Beijing P.R. China; ^4^ Department of Electrical and Computer Engineering National University of Singapore Singapore Singapore

**Keywords:** computational imaging, passive information reconstruction, real‐world validation, sCMOS array, strong scattering media

## Abstract

De‐scattering imaging seeks to recover target information from the scattered images, which has increasingly garnered attention due to its wide‐ranging applications across various fields. Scattering media such as fog and turbid water can severely degrade imaging quality, thereby obstructing the accurate recovery of important details for identifying targets. Existing methods struggle to maintain de‐scattering performance in practical complex strong scattering media. To address this challenge, we propose an sCMOS array detection system that utilizes color channel simulation and independent exposure adjustment mechanisms. Our system significantly extends the dynamic range and enhances the signal‐to‐noise ratio of raw scattering data. By introducing a passive colorful de‐scattering pipeline, we achieve high‐quality reconstruction in strong scattering conditions that preserves the target's chromatic identity. Experiments in outdoor foggy conditions demonstrate the system's effectiveness in recovering target's shape and color. Further validation in aquaculture setting indicates the generalization of our method. This work paves the way for outdoor passive imaging applications under challenging scattering conditions.

## Graphical abstract

Light reflected from distant targets is severely attenuated and scattered by dense fog. A color‐channel‐simulated, independently exposed sCMOS array (CISA), combined with a passive colorful de‐scattering pipeline (PCDP), captures and reconstructs extremely weak target signals. Clear color images are recovered under strong scattering, demonstrating a practical route toward passive long‐range imaging in challenging environments.

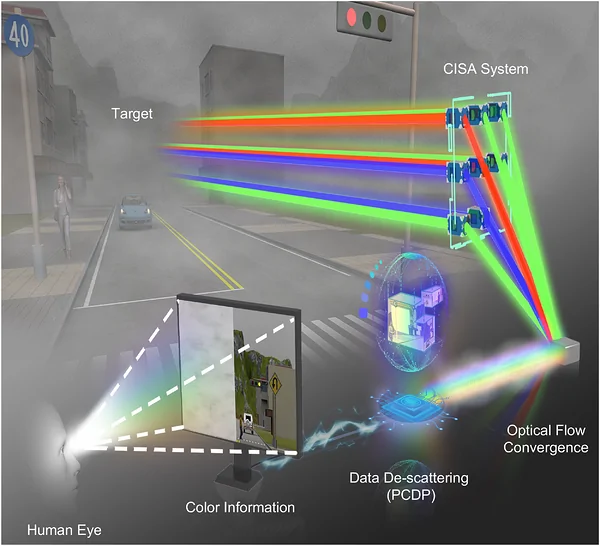

## 1 Introduction

De‐scattering imaging aims to recover target information from images captured through scattering media. Due to its diverse applications in fields such as transportation, remote sensing, emergency rescue, urban security, and deep‐sea exploration, it has become a significant area of focus in optical imaging [1, 2, 3]. As illustrated in Figure 1, typical macro‐scale dynamic scattering media, such as fog, frequently arise under adverse weather conditions. In these conditions, light signals from a target undergo multiple scattering events as they pass through the media, resulting in a rapid loss of photon energy. Consequently, the target's information is obscured by noise, making it nearly impossible for human observers to discern details. Compared to static media like foam and ground glass, the time‐varying characteristics of dynamic media add complexity to the de‐scattering process. In this paper, we focus on addressing the de‐scattering problem in dynamic media, particularly under challenging conditions of strong scattering.

**FIGURE 1:**
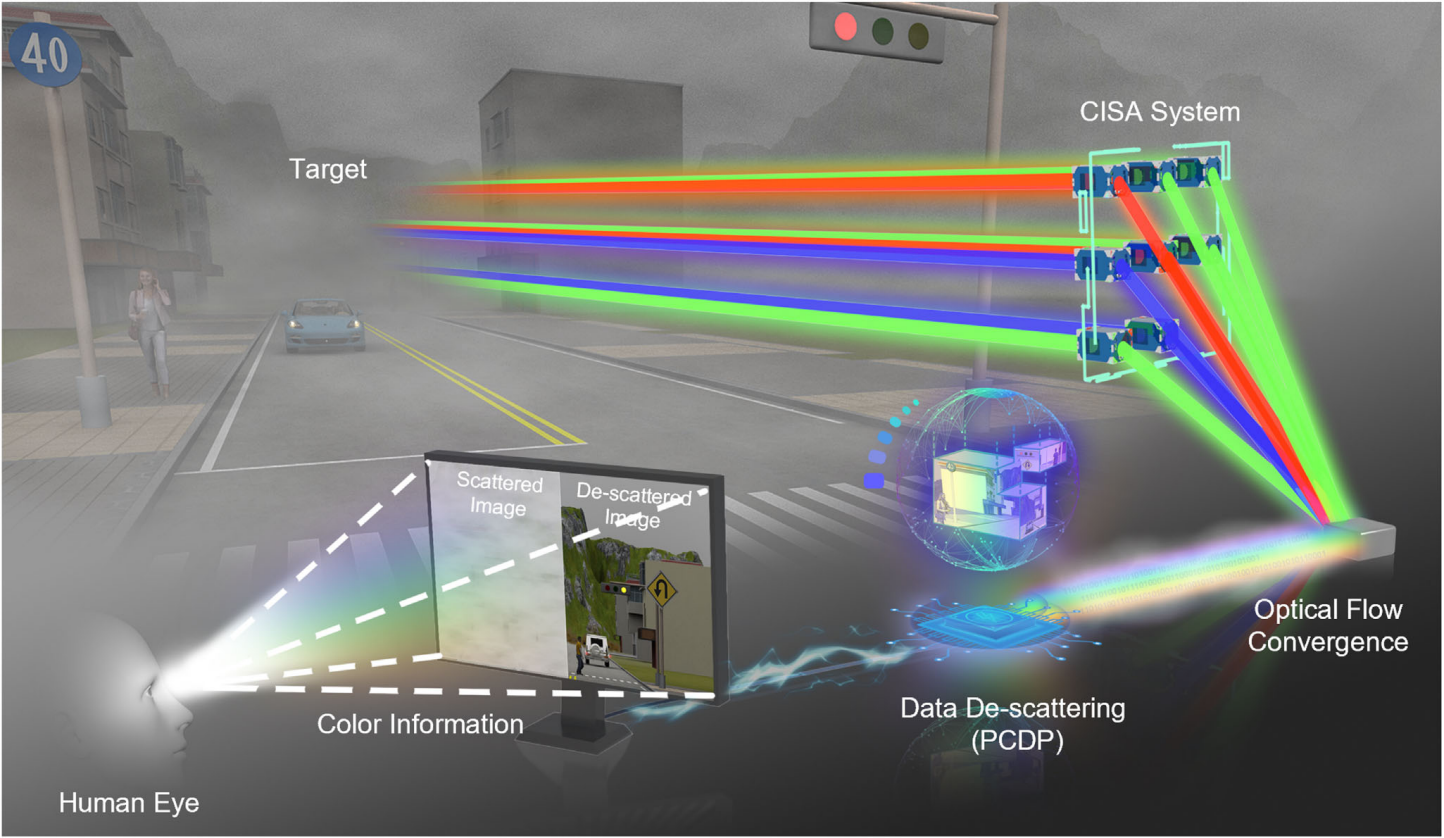
An illustration of de‐scattering in macroscopic scenes. The target signals reflected from distant target, scatter through the dense fog and reach the detection system. Our color channel simulation and independent exposure adjustment mechanisms driven by an sCMOS array (CISA) detection system captures the scattered signal. Along with the proposed passive colorful de‐scattering pipeline (PCDP), we can recover a clear image from the extremely weak signals, making the de‐scattering techniques applicable in practical uses.

In recent years, several types of methods have been proposed to solve the de‐scattering problem, which are broadly categorized as active or passive approaches, depending on whether an active light source is used [4, 5]. Time‐gated imaging is one of the typical active imaging methods. It utilizes laser pulses and time‐gating technology along with high‐performance imaging components, such as single‐photon cameras, to separate photon information at specific depths [6, 7, 8, 9]. Active polarization and polarized LiDAR methods have also been explored for descattering imaging [10, 11, 12]. On the other hand, techniques like millimeter‐wave and terahertz imaging employ long‐wavelength light, which experiences less attenuation in scattering media, thus aiding in target localization [13, 14, 15, 16]. However, complex reflections from surfaces with varying properties may increase spatial broadening of the pulse and introduce artifacts in long‐range imaging. As a result, the stability of these active methods in complex environment still requires further improvement. Additionally, in strong scattering media, due to the rapid attenuation of light energy during propagation, higher laser power is often mandatory, which increases the risk to human eyes [17, 18, 19]. Moreover, using active light sources with only a single or a few wavelengths limits active methods in capturing the full spectral information of the target, making it extremely challenging to restore the target color and other intrinsic matter characteristics.

In contrast, passive imaging techniques do not rely on active light sources, which simplifies system design and is less harmful to human eyes. Passive de‐scattering algorithms are generally categorized into three types: image enhancement methods, hand‐crafted prior‐based methods, and deep learning methods. Image enhancement techniques, such as contrast enhancement and Retinex [20, 21, 22, 23, 24, 25, 26], focus on improving pixel intensity distributions without explicitly estimating the scattering model. Hand‐crafted prior‐based methods, on the other hand, utilize manually designed scattering priors to estimate transmission matrices and atmospheric light intensity. One of the most widely used de‐scattering techniques is the dark channel prior (DCP) [27]. Additionally, Ju et al. introduced the saturation channel prior (SLP) [28], which provides statistically significant depth distribution estimates. More recently, Liu et al. proposed the ROP+ algorithm [29], combining a rank‐one prior with a loss function to iteratively refine the estimation of the parameters of scattering model. Deep learning methods have also demonstrated remarkable results by leveraging scattering datasets and neural networks without requiring an explicit formulation of the physical model or manually designed priors [30, 31, 32, 33, 34, 35, 36]. Moreover, recent advances in passive imaging hardware include polarization imaging devices [37, 38, 39, 40], multispectral data fusion [41], and angular‐selective devices (ASD) [5]. These innovations have further enhanced the capabilities of passive de‐scattering techniques. However, under strong scattering conditions, due to the limited reception of target signals, existing passive approaches still require further improvement.

Despite the progress made by existing methods, some issues heavily hinder the reliability of de‐scattering techniques in real‐world tasks when involving strong scattering media. The first challenge is the inherent complexity of strong scattering scenes. In such scenarios, the target signal becomes very weak, and even slight perturbations in imaging can significantly impact the recovery of target information. Consequently, factors such as ambient lighting variations and lens imperfections can considerably degrade de‐scattering performance in practice. The second challenge is the limited research on extreme scattering conditions. Most existing methods experience significant degradation in image quality under strong scattering. These factors together make de‐scattering under outdoor strong scattering conditions a significant challenge. Furthermore, existing methods still struggle to correctly recover the colorful information of the target under strong scattering condition, while color plays a crucial role in quick identification of target and enhancing scene perception [42, 43, 44], such as identifying traffic signs or performing emergency rescues at sea or in forests during adverse weather conditions.

In this paper, we introduce a passive color imaging framework for real‐world strong scattering media, applicable to dynamic media including dense fog and turbid water. By analyzing the scattering imaging model from the perspective of irradiance, we identify the factors that hinder the practical application of existing methods. Based on these insights, we propose color channel simulation and independent exposure adjustment mechanisms driven sCMOS array (CISA) detection system. In this system, each sCMOS camera is equipped with a specially designed band‐pass filter to realize color channel simulation. Compared with conventional camera, the independent adjustment mechanisms, which is achieved by independently adjusting the exposure of each color channel, enables full‐well photon collection across different spectral bands. Combined with the spatial information from the camera array, this configuration facilitates the generation of simulated Raw‐format scattering images. This system effectively extends the dynamic range, improves the signal‐to‐noise ratio (SNR) of scattering data, and preserves the chromatic identity of the recovered scene. Additionally, by analyzing the characteristics of color space under strong scattering conditions, we frame the de‐scattering challenge as a color modulation problem, and propose a passive colorful de‐scattering pipeline (PCDP) for strong scattering removal. This pipeline enables high‐quality recovery of a target's texture and color by leveraging the fused illumination channel, possessing a high SNR, without relying on scattering attenuation coefficient priors. Outdoor field experimental tests were conducted in the Qinling Mountains, China, where fog data was collected and de‐scattering experiments were performed, demonstrating the system's effectiveness in recovering the shape and color of distant targets. Further validation in real‐world aquaculture settings demonstrates the generalizability of the proposed method. This research paves the way for outdoor imaging applications in challenging scattering environments. Furthermore, we provide the collected data, covering various lighting conditions and scattering media to support future research.

## 2 Results

### 2.1 The Irradiance Imaging Model

After undergoing multiple scattering events and photoelectric conversion, the target's photon signal is recorded as a digital image. The key hardware factors affecting de‐scattering performance are the SNR of the measured target signal and the discriminative limit in distinguishing the target's ballistic light from the background. We analyze this imaging process using a dimensionally consistent irradiance model, and the complete derivation is provided in Section S.1.1.

As discussed by previous works, interference from the target's forward‐scattered light can be ignored [45, 46]. Therefore, for any given pixel on the sensor, the radiance $L_{C}$ reaching the camera can be approximated as the sum of the target‐ballistic term $L_{tar}$, which is the ballistic light from the target that carries its spatially resolvable information, and the path‐radiance term $L_{path}$:

(1) $$L_{C}=L_{tar}+L_{path},$$

As the propagation distance u increases, the ballistic energy decays exponentially, so the target‐ballistic term is expressed as:

(2) $$L_{tar}=L_{obj}\;e^{-OT},\;OT=\int_{0}^{u}\beta \;dz,$$

where $L_{obj}$ is the initial irradiance emitted by the object, β is the attenuation coefficient, and OT is the optical thickness. The path‐radiance term $L_{path}$ collects radiance from the scattering medium and ambient illumination along the propagation path. Under variations in natural outdoor lighting and non‐uniform fog density, $L_{path}$ received by the optical system is not constant, and it raises the overall image brightness without carrying target information.

The reception of the target signal is further affected by optical‐system imperfections. Based on the off‐axis attenuation characteristics of the lens [47], the irradiance E on the sensor can be expressed as:

(3) $$E=\pi L_{C}\cdot \frac{1}{1+4{\left(f/\#\cdot (1-m)\right)}^{2}}\;{\left(\cos\theta \right)}^{4},$$

where f/# is the f‐number and m=1−z/f, with m<0 under typical imaging conditions, so the light field exhibits vignetting. Together with off‐axis deviation, dust on the lens also degrades the recovery of extremely weak target signals in highly scattering environments (see Figure 8b for further analysis).

When the received irradiance is converted into a digital signal, it is influenced by several types of noise, including photon shot noise, dark‐current noise, read noise, and quantization noise [48, 49]. In strong scattering environments with sufficient illumination, shot noise, which follows a Poisson distribution [50], becomes the dominant component [51], allowing a simplified model for further analysis.

Based on the model introduced above, we derive two inequalities to guide the design of the camera system. Since the full‐well capacity limits the exposure time, the target signal remains resolvable only up to a bit‐depth‐governed upper bound of optical thickness:

(4) $$OT_{bit,bound}=\ln\;\left[\frac{\mu_{e,tar}^{\left(0\right)}\;\left(2^{b}-1\right)}{\kappa_{q}\;C_{ADC}}\right],$$

where $\mu_{e,tar}^{\left(0\right)}$ is the reference target‐generated photoelectron count at OT=0, b is the ADC bit depth, $\kappa_{q}$ is a digital‐resolution factor, and $C_{ADC}$ is the ADC full‐scale electron range. Beyond this ADC‐resolvability bound, the optical thickness is also limited by the minimum system SNR required for reliable detection, so the overall detectable optical thickness is set by the more restrictive of the two:

(5) $$OT_{bound}=\min\;\left(OT_{ADC},\;OT_{SNR}\right).$$

The corresponding noise‐limited bound ${\mathrm{OT}}_{\mathrm{SNR}}$ and the full derivation are given in Section S1.1. We further derive an upper bound of the SNR of the fused scattered image:

(6) $$SNR_{sys,max}\approx f\;\sqrt{N_{v}N_{f}C_{use}},$$

where f is the ballistic signal fraction, $N_{v}$ is the number of cameras, $N_{f}$ is the number of fused frames per camera, and $C_{use}$ is the usable charge capacity per exposure. The corresponding noise‐limited optical thickness and the complete derivation are provided in Section S1.1.

According to these results, we conclude that the optical thickness is primarily governed by the system's bit depth, while the SNR of the scattered image is constrained by the equivalent photon capacity $N_{v}N_{f}C_{use}$ and the ballistic fraction f. For passive imaging systems, improving f through hardware modifications is challenging. Therefore, increasing the imaging bit depth and the equivalent photon capacity presents a practical approach to enhancing de‐scattering performance. This insight provides crucial guidance for both the design of imaging systems and the development of algorithms in strong scattering environments.

### 2.2 Color Space Characteristics

Without considering the shot noise, the hue (H), saturation (S) and value (V) domains of HSV color space in scattering scenes exhibits the following properties:

(7) $$\left\{\begin{array}{cc} H_{I} & \approx H_{J}\\ CR_{S_{I}} & \to 1\\ V_{I} & \propto e^{-\beta u} \end{array}\right.,$$

where I represents the scattered image, J denotes the clear image, and K is a constant. CR stands for contrast, which can be expressed as $CR=\frac{I\left(x_{1},y_{1}\right)-I\left(x_{2},y_{2}\right)}{I\left(x_{1},y_{1}\right)+I\left(x_{2},y_{2}\right)}$, where $\left(x_{2},y_{2}\right)$ represents any neighboring pixel of $\left(x_{1},y_{1}\right)$. As indicated in the above equation, the H domain information is preserved, the contrast in the S domain is maintained, and the information in the V domain rapidly diminishes due to scattering.

However, as shown in Figure 2, in strong scattering scenes, shot noise becomes dominant and cannot be ignored. Therefore, considering the photon noise, the SNR characteristics of the scattered image in the HSV space are as follows:

(8) $$SNR_{V_{I}}≳SNR_{S_{I}}≳SNR_{H_{I}},$$

where ≳ denotes approximately greater. The derivation of Equations (7) and (8) is provided in Section S1.2.2.

**FIGURE 2:**
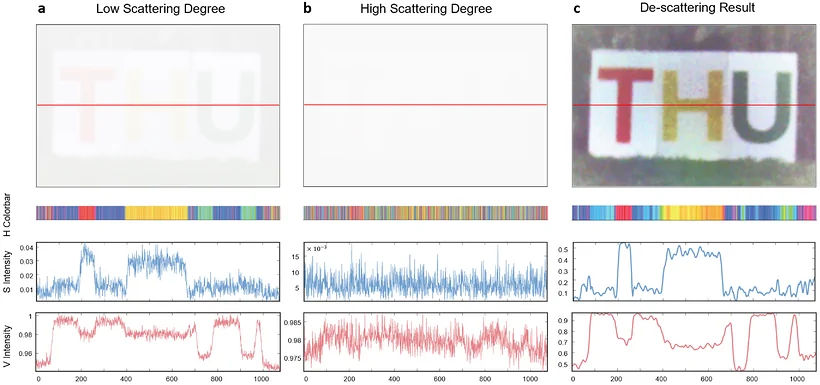
An illustration of the characteristics of the HSV space in scattering scenes. (a–b) The scattered images of the same outdoor scene (the letter “THU”) under varying levels of scattering intensity, along with the HSV distribution of the 230th row in each corresponding scene. As the level of scattering increases, the information in the hue (H) and saturation (S) domains is gradually overwhelmed by noise. The value (V) domain, despite experiencing severe information loss due to fog interference, maintains the highest SNR among all channels. (c) The de‐scattering result of (b) and its corresponding HSV distribution. The noise in the H domain is effectively reduced, while both the S and V domains are significantly enhanced. This enhancement leads to a recovery of the target's contrast and hue direction consistent with the original scene.

According to the above characteristics of the scattered image in strong scattering environments, we can draw the following conclusions. The H domain still retains the original color information of the target, making it useful for identifying the color of objects under dense fog, though it exhibits the lowest SNR. The S domain retains substantial contrast information, making it valuable for identifying object boundaries under dense fog. The V domain maintains the highest SNR. Therefore, the de‐scattering problem in strong scattering environments can be summarized as a task of denoising in the H domain and jointly regulation and enhancing the S and V domains.

### 2.3 The CISA Detection System

In outdoor natural lighting conditions, both the peak spectral energy of sunlight and the peak spectral response of photodetectors are typically concentrated in the green wavelength range. As shown in Figure 3b, in strong scattering scenes, existing CMOS sensors, constrained by a global uniform exposure mechanism, often result in underexposure in the red and blue channels when the green channel reaches full well capacity. This leads to insufficient photon collection in the red and blue channels, resulting in lower SNR. In addition, during the demosaicing process that converts Raw images into RGB images, merging dual green channels reduces noise in the green channel, further widening the SNR gap with the red and blue channels. Taking both the photoelectric conversion process and image signal processor (ISP) processing into account, the low SNR in the red and blue channels limits the ability of subsequent algorithms to accurately restore the target's color.

**FIGURE 3:**
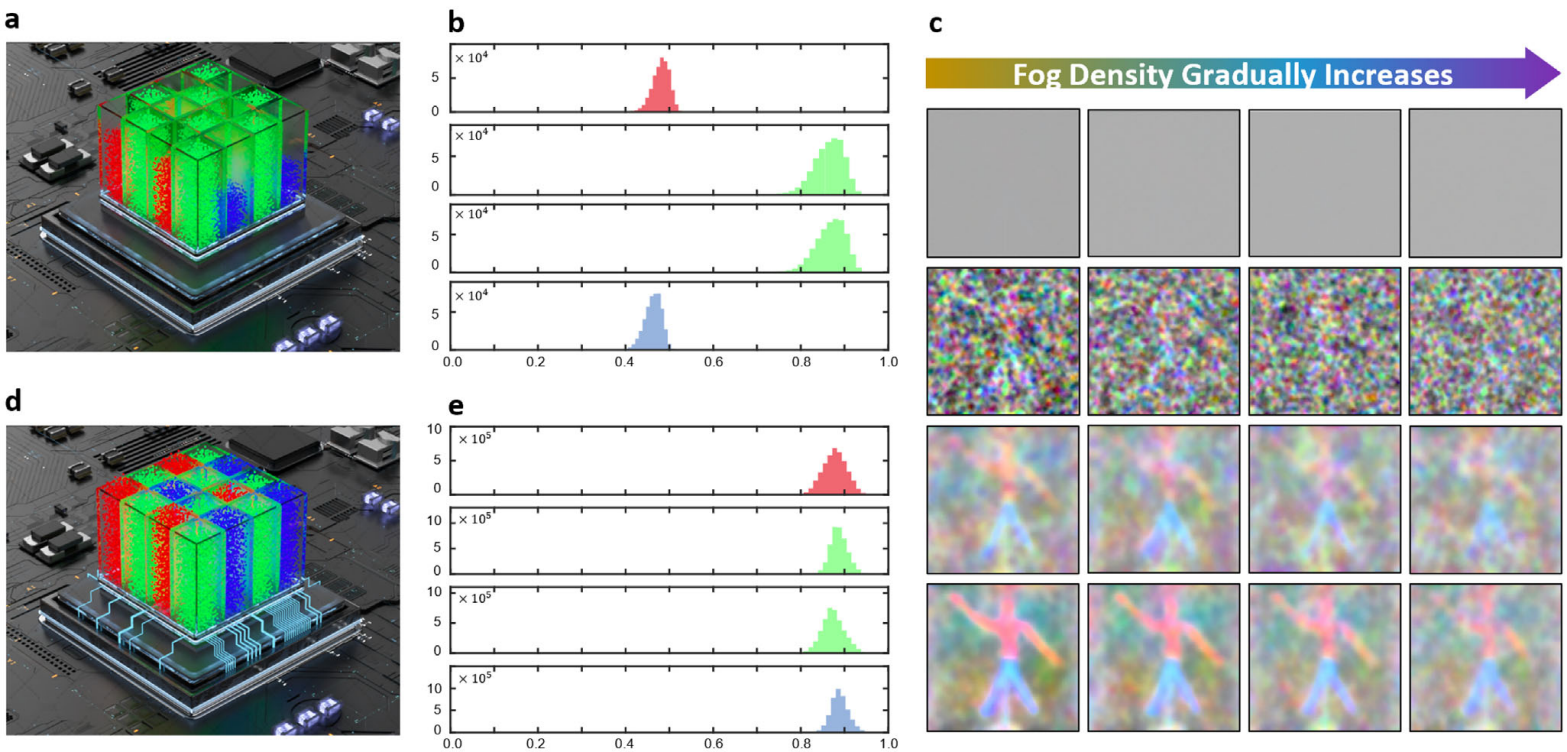
A comparison of the different exposure mechanisms and de‐scattering result. (a) The photon accumulation diagrams for the photodetector under global uniform exposure mechanism. (b) The histogram distribution of each color channel for a CMOS sensor under strong scattering conditions. (c) From left to right, the degree of scattering gradually increases. From top to bottom, the first row shows the original scattered images. The second row displays the de‐scattering results using our PCDP algorithm on a CMOS camera. The third row presents the de‐scattering outcome of one imaging unit in the CISA system using PCDP under a global uniform exposure mechanism. The fourth row shows the de‐scattering result of one CISA imaging unit using PCDP under an independent exposure mechanism, captured at full well capacity. (d) The photon accumulation simulated diagrams of our CISA under independently adjusted exposure mechanism. (e) The histogram distribution of each color channel for our CISA imaging unit under strong scattering conditions.

The CISA system we designed consists of eight high‐performance sCMOS cameras equipped with custom red, green, and blue bandpass filters. Additionally, a CMOS color guide camera is used for comparison. The sCMOS cameras and CMOS camera are arranged in a 3×3 configuration in the sequence RGGB/RGGB/Color. Each sCMOS serves as a simulated color channel, with independently adjustable exposure time and synchronous triggering, ensuring sufficient photon collection across different spectral bands. Calibrated for long‐range imaging, the CISA system provides a combined field of view of approximately 0.6 degrees. In outdoor environments, during continuous day and night standby imaging, the system maintains high‐quality image fusion despite uncontrollable factors like ground vibrations. At an observation distance of 1000 m, the CISA system achieves a fusion size error of less than 2 cm and a viewing angle deviation error of less than 0.3%. More design details can be found in the Methods section and Section S5.

We compare different exposure mechanism and the impact of independent exposure adjustment on de‐scattering performance in Figure 3c. The experiment was conducted in an indoor fog chamber using a CISA system's color imaging unit composed of four sCMOS cameras, with a colorful figure as the target. Under a unified exposure mechanism, the single imaging unit of our CISA system significantly outperforms the CMOS camera, validating the superior performance of our system's imaging unit. Moreover, the independent exposure mechanism yields better results than the unified exposure mechanism, indicating that the traditional exposure mechanism's low SNR in the R and B channels restricts the high‐quality recovery of information in scattering scenes. A quantitative ablation analysis is further provided in Section S10 to separately evaluate the contributions of the reconstruction algorithm, detection platform, and exposure strategy across different scattering conditions.

### 2.4 Details of the Data Processing

As shown in Figure 4a,b, the CISA detection system collects photon signals from each spectral band, approaching full well capacity through an independent exposure mechanism. These signals are then stored as scattering image data. After data acquisition is complete, the system fuses the multi‐frame, multi‐view image data to produce a simulated Raw image. This image is subsequently processed through the ISP to convert it into an RGB‐format scattering image. In this system, each camera functions as a simulated color channel, which is indexed by i, where $i=1,2,\text{…},N_{v}$ and $N_{v}=8$ in this system.

**FIGURE 4:**
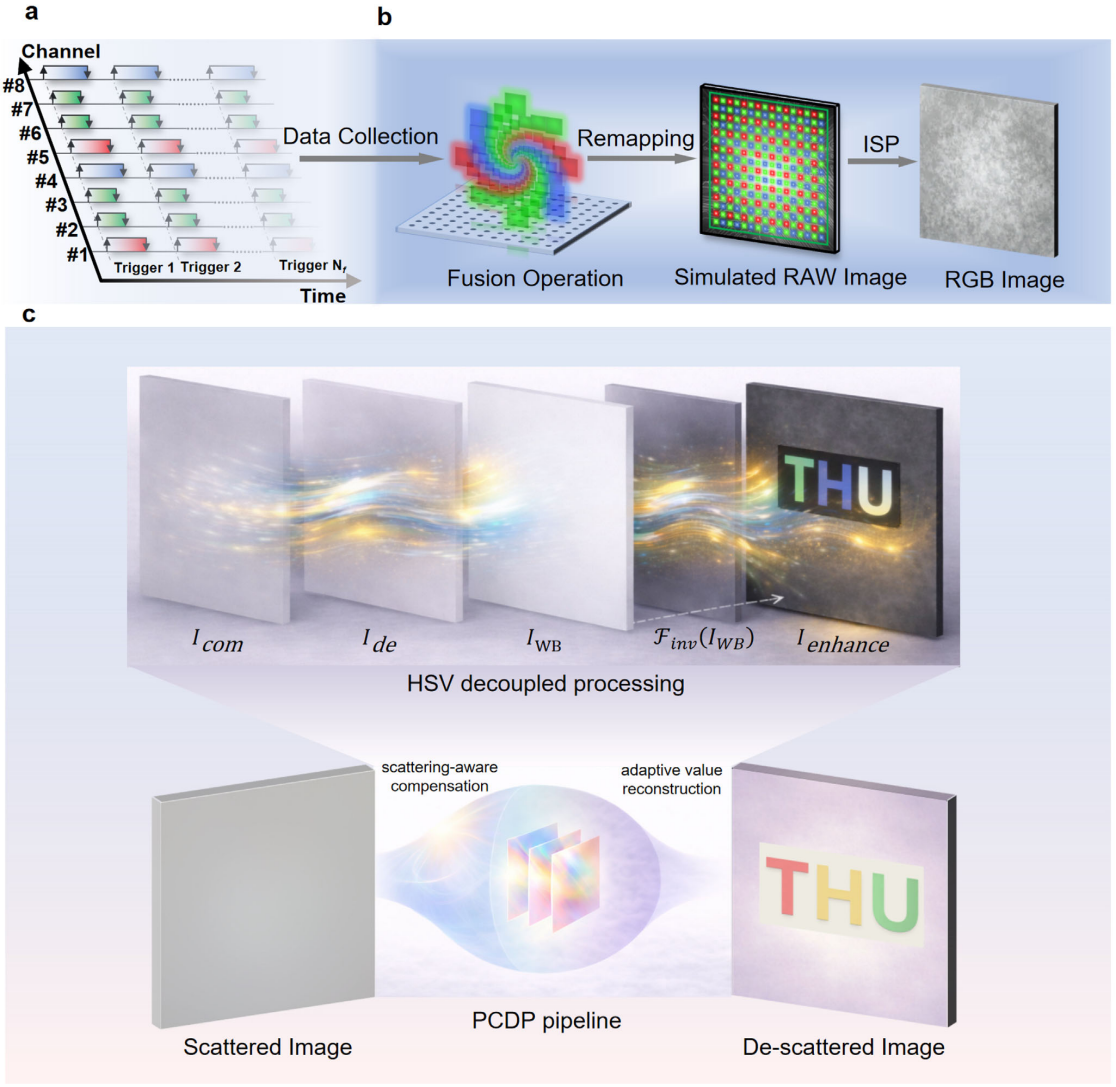
Flowchart of the proposed method. (a) Exposure logic timing diagram of the CISA system. Eight sCMOS cameras are divided into two imaging units, outputting 16‐bit depth TIFF format images at 10 frames per second through independent exposure adjustment. (a) The optical flow convergence processing workflow of the CISA system. Each sCMOS camera, equipped with a bandpass filter for a specific spectral band, functions as a simulated color channel. The image stream data from each camera is fused through a system spatial calibration matrix, producing a simulated Raw image. After being processed by the ISP, it outputs a scattered image in RGB format. (c) An illustration of the proposed PCDP. The fused scattered image data undergoes sequential processing, starting with scattering‐aware illumination compensation. This is followed by denoizing and enhancement in the HSV decoupled processing. Finally, the de‐scattered image is produced by adaptive value reconstruction.

For each camera, $N_{f}$ frames are captured, with each frame denoted as $I_{i}^{\left(k\right)}\left(x,y,\theta_{i}\right)$, where $k=1,2,\text{…},N_{f}$ represents the frame index; by adaptively adjusting the exposure parameter $\theta_{i}$ for different cameras, each color channel is nearly captured at full well capacity. The multi‐frame image fusion $M_{i}$ is then performed to generate a fused single‐channel image:

(9) $$I_{i}\left(x,y\right)=M_{i}\;{\left\{I_{i}^{\left(k\right)}\left(x,y,\theta_{i}\right)\right\}}_{k=1}^{N_{f}}.$$

Through camera view calibration, a fusion matrix $H_{i}$ is generated to achieve spatial alignment of images from different cameras. Then the images from different spectral bands and viewpoints are fused by the operation G. Finally, through the ISP operation $F_{isp}$, the simulated Raw image is converted into an RGB‐format scattering image as:

(10) $$I_{sca}\left(x,y\right)=F_{isp}\;\left(G\;\left({\left\{H_{i}I_{i}\left(x,y\right)\right\}}_{i=1}^{8}\right)\right),$$

which is used for the subsequent de‐scattering pipeline.

As shown in Figure 4c, the proposed de‐scattering pipeline PCDP contains three main steps: scattering‐aware illumination compensation, HSV decoupled processing, and adaptive value reconstruction. First, scattering‐aware illumination compensation is applied to the scattered image, described by the equation:

(11) $$I_{com}=LF_{cal}\left(I_{sca},P_{sys}\right),$$

where $I_{com}$ denotes the compensated image. The operator L represents Legendre polynomial fitting, while $F_{cal}$ refers to the flat‐field correction applied to the image, and $P_{sys}$ denotes the pre‐calibrated illumination‐field distribution within the system's fused field of view, which is an inherent characteristic of the system that can be calibrated using a whiteboard. This step effectively mitigates off‐axis deviations and imaging defects, such as dust, producing images similar to those generated by the atmospheric scattering model (ASM). This aids in overall color control during the following imaging steps.

**FIGURE 5:**
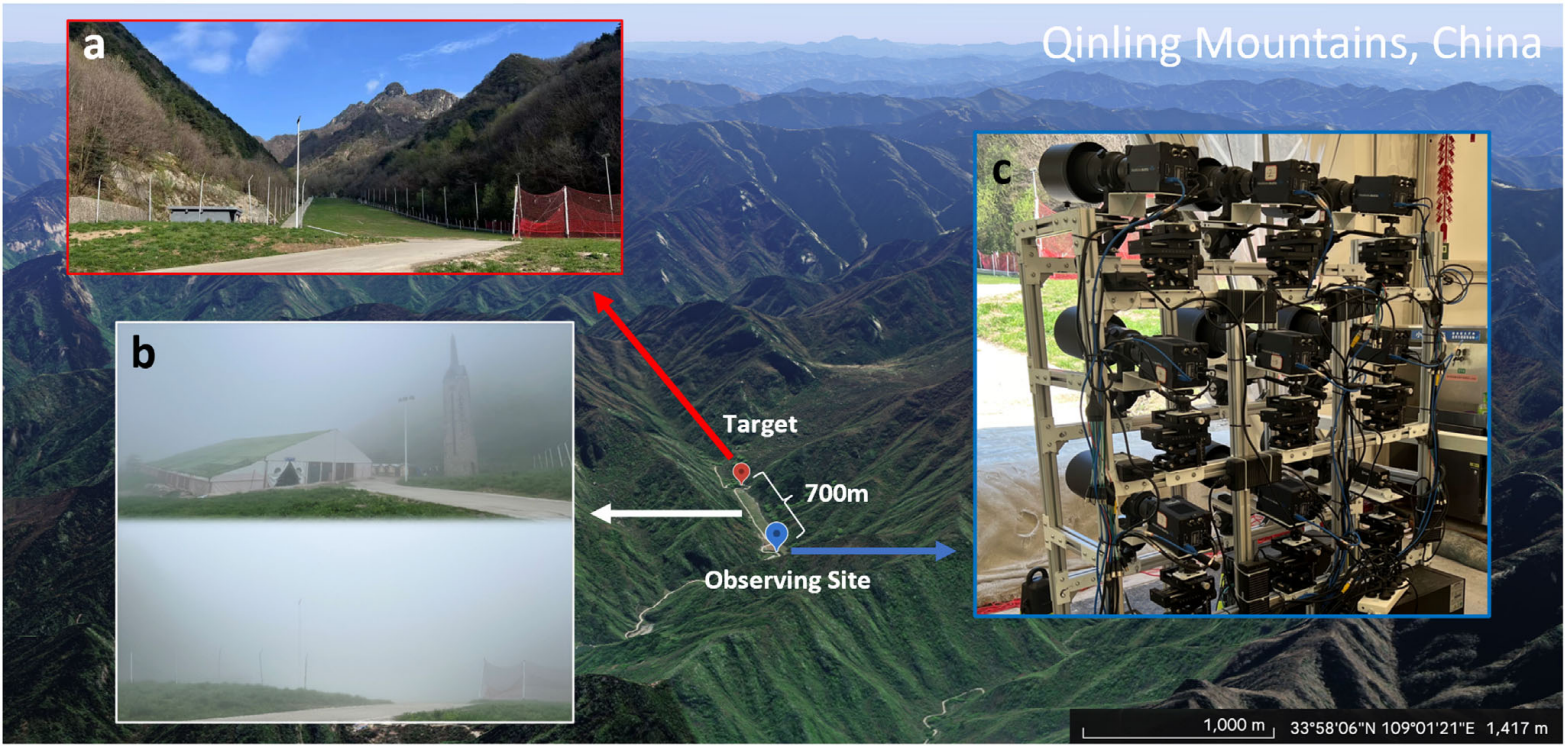
An illustration of the fog testing scenario. (a) High‐altitude mountainous test site. The observation distance is approximately 700 m, with an 8‐degree elevation angle. (b) Overview of the collection and imaging end under heavy fog conditions. (c) The proposed CISA detection system. The camera array consists of 8 high‐performance sCMOS cameras paired with band‐pass filters, jointly controlled by two high‐performance hosts.

The second step is to obtain the HSV decoupled processed image $I_{de}$, which is expressed as:

(12) $$I_{de}=D_{\Omega }\;\left(I_{com},\;W\left(I_{com}\right)\right),$$

where $D_{\Omega }$ represents the denoizing operator applied within the region Ω. The chosen denoizing method is edge‐preserving filtering guided by the fused V domain. W is defined as $W\left(I\right)=0.5\;I_{G}+0.25\;I_{R}+0.25\;I_{B}$, which fuses the three channels of the input image with weighted values. This step effectively suppresses noise in each color channel while preserving edge information, reducing noise in HSV space especially in H and S domains, and preventing the de‐scattered result from appearing color‐inconsistent or dull. As noted in Section S1.3, fusing the image with these weights when captured under the same illuminance yields the highest theoretical SNR. The image $I_{de}$ will be redistributed in the HSV color space to get $I_{WB}$, which will accomplish color correction and chromatic balance. This is then inverted to obtain the dark image $I_{inv}$, the resulting fused luminance channel is subsequently employed to perform V enhancement on $I_{WB}$, producing the enhanced output:

(13) $$I_{enhance}=R\;\left(I_{inv},\;W\left(I_{inv}\right)\right),$$

In the above equation, $I_{inv}=F_{inv}\left(I_{WB}\right)$, the operation $F_{inv}$ refers to the inversion of the normalized image. R enhances the image's V domain based on Retinex theory.

Finally, the adaptive value reconstruction is performed:

(14) $$I_{out}=F_{adj,w}\;\left(F_{inv}\;\left(\frac{1}{L}\;D_{\Omega }\left(I_{enhance}\right)\right)\right),$$

where $I_{out}$ denotes the final output of the proposed pipeline. The operator $F_{adj,w}$ adjusts the overall V domain of the image with parameter w, L controls the de‐scattering enhancement. The mechanism described in Equation (14) adjusts the HSV space as follows:

(15) $$\left\{\begin{array}{cc} H_{J} & =H_{I}\\ S_{J} & =\frac{V_{I}}{V_{I}-\left(1-\frac{L}{T}\right)}S_{I}\\ V_{J} & =1-\frac{T}{L}\left(1-V_{I}\right) \end{array}\right.$$

It is similar to inversion methods which are based on prior models. The key idea is to keep the H domain unchanged, while using the pixel gain coefficient (T) matrix from the Retinex algorithm under low‐light conditions to enhance both S and V domains simultaneously. The detailed derivation of Equation (15) can be found in Section S.2.

### 2.5 Experimental Results

All outdoor de‐fogging experiments were conducted in a valley of the Qinling Mountains, China, at an altitude of approximately 1400 m, with an observation distance of 700 m, as shown in Figure 5. This region is known for its humid climate and frequent foggy days in inland China. The tests took place from April to May in 2024. The imaging section of the CISA system comprised eight sCMOS cameras with a 16‐bit depth, with details available in the Methods section and Supplementary Information.

With the outdoor measurements, we evaluate the effectiveness of the proposed method. In Figure 6, we present the de‐scattering results of traffic signs under various scattering conditions, visibility under the fog from 700 m to 200 m. Each traffic sign, with a diameter of approximately 1 m, was chosen to simulate a foggy traffic scenario. As illustrated in the second row, traditional high‐performance CMOS cameras struggle to restore the original target features. When the fog is relatively light, existing algorithm can recover some target information from scattered image obtained by CMOS; however, the overall image quality remains poor, making it difficult to discern even the red STOP sign. In contrast, our designed CISA system, combined with the same algorithm as used by the second row, can fully preserve the target information despite the overall de‐scattering effect still being imperfect. By further leveraging the proposed pipeline, texture details and colors are effectively recovered, even when the OT under fog reaches its maximum value of 10.5. To quantitatively evaluate color preservation under fog, we further compared the recovered traffic‐sign colors with their clear‐condition references across four optical thicknesses (Section S9). Color preservation was characterized using hue‐angle deviation and chroma retention. PCDP consistently achieved the smallest hue deviation across all tested scattering conditions, demonstrating its improved ability to preserve the chromatic identity of the targets.

**FIGURE 6:**
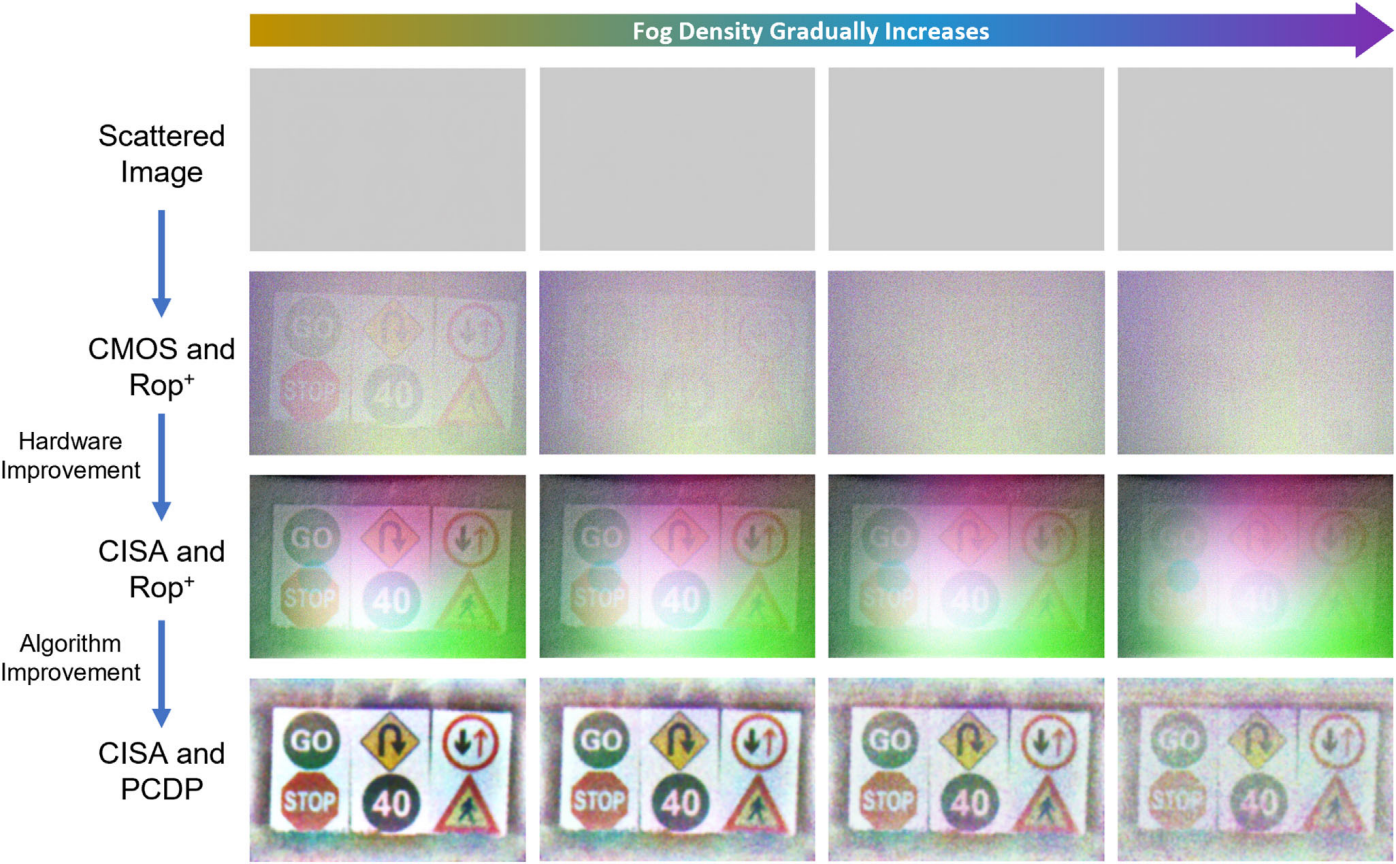
De‐scattering results of planar targets under strong scattering conditions. The first row presents the unprocessed scattering images of traffic signs captured with the fog density increasing gradually from left to right. The second row illustrates the de‐scattering results obtained by applying the ROP+ algorithm to multi‐frame raw images captured by a CMOS camera. The third row shows the de‐scattering performance of the same algorithm when applied to CISA system, highlighting the comparison between the two devices. The last row displays the de‐scattering results achieved by applying the proposed PCDP framework to the CISA system, demonstrating a significant improvement in visibility and detail restoration.

The de‐scattering results of a more complex scene are presented in Figure 7. To simulate a rescue scenario in mountainous foggy weather, three volunteers posed in front of a rockery, each wearing clothing of different colors. This setup increases the complexity due to the varying visibility and texture contrast of the targets under dense fog conditions. According to the results, the proposed pipeline is still able to faithfully recover the texture and color information of the targets, demonstrating its effectiveness in challenging real‐world applications. These findings highlight the potential of the proposed system for practical use in scenarios such as search and rescue operations in foggy environments. De‐scattering results compared with other algorithms are provided in the Supplementary Information. To assess how the recovered images benefit downstream vision tasks, we overlay each result with the detection produced by a COCO‐pretrained YOLO26m detector. Only the CISA + PCDP output enables the detector to localize all targets with high confidence, whereas the CMOS baseline leaves most targets undetected under strong scattering, confirming the practical value of our system for tasks such as search and rescue. A full quantitative evaluation is provided in Section S9.4.

**FIGURE 7:**
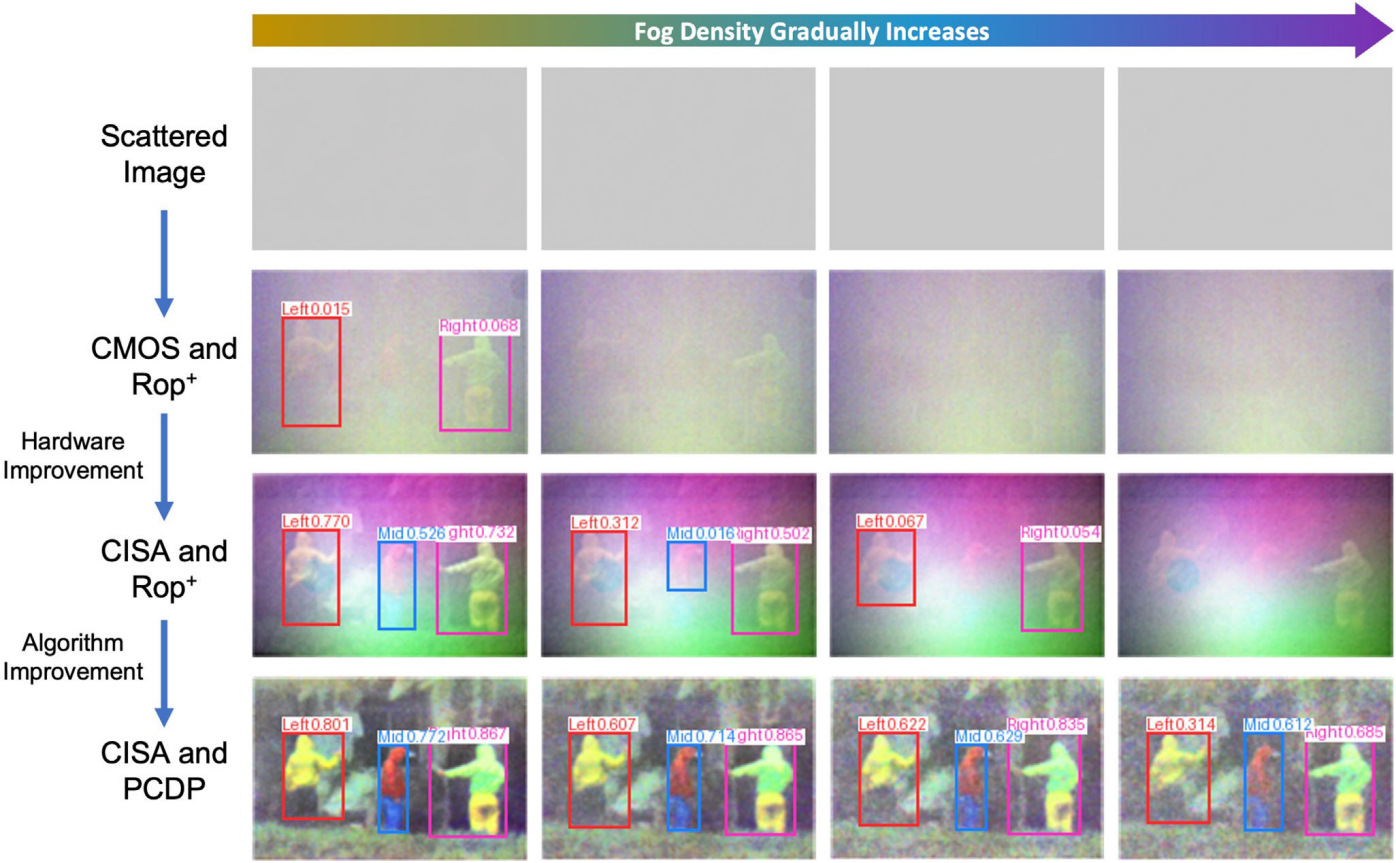
De‐scattering results of stereo targets under strong scattering conditions. The first row presents the unprocessed scattering images of human postures captured with the fog density increasing gradually from left to right. The second row illustrates the de‐scattering results obtained by applying the ROP+ algorithm to multi‐frame raw images captured by a CMOS camera. The third row shows the de‐scattering performance of the same algorithm when applied to our CISA system. The last row demonstrates the de‐scattering results of our PCDP method, which significantly enhances the visual quality by improving image color and restoring finer details. Each panel is overlaid with the bounding box and confidence score produced by a COCO‐pretrained YOLO26m detector, illustrating how the improved image quality translates into more reliable downstream person detection.

## 3 Discussion

Building upon the refined irradiance theory for strong scattering scenarios, we have developed CISA, a novel detection system composed of sCMOS array that can generate high‐quality simulated Raw images. By incorporating the PCDP, a de‐scattering pipeline informed by the analysis of color space characteristics in such environments, we realize passive de‐scattering with high quality color reconstruction. This advancement paves the way for real‐world imaging applications under strong scattering conditions.

Compared to gate‐based or single‐photon camera systems, our system operates independently of active light sources and does not require prolonged scanning. It employs an imaging mechanism similar to conventional CMOS cameras, enabling straightforward and reliable detection of target's textures and color information. Moreover, its full compatibility with most existing de‐scattering algorithms greatly enhances the adaptability and recovery capabilities of precision instruments in challenging natural environments.

Instead of relying on the traditional ASM, we utilize comprehensive light field correction to achieve an image information distribution similar to ASM. This approach allows us to optimize overall image restoration by focusing on the illumination domain with the highest SNR, effectively compensating for H domain noise while restoring S and V domains. The entire process is highly interpretable and consistently delivers stable restoration performance, providing robust technical support for de‐scattering imaging under real‐world adverse weather conditions.

### 3.1 System Resolution Evaluation and Ablation Experiments

To evaluate the resolution performance of the proposed pipeline, we conduct a resolution test at a distance of 700 m and present the de‐scattering results of a resolution chart in Figure 8a. The grid pattern resolution chart features a minimum line width of 2 cm, while the circular resolution chart exhibits an angular separation of 22.5 degrees and a diameter of 1 m. The results indicate that, prior to reaching the limit of OT, the resolution of the de‐scattered target remains approximately 2 cm.

**FIGURE 8:**
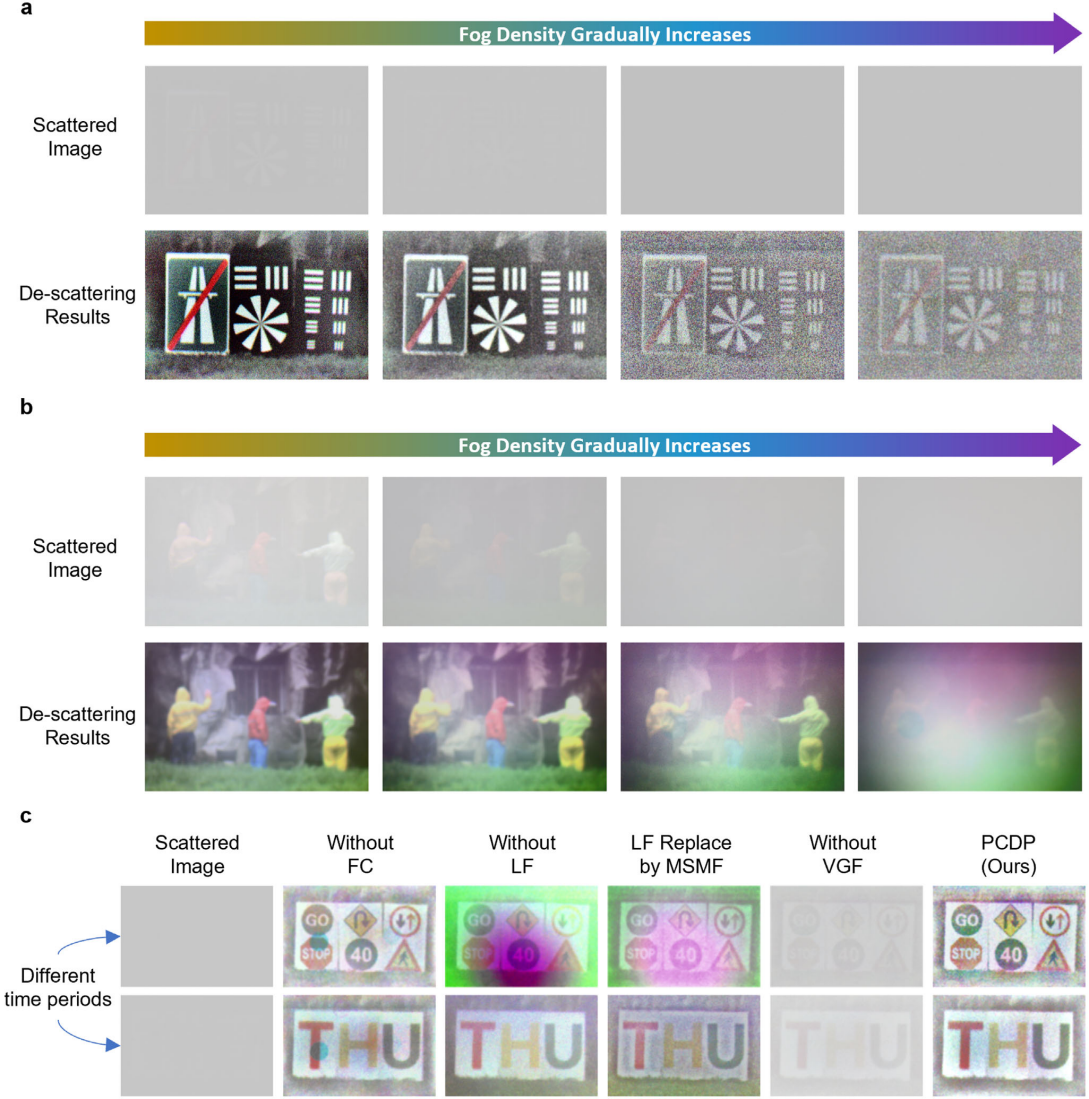
Extended Testing of the CISA system. (a) Resolution test of the proposed pipeline. The fog density increased gradually from left to right. The proposed pipeline can provide de‐scattered images with a resolution of approximately 2 cm under strong scattering conditions. (b) The impact of lens performance on de‐scattering imaging. The first row is the scattered image. The second row shows the de‐scattering results without using our proposed light field comprehensive correction module. (c) Ablation test of the proposed pipeline. The top and bottom rows represent de‐scattering results under different outdoor time periods. In each column, from left to right, the images show the original scattering image followed by de‐scattering results after sequentially removing each processing step from the pipeline. FC: Flatfield Correction, LF: Legendre polynomial fitting, MSMF: Multi‐Scale Mean Filtering and Fitting, VGF: V‐Guided Filtering. The results demonstrate that each step of our proposed method is crucial in addressing complex scattering conditions.

In Figure 8b, we analyze the impact of off‐axis aberrations and dust on the lens, factors that are unavoidable in outdoor scenes, on the de‐scattering performance. When compared to Figure 7, under low scattering conditions, these factors have a minimal effect on de‐scattering enhancement. However, as the scattering level increases, their impact becomes more significant, highlighting the importance of our proposed light field comprehensive correction module. Conventional detectors, such as CMOS sensors, also exhibit these effects when using de‐scattering algorithms. However, under conditions of such intense scattering, the images captured by CMOS sensors are often dominated by noise, which obscures these effects and makes them less noticeable.

Additionally, we conduct an ablation study on the proposed pipeline under different outdoor sun lighting conditions. As shown in Figure 8c, each step plays a crucial role in enhancing de‐scattering quality, and the removal of any step results in a noticeable degradation of the de‐scattering results, underscoring the necessity of the complete pipeline. In the Section S10, we provide further details of the ablation study to demonstrate the effectiveness and generalizability of the proposed pipeline.

### 3.2 System Generalization Evaluation

To evaluate generalization across scattering media and sensor architectures, we implemented the proposed pipeline on a miniaturized CMOS camera array and tested it in turbid water. By using farmed carp to simulate real turbid water, we avoided the distortion of scattering characteristics, which often occurs when using skimmed milk or lime. Miniaturized camera array is used to conduct de‐scattering tests on colored objects and fish under both bright and dark lighting conditions. According to the results shown in Figure 9a, the proposed pipeline exhibits significant advantages over previous algorithms, demonstrating the effectiveness of the proposed method. As further illustrated in Figure 9b and the video contained in the Supplementary Materials, de‐scattered video sequences of freely swimming fish under strong scattering conditions are presented, demonstrating that the proposed miniaturized system supports video‐rate acquisition with offline reconstruction for observing dynamic targets in turbid aquatic media. The underwater experiments include both daylight illumination and continuous artificial illumination. They are therefore used to evaluate generalization across scattering media and sensor architectures, rather than as standalone evidence for passive operation.

**FIGURE 9:**
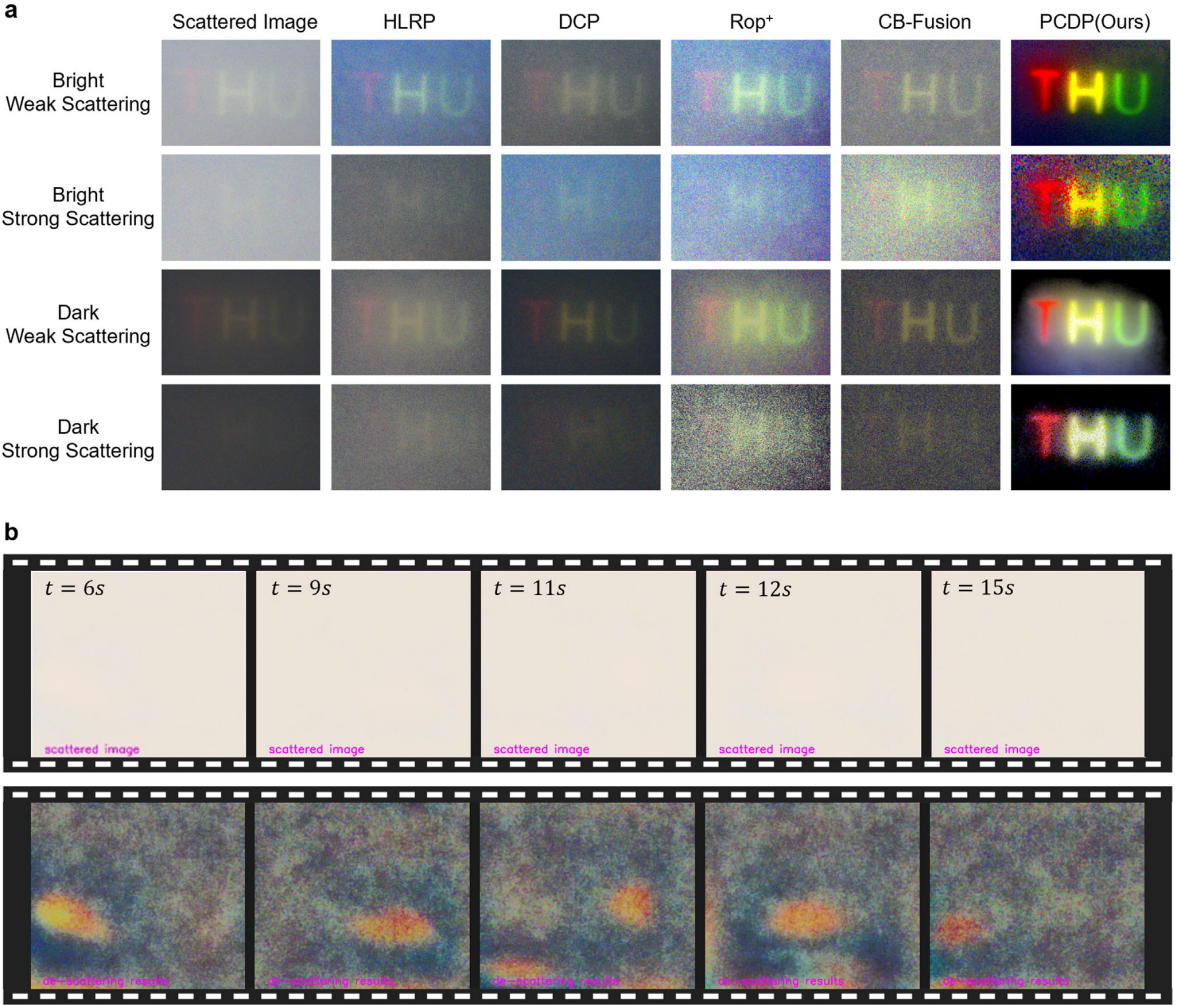
Comparison of underwater de‐scattering performance of different methods. (a) Comparison with existing methods. From left to right, the columns present the original scattering images followed by the de‐scattering results of different methods: HLRP, DCP, ROP+, CB‐Fusion [52], PCDP. The first row represents a weak scattering scenario under normal lighting conditions, while the second row shows a strong scattering scenario under the same lighting. The third row depicts a weak scattering scenario with underexposed lighting, and the last row illustrates a strong scattering scenario with underexposed lighting. The proposed method effectively recovers target information, including contours and details, under varying lighting and scattering conditions, demonstrating its robustness and versatility in complex underwater environments. (b) De‐scattering results of different frames. Miniaturized imaging system was developed for dynamic observation and visualization of red carp in turbid aquatic environments.

The limitation of the proposed pipeline primarily lies in de‐scattering with large depth range. The CISA detection system requires an accurate camera fusion matrix for field‐of‐view fusion, making it challenging to achieve precise observations of multiple objects at different depths simultaneously. However, in practice, we may be more interested in high‐value targets at specific depths. Additionally, since the CISA detection system consists of an array of cameras that naturally allows for accurate depth recovery through the multi‐view camera mechanism, this limitation can be mitigated by adjusting the parameters of the fusion matrix in the future. Another limitation concerns the reconstruction of dynamic targets. Multi‐frame fusion introduces a trade‐off between noise suppression and temporal fidelity, and faster target motion may lead to ghosting or motion blur over the fusion window. Although moving targets were successfully reconstructed in the underwater experiments, further improvements will require motion‐compensated registration and adaptive fusion‐window selection.

Moreover, the system can be configured with filters across multiple spectral bands, enabling it to capture fine spectral data over a wide range of wavelengths. This lays the foundation for the CISA detection system to achieve hyperspectral imaging, with potential applications in fields such as remote sensing, surveillance, agriculture, and hydrology.

## 4 Methods

### 4.1 Details of the CISA Detection System

The camera array system consists of eight high‐bit‐depth sCMOS cameras, a synchronization trigger box, two high‐performance hosts, and a portable power supply. The sCMOS cameras are Teledyne Photometrics Prime BSI, featuring a resolution of 2048×2048 pixels, 95% quantum efficiency, and a 16‐bit data output. These cameras also offer a full well capacity of 54 000 $e^{-}$ and a maximum frame rate of 43 frames per second. The CMOS camera is an FLIR ORX‐10G‐310S9C model, with a resolution of 6464×4852 pixels, 12‐bit depth, and a full well capacity of 10 575 $e^{-}$. Its maximum frame rate is 26 frames per second. Both types of cameras are equipped with 500 mm lenses (SIGMA F4 DG OS HSM) and 2× teleconverters. The system operates at a maximum frame rate of 30 frames per second. For simultaneous triggering, an OF‐P‐SYNC camera synchronizer is used. Data transmission is handled by the PCIe‐USB340 acquisition card, ensuring fast and stable performance across the array. The cameras are mounted on a custom aluminum frame, with 20 cm spacing between each unit. The main structure measures 1000×1000×1800 mm. Each camera base is installed on a high‐precision, three‐degree‐of‐freedom sliding rail for precise position adjustments. Calibration is conducted over long distances, and the data acquisition software includes Spinview and LUSTER software by LUSTER LightTech.

### 4.2 Details of the Experiments

We evaluated the system's de‐scattering performance and operational stability in heavy fog conditions in the valleys and roads on the northern slopes of the Qinling Mountains in China. As shown in Figure  5, the valley testing was conducted in the Cui‐Hua Mountain Landslide National Geopark. The test distance was 700 m, at an altitude of approximately 1400 m, with an observation angle of 8 degrees. As illustrated in Section S4, the fusion accuracy test of CISA was performed at Taiping National Forest Park, with a test distance of up to 1000 m. Testing took place at ambient temperatures ranging from $-20^{\circ }C$ to $30^{\circ }C$, with the system operating continuously day and night in adverse weather conditions. Data collection occurred during heavy fog conditions, managed by the acquisition software.

For the controlled experiment, indoor tests (Figure 3) were conducted in a fog chamber with dimensions of 1.5 m × 0.5 m × 0.5 m. The chamber was made of high‐transparency glass. Different fog densities were generated using a 1.7 MHz ceramic atomizer, simulating a water mist environment dominated by Mie scattering. Two fans were used to ensure the uniform distribution of fog within the chamber. The observation target was placed inside the chamber.

In the comparisons of foggy scenes, the frame rates and acquisition durations of the CMOS camera and the CISA system were kept consistent. The CMOS camera captured 10 continuous 12‐bit RAW images, which were fused and processed through an ISP to produce de‐scattered double RGB format images. Similarly, each sCMOS camera in the array system captured 10 continuous 16‐bit TIFF images. These images were fused using a calibration matrix to generate a simulated RAW format image, which was then processed by the ISP to yield double RGB format images.

In the miniaturized turbid water testing system, the main components include a high‐transparency acrylic aquarium with dimensions of 1.7 m × 0.7 m × 0.8 m. The system is equipped with nine FLIR BFS‐U3‐31S4C cameras, each with a 12‐bit depth. These cameras use FA0802D lenses with an 8 mm focal length and 2/3'' sensors. The cameras are spaced approximately 8 cm apart and are supported by side‐front lighting. To simulate a realistic underwater testing environment, we introduced several koi fish, creating a turbid water condition with scattering media of varying particle sizes. The cameras are spaced approximately 8 cm apart. Data were acquired under two illumination conditions to evaluate the robustness of the method to changes in illumination. In the bright condition, a broadband side‐front spotlight was switched off and the tank was illuminated only by daylight entering the laboratory. In the dark condition, the laboratory was blacked out and the same spotlight, run continuously and unsynchronized with camera acquisition, was the sole illumination source. Exposure, gain, and processing settings were held fixed across both conditions.

In the underwater experiments, each camera in the array system captured 10 continuous 12‐bit RAW images. These images were fused using a calibration matrix to produce a synthetic RAW image. The synthetic image was then processed by the ISP to generate double RGB format images. In the method comparison tests, the RGB format scattering images were derived from single‐frame RAW images captured by a central perspective camera.

Further details of the experimental sites, acquisition conditions, and hardware configuration are provided in the Supplementary Information, together with the engineering specifications of the imaging system. The implementation of the PCDP pipeline, including its main processing stages, computational complexity, and measured processing performance, is also described in detail in the Supplementary Information.

## Author Contributions

X.J. conceived and supervised the research. R.W. designed the detailed implementations. R.W., X.W. and Z.Y. designed and set up the detection system and conducted the experiments. R.W. developed the code, performed data analysis, and created the figures for the paper. R.W., J.W. and X.W. carried out the theoretical derivations. R.W. and J.W. wrote the manuscript with contributions from all authors.

## Funding

National Natural Science Foundation of China, Grant Numbers: 61827804 and 62131011.

## Conflicts of Interest

The authors declare no conflicts of interests.

## Supporting information


**Supporting File 1**: advs77876‐sup‐0001‐SuppMat.pdf.

**Supporting File 2**: advs77876‐sup‐0002‐VideoS1.mov.

## Data Availability

The data that support the findings of this study are available from the corresponding author upon reasonable request.
