# Supplementary material for: Passive Color Imaging Through Macroscopic Strong Scattering Media
Source: Adv Sci (Weinh). 2026 Oct 29:e77876. Online ahead of print. doi: 10.1002/advs.77876 (PMC13720266; doi:10.1002/advs.77876)
Supplement: Supplementary file 1 [file ADVS-9999-e77876-s002.pdf]

# Supplementary Materials for
# Passive Color Imaging Through Macroscopic Dynamic Strong Scattering Media

Renrui Wang[1,4], Xiaoyu Wang[2], Jianyu Wang[3], Zhiyu Yang[1], Xiangyang Ji[2,*]

[1]Shenzhen International Graduate School, Tsinghua University, Shenzhen 518055, PR China.

[2]Department of Automation, Tsinghua University, Beijing 100084, PR China.

[3]Yau Mathematical Sciences Center, Tsinghua University, Beijing 100084, PR China.

[4]Department of Electrical and Computer Engineering,

National University of Singapore, Singapore 117583, Singapore.

[*]Corresponding author: E-mail: xyji@tsinghua.edu.cn

# S.1 Analysis of scattering theory under strong scattering condition

## S.1.1 Analysis of the Imaging Model under Strong Scattering Conditions

This section develops a dimensionally consistent imaging model for passive target detection through strong scattering media. Rather than predicting a universal detection limit for every scattering scene, the model describes how spectral radiance, sensor irradiance, exposure, incident photon number, generated photoelectron number, full-well capacity, ADC bit depth, conversion gain, and digital output are related.

**Target-ballistic and path-radiance components.** We separate the spectral radiance arriving at the camera into target-ballistic and path-radiance components (*1, 2*):

$$L_{\mathrm{C},\lambda} = L_{\mathrm{tar},\lambda} + L_{\mathrm{path},\lambda}, \tag{S.1}$$

where $L_{\mathrm{C},\lambda}$, $L_{\mathrm{tar},\lambda}$, and $L_{\mathrm{path},\lambda}$ are measured in $\mathrm{W\,m^{-2}\,sr^{-1}\,nm^{-1}}$. Here, $L_{\mathrm{tar},\lambda}$ is the target-ballistic radiance that retains spatially resolvable target information, whereas $L_{\mathrm{path},\lambda}$ contains radiance from the scattering medium and ambient illumination.

The target-ballistic component decreases with optical thickness according to

$$L_{\mathrm{tar},\lambda} = L_{\mathrm{obj},\lambda} \exp[-OT(\lambda)], \tag{S.2}$$

where

$$OT(\lambda) = \int_0^u \beta(\lambda, z)\, dz. \tag{S.3}$$

Here, $\beta(\lambda, z)$ is the extinction coefficient in $\mathrm{m^{-1}}$, so $OT(\lambda)$ is dimensionless (*3*).

As in the main text, interference from the target's forward-scattered light is treated as negligible in this regime, so the target-ballistic term $L_{\mathrm{tar},\lambda}$ is taken to consist of unscattered ballistic photons; photons scattered by the medium, including any forward-scattered contribution, are assigned to $L_{\mathrm{path},\lambda}$. We examine this assumption directly in Sec. S.3.6.

**From spectral radiance to spectral exposure.** For color channel $c \in \{R, G, B\}$, component $j$ produces the following spectral irradiance at the sensor:

$$E_{j,c,\lambda} = \Omega_{\text{eff},c}(\theta, \lambda) T_{\text{lens}}(\lambda) T_{\text{filter},c}(\lambda) L_{j,\lambda}, \qquad j \in \{\text{tar}, \text{path}\}, \tag{S.4}$$

where the quantities are defined as follows:

- $E_{j,c,\lambda}$ is the spectral irradiance in $\mathrm{W\,m^{-2}\,nm^{-1}}$;
- $\Omega_{\text{eff},c}(\theta, \lambda)$ is the effective collection solid angle in $\mathrm{sr}$, including aperture, magnification, off-axis attenuation, and vignetting;
- $T_{\text{lens}}(\lambda)$ is the dimensionless lens transmission;
- $T_{\text{filter},c}(\lambda)$ is the dimensionless transmission of the bandpass filter in channel $c$.

In a simplified thin-lens model, the off-axis dependence contained in $\Omega_{\text{eff},c}$ includes the conventional $\cos^4 \theta$ irradiance falloff (*4*).

Over an exposure time $t_c$, the spectral exposure at the sensor is

$$H_{j,c,\lambda} = t_c E_{j,c,\lambda}, \tag{S.5}$$

where $H_{j,c,\lambda}$ has units of $\mathrm{J\,m^{-2}\,nm^{-1}}$.

The radiometric and sensor-domain conversion chain can then be written as

$$L_\lambda \longrightarrow E_\lambda \longrightarrow H_\lambda \longrightarrow N_\gamma \longrightarrow \mu_e \longrightarrow D, \tag{S.6}$$

where $N_\gamma$ is the incident photon number, $\mu_e$ is the mean generated photoelectron number, and $D$ is the digital output in digital numbers (DN).

**Incident photon number and generated photoelectrons.** The expected number of target-ballistic photons incident on one pixel during exposure time $t_c$ is

$$N_{\gamma,\text{tar},c} = A_{\text{pix}} \int H_{\text{tar},c,\lambda} \frac{\lambda}{hc_0} \, d\lambda, \tag{S.7}$$

and the corresponding number of path-radiance photons is

$$N_{\gamma,\mathrm{bg},c} = A_{\mathrm{pix}} \int H_{\mathrm{path},c,\lambda} \frac{\lambda}{hc_0}\, d\lambda. \tag{S.8}$$

Here, $A_{\mathrm{pix}}$ is the pixel area in $\mathrm{m}^2$, $h$ is Planck's constant, and $c_0$ is the speed of light. The quantities $N_{\gamma,\mathrm{tar},c}$ and $N_{\gamma,\mathrm{bg},c}$ are dimensionless photon counts.

Only a fraction of the incident photons generate collected photoelectrons. The wavelength-dependent quantum efficiency $QE(\lambda)$ gives this probability (5). The expected target-ballistic photoelectron count is

$$\mu_{e,\mathrm{tar},c} = A_{\mathrm{pix}} \int H_{\mathrm{tar},c,\lambda} QE(\lambda) \frac{\lambda}{hc_0}\, d\lambda, \tag{S.9}$$

and the expected background photoelectron count is

$$\mu_{e,\mathrm{bg},c} = A_{\mathrm{pix}} \int H_{\mathrm{path},c,\lambda} QE(\lambda) \frac{\lambda}{hc_0}\, d\lambda. \tag{S.10}$$

Both $\mu_{e,\mathrm{tar},c}$ and $\mu_{e,\mathrm{bg},c}$ are measured in electrons, denoted by $\mathrm{e}^-$.

More generally, the incident photon number and generated photoelectron number are related by

$$\mu_{e,j,c} = \int QE(\lambda)\, dN_{\gamma,j,c}(\lambda), \qquad j \in \{\mathrm{tar}, \mathrm{bg}\}. \tag{S.11}$$

For a sufficiently narrow spectral channel, this relation becomes

$$\mu_{e,j,c} \approx \overline{QE}_c N_{\gamma,j,c}, \tag{S.12}$$

where $\overline{QE}_c$ is the photon-weighted effective quantum efficiency of channel $c$.

The total signal-generated photoelectron count is

$$\mu_{e,c} = \mu_{e,\mathrm{tar},c} + \mu_{e,\mathrm{bg},c}. \tag{S.13}$$

The sensor full-well capacity constrains the photoelectron count rather than the incident photon count, so the sensor-noise and ADC models are written in the photoelectron domain.

**Target-ballistic photoelectron fraction.** After quantum-efficiency weighting, we define the target-ballistic photoelectron fraction as

$$f_c = \frac{\mu_{e,\mathrm{tar},c}}{\mu_{e,c}}. \tag{S.14}$$

Thus,

$$\mu_{e,\mathrm{tar},c} = f_c \mu_{e,c}. \tag{S.15}$$

Thus, $f_c$ is the fraction of generated photoelectrons contributed by target-ballistic light.

Under strong scattering, path radiance may keep the total image intensity high even when $f_c$ is very small. Image brightness alone does not determine whether the target can be detected.

**Photoelectron statistics and sensor noise.** For statistically independent incident photons, photoelectron generation follows Poisson statistics (*6, 7*). The target and background shot-noise variances are

$$\sigma^2_{\mathrm{shot,tar},c} = \mu_{e,\mathrm{tar},c}, \qquad \sigma^2_{\mathrm{shot,bg},c} = \mu_{e,\mathrm{bg},c}. \tag{S.16}$$

The mean dark-current electron count during exposure time $t_c$ is

$$\mu_{\mathrm{dark},c} = i_{\mathrm{dark},c} t_c, \tag{S.17}$$

where $i_{\mathrm{dark},c}$ has units of $\mathrm{e}^-\ \mathrm{pixel}^{-1}\ \mathrm{s}^{-1}$. The dark-current shot-noise variance is equal to $\mu_{\mathrm{dark},c}$.

With all electronic noise sources referred to the sensor input, the total variance of one measurement is (*8, 9, 10*)

$$\sigma^2_{e,c} = \mu_{e,c} + \mu_{\mathrm{dark},c} + \sigma^2_{\mathrm{read},c} + \sigma^2_{\mathrm{FPN},c} + \sigma^2_{\mathrm{q},c}. \tag{S.18}$$

All terms in Eq. (S.18) have units of $\mathrm{e}^{-2}$:

- $\mu_{e,c}$ is the target-plus-background photon shot-noise variance;
- $\mu_{\mathrm{dark},c}$ is the dark-current shot-noise variance;
- $\sigma^2_{\mathrm{read},c}$ is the input-referred read-noise variance;
- $\sigma^2_{\mathrm{FPN},c}$ is the residual fixed-pattern-noise variance after calibration;

- $\sigma_{\mathrm{q},c}^2$ is the input-referred ADC quantization-noise variance.

We group the non-photon sensor-noise terms as

$$\sigma_{\mathrm{sen},c}^2 = \mu_{\mathrm{dark},c} + \sigma_{\mathrm{read},c}^2 + \sigma_{\mathrm{FPN},c}^2 + \sigma_{\mathrm{q},c}^2. \tag{S.19}$$

The total variance is then

$$\sigma_{e,c}^2 = \mu_{e,c} + \sigma_{\mathrm{sen},c}^2. \tag{S.20}$$

**Conversion gain and digital output.** The pixel readout circuit converts the collected charge into a voltage. The analog signal chain then amplifies the voltage before ADC quantization (*8*).

Let the input-referred conversion factor be

$$K_c \quad [\mathrm{e}^-/\mathrm{DN}], \tag{S.21}$$

so that $K_c$ gives the number of input electrons represented by one DN. Its reciprocal,

$$G_{\mathrm{conv},c} = \frac{1}{K_c} \quad [\mathrm{DN}/\mathrm{e}^-], \tag{S.22}$$

is the electron-to-DN conversion gain.

The digital output of one pixel is modeled as

$$D_c = D_{\mathrm{off},c} + \mathcal{Q}_{b_c}\left[\frac{X_{e,c} + n_{\mathrm{read},c} + n_{\mathrm{FPN},c}}{K_c}\right], \tag{S.23}$$

where

- $D_c$ and $D_{\mathrm{off},c}$ are expressed in DN;
- $\mathcal{Q}_{b_c}[\cdot]$ represents quantization and clipping by a $b_c$-bit ADC;
- $X_{e,c}$ is the random photoelectron and dark-electron count;
- $n_{\mathrm{read},c}$ and $n_{\mathrm{FPN},c}$ are input-referred electronic-noise terms in electrons.

Changing the analog gain changes $K_c$, and thus the number of electrons represented by one DN. It does not improve the input-referred photon-shot-noise-limited SNR because it amplifies the signal and all upstream noise together. The gain setting instead affects

- the conversion factor $K_c$;
- the ADC input range expressed in electrons;
- the input-referred quantization interval;
- the input-referred read noise;
- the digital saturation level.

**ADC bit depth and quantization interval.** For a $b_c$-bit ADC, the maximum ideal output code is

$$D_{\mathrm{FS},c} = 2^{b_c} - 1. \tag{S.24}$$

The input-referred electron range corresponding to the ADC full scale is

$$C_{\mathrm{ADC},c} = K_c D_{\mathrm{FS},c} = K_c(2^{b_c} - 1), \tag{S.25}$$

where $C_{\mathrm{ADC},c}$ has units of electrons.

Hence, one least significant bit corresponds to the input-referred electron interval

$$q_{e,c} = K_c = \frac{C_{\mathrm{ADC},c}}{2^{b_c} - 1}. \tag{S.26}$$

If the ADC electron range is approximately matched to the physical full-well capacity, this interval becomes

$$q_{e,c} \approx \frac{C_{\mathrm{full},c}}{2^{b_c} - 1}. \tag{S.27}$$

Thus, $C_{\mathrm{full}}/2^b$ is not a complete sensor sensitivity threshold. It only approximates the input-referred ADC interval when the full-well and ADC ranges are matched.

With uniform quantization, the input-referred quantization-noise variance is

$$\sigma_{\mathrm{q},c}^2 = \frac{q_{e,c}^2}{12}. \tag{S.28}$$

A conservative condition for a stable code change in one measurement is

$$\mu_{e,\mathrm{tar},c} \geq \kappa_q q_{e,c}, \tag{S.29}$$

where $\kappa_q$ is a digital-resolution factor. Setting $\kappa_q = 1$ requires the target-ballistic signal to span at least one input-referred ADC interval.

Using Eq. (S.15), this condition becomes

$$f_c \mu_{e,c} \geq \kappa_q q_{e,c}. \tag{S.30}$$

This criterion applies to a single measurement and is not a fundamental photon-detection limit. With repeated measurements and sufficient random variation, averaging may recover a signal below one LSB.

**SNR of a single measurement.** The useful signal is the mean number of photoelectrons generated by target-ballistic photons. The linear SNR of one measurement is

$$\mathrm{SNR}_{\mathrm{single},c} = \frac{\mu_{e,\mathrm{tar},c}}{\sqrt{\mu_{e,c} + \sigma_{\mathrm{sen},c}^2}}. \tag{S.31}$$

Using Eq. (S.15), this becomes

$$\mathrm{SNR}_{\mathrm{single},c} = \frac{f_c \mu_{e,c}}{\sqrt{\mu_{e,c} + \sigma_{\mathrm{sen},c}^2}}. \tag{S.32}$$

We define linear SNR as the mean target-ballistic signal divided by the noise standard deviation. In decibels,

$$\mathrm{SNR}_{\mathrm{single},c,\mathrm{dB}} = 20 \log_{10} \left( \mathrm{SNR}_{\mathrm{single},c} \right), \tag{S.33}$$

or equivalently,

$$\mathrm{SNR}_{\mathrm{single},c,\mathrm{dB}} = 10\log_{10}\left[\frac{\mu_{e,\mathrm{tar},c}^2}{\mu_{e,c}+\sigma_{\mathrm{sen},c}^2}\right]. \tag{S.34}$$

When photon-generated noise dominates the sensor-noise floor,

$$\mu_{e,c} \gg \sigma_{\mathrm{sen},c}^2, \tag{S.35}$$

Eq. (S.32) reduces to

$$\mathrm{SNR}_{\mathrm{single},c} \approx f_c\sqrt{\mu_{e,c}}. \tag{S.36}$$

The target SNR depends on both $f_c$ and the square root of the total photoelectron count. A bright image can still have a low target SNR if most photoelectrons come from path radiance.

**Multi-camera and multi-frame fusion.** Suppose that $N_{v,c}$ cameras are assigned to channel $c$ and each camera records $N_f$ frames. After registration and radiometric normalization, the number of observations is

$$M_c = N_{v,c}N_f. \tag{S.37}$$

For equal-weight averaging, the fused measurement is

$$\overline{Y}_c = \frac{1}{M_c}\sum_{i=1}^{M_c} Y_{i,c}. \tag{S.38}$$

If the observations have the same normalized target signal and noise variance, and their noise is independent, averaging preserves the mean target signal and reduces the noise variance by a factor of $M_c$. The ideal SNR gain is

$$G_{\mathrm{fus,c}} = \sqrt{N_{v,c}N_f}. \tag{S.39}$$

The fused system SNR is

$$\mathrm{SNR}_{\mathrm{sys},c} = G_{\mathrm{fus},c}\frac{f_c\mu_{e,c}}{\sqrt{\mu_{e,c}+\sigma_{\mathrm{sen},c}^2}}. \tag{S.40}$$

In the photon-noise-dominated regime,

$$\mathrm{SNR}_{\mathrm{sys},c} \approx G_{\mathrm{fus},c} f_c \sqrt{\mu_{e,c}}. \tag{S.41}$$

If the average pairwise noise correlation is $\overline{\rho}_{n,c}$, the effective fusion gain is approximately

$$G_{\mathrm{fus},c} \approx \sqrt{\frac{M_c}{1 + (M_c - 1)\overline{\rho}_{n,c}}}. \tag{S.42}$$

For $\overline{\rho}_{n,c} = 0$, Eq. (S.42) gives the ideal square-root scaling. Noise correlation, changes in the scattering medium, common illumination fluctuations, residual fixed-pattern noise, and registration errors all reduce the gain.

**Exposure time, full-well capacity, and ADC saturation.** Let $\dot{\mu}_{e,c}$ be the target-plus-background photoelectron rate in $\mathrm{e}^{-}\ \mathrm{pixel}^{-1}\,\mathrm{s}^{-1}$. The mean signal-generated photoelectron count is

$$\mu_{e,c} = t_c \dot{\mu}_{e,c}. \tag{S.43}$$

Saturation occurs when either the pixel reaches its full-well capacity or the ADC reaches its input-equivalent full scale (*11*). We define the usable charge capacity as

$$C_{\mathrm{use},c} = \eta_{\mathrm{sat}} \min\left(C_{\mathrm{full},c}, C_{\mathrm{ADC},c}\right), \tag{S.44}$$

where

- $C_{\mathrm{full},c}$ is the physical full-well capacity in electrons;
- $C_{\mathrm{ADC},c}$ is the ADC full-scale input range in electrons;
- $\eta_{\mathrm{sat}} < 1$ is a safety factor used to avoid saturation and nonlinear response.

The exposure time must satisfy

$$t_c \left(\dot{\mu}_{e,c} + i_{\mathrm{dark},c}\right) \leq C_{\mathrm{use},c}. \tag{S.45}$$

Including the frame-period and motion-blur limits, the maximum exposure time is

$$t_c^* = \min\left[\frac{C_{\text{use},c}}{\dot{\mu}_{e,c} + i_{\text{dark},c}}, t_{\text{frame}}, t_{\text{motion}}\right], \tag{S.46}$$

where $t_{\text{frame}}$ is the frame-period limit and $t_{\text{motion}}$ is the longest exposure that avoids unacceptable motion blur.

The corresponding photoelectron budget for one measurement is

$$\mu_{e,c}^{\text{use}} = t_c^* \dot{\mu}_{e,c}. \tag{S.47}$$

The maximum system SNR under these constraints is

$$\text{SNR}_{\text{sys,max},c} = G_{\text{fus},c} \frac{f_c \mu_{e,c}^{\text{use}}}{\sqrt{\mu_{e,c}^{\text{use}} + \sigma_{\text{sen},c}^2}}. \tag{S.48}$$

When photon noise dominates,

$$\text{SNR}_{\text{sys,max},c} \approx G_{\text{fus},c} f_c \sqrt{\mu_{e,c}^{\text{use}}}. \tag{S.49}$$

If the exposure reaches the usable charge limit and the dark-current contribution is small,

$$\mu_{e,c}^{\text{use}} \approx C_{\text{use},c}, \tag{S.50}$$

the maximum SNR becomes

$$\text{SNR}_{\text{sys,max},c} \approx G_{\text{fus},c} f_c \sqrt{C_{\text{use},c}}. \tag{S.51}$$

For independent observations,

$$\text{SNR}_{\text{sys,max},c} \approx f_c \sqrt{N_{v,c} N_f C_{\text{use},c}}. \tag{S.52}$$

Equation (S.52) gives a useful engineering limit. Strong scattering reduces $f_c$, the usable charge capacity limits the photoelectron count in each measurement, and multi-camera and multi-frame

fusion increases the number of independent observations.

**Role of independent channel exposure.** The red, green, and blue channels generally have different photoelectron-generation rates because the object spectrum, optical transmission, quantum efficiency, and scattering all vary with wavelength:

$$\dot{\mu}_{e,R} \neq \dot{\mu}_{e,G} \neq \dot{\mu}_{e,B}. \tag{S.53}$$

With a global exposure, all channels use the same time $t_{\text{global}}$. To prevent saturation,

$$t_{\text{global}} \leq \min_{c} \left[ \frac{C_{\text{use},c}}{\dot{\mu}_{e,c} + i_{\text{dark},c}} \right]. \tag{S.54}$$

The first channel to saturate sets the global exposure time. The other channels may then use only a small part of their available charge capacity.

With independent exposure, each channel uses its own $t_c^*$ from Eq. (S.46). For a fixed scene in the photon-noise-dominated regime, the gain over global exposure is approximately

$$G_{\exp,c} \approx \sqrt{\frac{\mu_{e,c}(t_c^*)}{\mu_{e,c}(t_{\text{global}})}} \approx \sqrt{\frac{t_c^*}{t_{\text{global}}}}. \tag{S.55}$$

Independent exposure does not change the ADC bit depth. It allows an otherwise underexposed channel to collect more target and background photoelectrons, use more of its charge capacity, and achieve a higher SNR.

**Joint digital and statistical detection conditions.** A target-ballistic signal must meet both a digital and a statistical condition.

First, the target-ballistic photoelectrons must produce a resolvable ADC code change:

$$f_c \mu_{e,c} \geq \kappa_q q_{e,c}. \tag{S.56}$$

Second, the fused SNR must exceed the required threshold:

$$\text{SNR}_{\text{sys},c} \geq \gamma, \tag{S.57}$$

where $\gamma$ is a dimensionless SNR threshold.

From Eq. (S.40), the SNR condition gives

$$f_{\mathrm{SNR},c}^{\min} = \frac{\gamma}{G_{\mathrm{fus},c}} \frac{\sqrt{\mu_{e,c} + \sigma_{\mathrm{sen},c}^2}}{\mu_{e,c}}. \tag{S.58}$$

The ADC condition similarly gives

$$f_{\mathrm{ADC},c}^{\min} = \frac{\kappa_q q_{e,c}}{\mu_{e,c}}. \tag{S.59}$$

The minimum detectable target-ballistic fraction is

$$f_{\min,c} = \max\left(f_{\mathrm{ADC},c}^{\min}, f_{\mathrm{SNR},c}^{\min}\right). \tag{S.60}$$

The target is detectable when

$$f_c \geq f_{\min,c}. \tag{S.61}$$

Thus, the target-ballistic fraction must exceed both the ADC-resolution threshold and the statistical-noise threshold.

**Optical-thickness-dependent detection limit.** For a fixed channel and optical setup, the mean target-generated photoelectron count decreases with optical thickness as

$$\mu_{e,\mathrm{tar},c}(OT_c) = \mu_{e,\mathrm{tar},c}^{(0)} \exp(-OT_c), \tag{S.62}$$

where $\mu_{e,\mathrm{tar},c}^{(0)}$ is the reference target-generated photoelectron count and $OT_c$ is the effective optical thickness of channel $c$.

The ADC-resolvability condition is

$$\mu_{e,\mathrm{tar},c}^{(0)} \exp(-OT_c) \geq \kappa_q q_{e,c}, \tag{S.63}$$

which gives the ADC-limited optical thickness

$$OT_{\mathrm{ADC},c} = \ln\left[\frac{\mu_{e,\mathrm{tar},c}^{(0)}}{\kappa_q q_{e,c}}\right]. \tag{S.64}$$

Substituting Eq. (S.26) gives

$$OT_{\mathrm{ADC},c} = \ln\left[\frac{\mu_{e,\mathrm{tar},c}^{(0)}\left(2^{b_c}-1\right)}{\kappa_q C_{\mathrm{ADC},c}}\right]. \tag{S.65}$$

A higher ADC bit depth reduces the input-referred electron interval per code, allowing a weaker target-ballistic signal to be represented.

The noise-limited optical thickness is

$$OT_{\mathrm{SNR},c} = \sup\left\{OT_c : \mathrm{SNR}_{\mathrm{sys},c}(OT_c) \geq \gamma\right\}. \tag{S.66}$$

Because the background count, target-ballistic fraction, and total photoelectron count all vary with optical thickness, $OT_{\mathrm{SNR},c}$ generally has no closed-form expression.

The detectable optical thickness is set by the more restrictive limit:

$$OT_{\det,c} = \min\left(OT_{\mathrm{ADC},c}, OT_{\mathrm{SNR},c}\right). \tag{S.67}$$

ADC resolution may limit very weak target-ballistic signals, while path radiance and sensor noise may be more restrictive in other operating conditions.

**Engineering interpretation.** The model is summarized by three quantities: the target-ballistic photoelectron fraction $f_c$, the total photoelectron count $\mu_{e,c}$, and the fusion gain $G_{\mathrm{fus},c}$.

The system SNR is

$$\boxed{\mathrm{SNR}_{\mathrm{sys},c} = G_{\mathrm{fus},c}\frac{f_c \mu_{e,c}}{\sqrt{\mu_{e,c}+\sigma_{\mathrm{sen},c}^2}}}. \tag{S.68}$$

When photon noise dominates and the exposure reaches the usable charge limit,

$$\boxed{\mathrm{SNR}_{\mathrm{sys,max},c} \approx f_c\sqrt{N_{v,c}N_f C_{\mathrm{use},c}}}. \tag{S.69}$$

Strong scattering mainly reduces the fraction of photoelectrons that carries target information. Exposure time, optical transmission, quantum efficiency, and usable charge capacity set the photoelectron count in each exposure. Multi-camera and multi-frame fusion adds independent observations. ADC bit depth and conversion gain map the collected charge to digital codes, while the final detection limit depends on both ADC resolution and the combined photon and sensor noise.

### S.1.2 Analysis of Color Space Characteristics in Strong Scattering Scenarios

In this section, we will explore the constraints in color space that occur in scattering scenarios, particularly in strong scattering conditions. In image processing, RGB and HSV color spaces are commonly used. The RGB space closely aligns with the pixel imaging mechanism of cameras, while the HSV color space better reflects human color perception, providing an intuitive representation of hue (H), saturation (S), and value (V) (*12*). Here, we will focus on the color characteristics of strongly scattered images in the HSV space. Additionally, we will discuss how current physical models regulate the color space in such scenarios.

As outlined in Sec. S.1.1, the intensity information in the three spectral bands of RGB in a strongly scattering environment is captured by the camera pixels. This data undergoes a series of photoelectric conversion processes before being transformed into a digital image. During these processes, photon shot noise becomes the dominant factor, which cannot be effectively suppressed by post-processing algorithms.

In this section, we will analyze the effect of photon shot noise on color representation in such images. To simplify our analysis and draw connections to existing de-scattering techniques, we employ the ideal atmospheric light model (ASM) (*2*), Under the assumption of uniform fog density and globally consistent lighting, this expression can be simplified in terms of S.1 as follows:

$$I_C = J_C \cdot t(C) + A_C \cdot (1 - t(C)), \tag{S.70}$$

where $I$ represents the scattered image, $J$ is the original scene, $A$ is the intensity of atmospheric light, the function $t(C) = e^{-\beta(C)u}$ denotes the scattering attenuation coefficient, and $C$ represents the color channel.

Before analyzing the properties in HSV space, we will first review how to convert RGB to

HSV space. The HSV space can be obtained from RGB by the following equations:

$$\begin{cases} C_{\max} = \max(I_R, I_G, I_B) \\ C_{\min} = \min(I_R, I_G, I_B) \ , \\ \quad \Delta = C_{\max} - C_{\min} \end{cases} \tag{S.71}$$

where $C_{\max}$ and $C_{\min}$ represent the maximum and minimum value channels in the RGB channels, respectively. The H domain in HSV space can be computed as:

$$H = \begin{cases} 0, & \Delta = 0 \\ 60 \cdot \left(\frac{I_G - I_B}{\Delta}\right), & C_{\max} = I_R \\ 60 \cdot \left(\frac{I_B - I_R}{\Delta} + 2\right), & C_{\max} = I_G \\ 60 \cdot \left(\frac{I_R - I_G}{\Delta} + 4\right), & C_{\max} = I_B \end{cases} \tag{S.72}$$

The S domain can be computed as:

$$S = \begin{cases} 0, & C_{\max} = 0 \\ 1 - \frac{C_{\min}}{C_{\max}}, & C_{\max} \neq 0 \end{cases} \tag{S.73}$$

The V domain can be computed as:

$$V = C_{\max}. \tag{S.74}$$

#### S.1.2.1 The impact of the scattering to HSV space

To simplify the analysis, we introduce several general assumptions commonly adopted in the descattering problem. For instance, we consider a scene in which the color channel intensities follow the relationship $J_R > J_G > J_B$. Under this condition, $C_{\max} = J_R$. Denoting the color channels of the image without scattering as $H_J, S_J, V_J$, the HSV color channels can be expressed in terms of

RGB as follows:

$$\begin{cases} H_J = 60 \cdot \left(\dfrac{J_G - J_B}{\Delta}\right) \\ S_J = \dfrac{J_R - J_B}{J_R} \\ V_J = J_R \end{cases}. \tag{S.75}$$

Under strong scattering conditions—commonly associated with Mie scattering (*13*)—the attenuation coefficients for the red, green, and blue channels are approximately equal, i.e., $\beta_R \approx \beta_G \approx \beta_B$. Additionally, considering the commonly applied white balance operation before preprocessing, we assume that the atmospheric light intensities are also approximately equal: $A_R \approx A_G \approx A_B$. Without loss of generality, we assume a uniform attenuation coefficient $\beta$ and a consistent atmospheric light intensity $A$ for all channels. Furthermore, we assume that the scattering process does not change the relative ordering of the color channels.

Summarizing the above assumptions, we have:

**Assumption 1.** *Assumption of the object and the fog scattering environment:*

- *The color channel of the target satisfies $J_R > J_G > J_B$.*
- *There is a function $\beta$ that satisfies $\beta = \beta_R = \beta_G = \beta_B$.*
- *There is a real number $A$ satisfying $A_R = A_G = A_B = A$.*
- *The color channel of the scattered image satisfies $I_R > I_G > I_B$.*

Therefore, we can calculate the HSV channels $H_I, S_I, V_I$ for the scattered image. Initially, $H_I$ can be computed as

$$H_I = 60 \cdot \left(\frac{J_G \cdot e^{-\beta u} - J_B \cdot e^{-\beta u}}{J_R \cdot e^{-\beta u} - J_B \cdot e^{-\beta u}}\right) = H_J. \tag{S.76}$$

Then $S_I$ can be written as:

$$S_I = \frac{J_R \cdot e^{-\beta u} - J_B \cdot e^{-\beta u}}{J_R \cdot e^{-\beta u} + A \cdot (1 - e^{-\beta u})} = \frac{J_R - J_B}{J_R + A \cdot (e^{\beta u} - 1)}. \tag{S.77}$$

Finally, $V_I$ can be computed as:

$$V_I = J_R \cdot e^{-\beta u} + A \cdot (1 - e^{-\beta u}). \tag{S.78}$$

From the above equations, we can conclude that in strong scattering scenes dominated by Mie scattering, the scattered $H_I$ generally retains the original target information $H_J$. $S_I$ gradually decreases with increasing object distance until it approaches 0, while $V_I$ gradually approaches the ambient light value $A$ as the object distance increases.

Next, we consider the contrast relationship between the target and adjacent areas in the HSV space. Here, we further assume that the hue of the adjacent area does not change and denote the object's position as $m$ and the background position as $n$. Then, we can derive that:

$$\begin{aligned} CR_{S_I} &= \left| \frac{S_I(m) - S_I(n)}{S_I(m) + S_I(n)} \right| \\ &= \left| \frac{e^{-\beta u(m)}(J_G(m) - J_B(m)) - \left(\frac{I_R(m)}{I_R(n)}\right) e^{-\beta u(n)}(J_G(n) - J_B(n))}{e^{-\beta u(m)}(J_G(m) - J_B(m)) + \left(\frac{I_R(m)}{I_R(n)}\right) e^{-\beta u(n)}(J_G(n) - J_B(n))} \right|. \end{aligned} \tag{S.79}$$

Therefore, we have $CR_{S_I} \to 1$ as $u(n) \to +\infty$. Moreover, under strong scattering conditions, the intensity of each pixel approaches the brightness of the atmospheric light. In this regime, the intensity difference between any two pixels becomes negligible, i.e., $(I_R(m) - I_R(n)) \approx 0$. Consequently,

$$\begin{aligned} CR_{V_I} &= \left| \frac{V_I(m) - V_I(n)}{V_I(m) + V_I(n)} \right| \\ &= \left| \frac{I_R(m) - I_R(n)}{I_R(m) + I_R(n)} \right| \approx 0. \end{aligned} \tag{S.80}$$

As can be seen from the above equation, if noise is not considered, the contrast in the saturation domain S does not significantly decrease but tends to approach a constant value. In contrast, the contrast in the value domain V decreases rapidly, ultimately tending towards zero as the degree of scattering increases.

Considering that a small amount of Rayleigh scattering will still be present in practical scattering scenarios, the above conclusions may be affected. Therefore, ignoring noise, the characteristics of information distribution in the HSV space are as follows:

$$\begin{cases} H_I \approx H_J \\ CR_{S_I} \to 1 \\ V_I \propto e^{-\beta u} \end{cases}. \tag{S.81}$$

It is worth noting that the above derivation assumes a wavelength-independent scattering coefficient $\beta$, i.e., $\beta_R \approx \beta_G \approx \beta_B \approx \beta$. This simplification is justified in dense fog scenarios, where scattering is dominated by Mie scattering from water droplets with diameters much larger than the visible wavelengths. Under Mie scattering conditions, the wavelength selectivity of $\beta$ is significantly weaker than in Rayleigh scattering (*14*), and the approximation of a uniform $\beta$ across visible channels has been widely adopted in the dehazing literature (*15, 16*).

#### S.1.2.2 SNR relationship of the scattered image's HSV channels

In this section, we analyze the relative magnitudes of the SNR across different channels in the HSV space intuitively. Under bright scattering conditions, photon shot noise is the dominant noise source, while Gaussian noises, such as read noise and quantization noise, are negligible. Therefore, to simplify the derivation, we ignore Gaussian noise components in our SNR calculations. Denoting the operator $\mathcal{M}$ as the approximation operation, the HSV space of the measured intensity can then be expressed as:

$$\mathcal{M}(H) = 60 \cdot (\frac{L_G - L_B}{L_R - L_B}),\ \mathcal{M}(S) = 1 - \frac{L_B}{L_R},\ \text{and}\ \mathcal{M}(V) = L_R, \tag{S.82}$$

where $L_R$, $L_G$, and $L_B$ are independent random variables that approximate $I_R$, $I_G$, and $I_B$, respectively. Additionally, since we are only considering shot noise, we assume $L_R \sim \text{Poisson}(l_R)$, $L_G \sim \text{Poisson}(l_G)$, and $L_B \sim \text{Poisson}(l_B)$. To further simplify the following derivation, we assume $2l_B > l_R > l_G > l_B > 0$.

According to the definition of the SNR, and the properties of the Poisson distribution, we have

$$SNR^2_{\mathcal{M}(V)} = \frac{\mu^2(\mathcal{M}(V))}{\sigma^2(\mathcal{M}(V))} = l_R, \tag{S.83}$$

where $\mu(X)$ denotes the expected value of $X$, and $\sigma(X)$ represents the standard deviation of $X$. To compute the SNR of other variables, we need first introduce the following properties.

Considering two random variables $X$ and $Y$, where $Y$ has support at $[0, \infty)$, then the expected

value and variance of their ratio can be approximated as (*17, 18*)

$$\mu(\frac{X}{Y}) \approx \frac{\mu(X)}{\mu(Y)}, \tag{S.84}$$

and

$$Var(\frac{X}{Y}) \approx \frac{\mu(X)^2}{\mu(Y)^2}\left[\frac{\sigma(X)^2}{\mu(X)^2} - 2\frac{Cov(X,Y)}{\mu(X)\mu(Y)} + \frac{\sigma(Y)^2}{\mu(Y)^2}\right], \tag{S.85}$$

respectively. Here, $Var$ represents the variance and $Cov$ represents the covariance.

Additionally, if two independent variables $X$ and $Y$ satisfies that $X \sim Poisson(\lambda_x)$ and $Y \sim Poisson(\lambda_y)$, then $X - Y \sim Skellam(\lambda_x, \lambda_y)$, where $Skellam$ represents the Skellam distribution. Its expected value and variance can be expressed as

$$\mu(X - Y) = \lambda_x - \lambda_y, \text{ and } Var(X - Y) = \lambda_x + \lambda_y, \tag{S.86}$$

respectively. Based on these properties, the SNR of $\mathcal{M}(S)$ can be approximated by

$$SNR^2_{\mathcal{M}(S)} \approx \frac{l_R(l_R - l_B)^2}{l_B(l_B + l_R)}. \tag{S.87}$$

Since

$$\begin{aligned}\frac{(l_R - l_B)^2}{l_B(l_B + l_R)} &< \frac{1}{2}\left(\frac{l_R - l_B}{l_B}\right)^2 \\ &< \frac{1}{2},\end{aligned} \tag{S.88}$$

then

$$SNR^2_{\mathcal{M}(S)} \lesssim SNR^2_{\mathcal{M}(V)}, \tag{S.89}$$

where $\lesssim$ represents approximately less. Similarly, the SNR of $\mathcal{M}(\Delta) = L_R - L_B$ can be approximated by

$$SNR^2_{\mathcal{M}(\Delta)} \approx \frac{(l_R - l_B)^2}{l_R + l_B}. \tag{S.90}$$

With the assumption that $l_R > l_B$, then

$$SNR^2_{\mathcal{M}(\Delta)} \lesssim SNR^2_{\mathcal{M}(S)}. \tag{S.91}$$

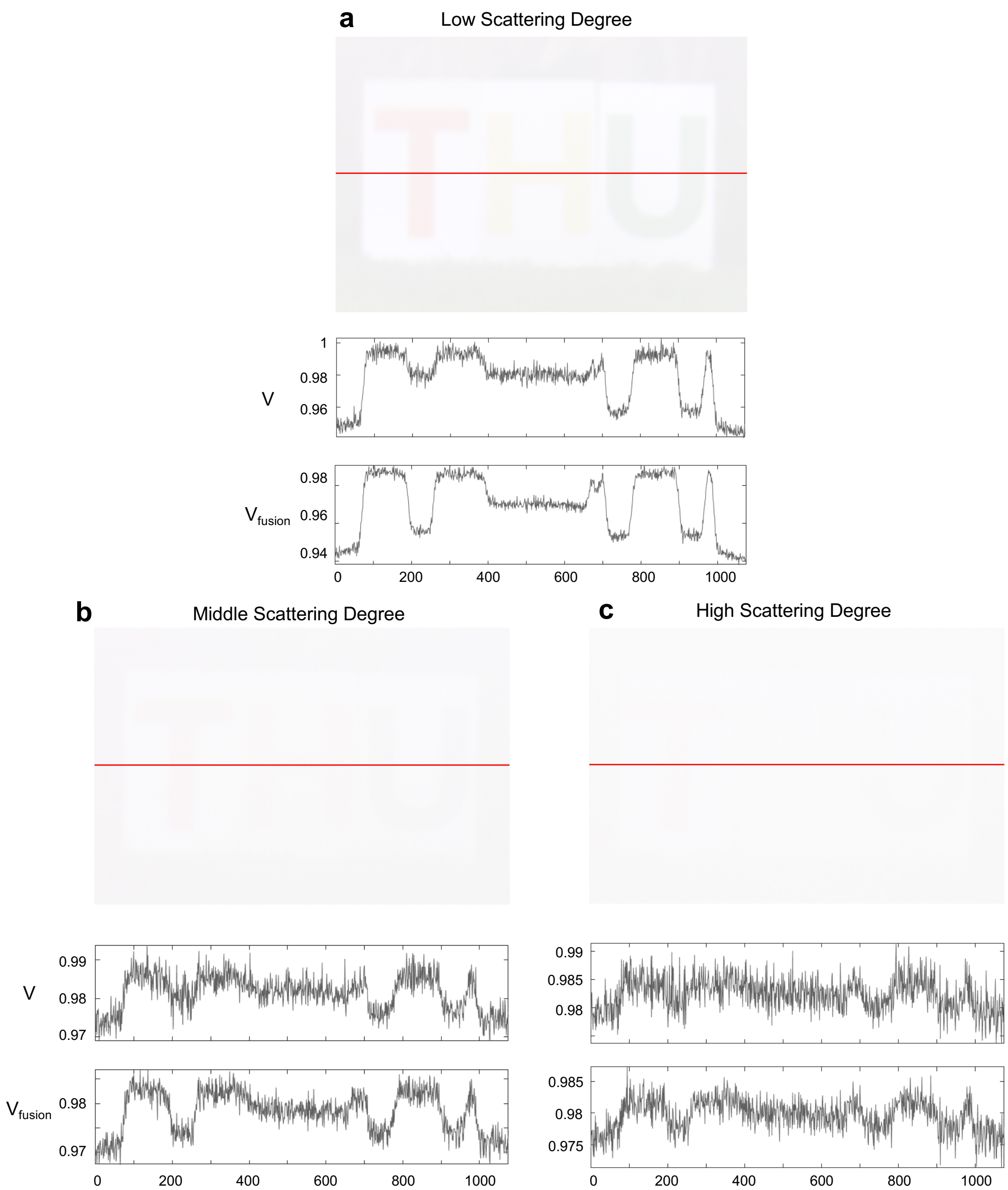


Figure S.1 **Intensity distribution of the image value domain $V$ and the fused value channel $V_{\mathbf{fusion}}$ under different scattering levels.**

Finally, the SNR of $\mathcal{M}(H)$ can be approximated as

$$SNR^2_{\mathcal{M}(H)} \approx \frac{(l_R - l_B)^2(l_G - l_B)^2}{(l_R + l_B)(l_G - l_B)^2 - 2l_B(l_R - l_B)(l_G - l_B) + (l_G + l_B)(l_R - l_B)^2}. \tag{S.92}$$

Furthermore,

$$\begin{aligned}
&\frac{(l_R - l_B)^2(l_G - l_B)^2}{(l_R + l_B)(l_G - l_B)^2 - 2l_B(l_R - l_B)(l_G - l_B) + (l_G + l_B)(l_R - l_B)^2} \\
<&\frac{(l_R - l_B)^2(l_G - l_B)^2}{(l_R + l_B)(l_G - l_B)^2 - [\sqrt{l_B}(l_R - l_B)]^2 - [\sqrt{l_B}(l_G - l_B)]^2 + (l_G + l_B)(l_R - l_B)^2} \\
=&\frac{(l_R - l_B)^2(l_G - l_B)^2}{l_G(l_R - l_B)^2 + l_R(l_G - l_B)^2} \\
<&\frac{(l_R - l_B)^2(l_G - l_B)^2}{(l_G + l_R)(l_G - l_B)^2} \\
=&\frac{(l_R - l_B)^2}{(l_R + l_G)}
\end{aligned} \tag{S.93}$$

With $l_R + l_G > l_R + l_B$, we have

$$SNR^2_{\mathcal{M}(H)} \lesssim SNR^2_{\mathcal{M}(\Delta)}. \tag{S.94}$$

Combining Eq. (S.89), (S.91) and (S.94), we have

$$SNR^2_{\mathcal{M}(H)} \lesssim SNR^2_{\mathcal{M}(\Delta)} \lesssim SNR^2_{\mathcal{M}(S)} \lesssim SNR^2_{\mathcal{M}(V)}. \tag{S.95}$$

### S.1.3 The choice of the coefficients of W

At the end of this section, we discuss how to choose the coefficients for fusing the channels to achieve the highest theoretical SNR. When capturing the image with full-well capacity, it has the following characteristics:

$$I_R = I_G = I_B. \tag{S.96}$$

Considering that there are two green channels fused before ISP processing, we can obtain the following equation:

$$2 \cdot \text{Var}^2_{I_G} = \text{Var}^2_{I_R} = \text{Var}^2_{I_B}, \tag{S.97}$$

where Var denotes the variance of the variable. We define the coefficients for the ilvalue fusion channel as:

$$\mathcal{W}(I) = xI_R + yI_G + zI_B, \tag{S.98}$$

where $x$, $y$ and $z$ are non-negative real numbers, satisfying $x + y + z = 1$. Then the SNR of the ilvalue fusion channel $V_{\text{fusion}}$ can be written as:

$$\text{SNR}^2_{\mathcal{W}(I)} = \frac{(xI_R + yI_G + zI_B)^2}{x^2\text{Var}^2_{I_R} + y^2\text{Var}^2_{I_G} + z^2\text{Var}^2_{I_B}}, \tag{S.99}$$

which can be rewritten as:

$$\text{SNR}^2_{\mathcal{W}(I)} = \frac{I_R^2}{(2x^2 + y^2 + 2z^2)\text{Var}^2_{I_R}} \tag{S.100}$$

To obtain the maximum value of the above equation, let us set $x + y = h$. The expression $2x^2 + y^2 + 2z^2$ can be transformed into:

$$2x^2 + y^2 + 2z^2 = 3\left(x - \frac{1}{3}h\right)^2 + \frac{8}{3}\left(h - \frac{3}{4}\right)^2 + \frac{1}{2}, \tag{S.101}$$

which reaches its minimum value when $h = 0.75$, $x = 0.25$, $y = 0.5$, and $z = 0.25$ under the premise that $x + y + z = 1$. Choosing this weight, the fused V channel achieves the highest SNR.

To verify the correctness of optimal coefficient selection under the independent exposure mechanism, we used real fog data for analysis. From Fig. S.1, it can be concluded that under different scattering levels, the noise fluctuation of $V_{\text{fusion}}$ is smaller than that of $V$, which is more conducive to de-scattering enhancement in strong scattering scenes.

### S.1.4 Registration and Fusion of the Fixed Camera Array

The fixed camera array consists of $N$ cameras whose relative positions and orientations remain unchanged during image acquisition. The central camera, indexed by $r$, is used as the reference camera. Registration is performed primarily using checkerboard calibration. An adaptive region-level correction is applied only when the calibration-based transformation is insufficient because the target depth is unknown or differs from the calibration plane.

#### S.1.4.1 Calibration-based registration

Before the scattering experiments, the camera array is calibrated under normal visibility using a planar checkerboard and Zhang's calibration method (*19*). All cameras record the checkerboard at several positions and orientations. For camera $i$, the projection of a three-dimensional point $\mathbf{X} = [X, Y, Z, 1]^{\mathrm{T}}$ is

$$s_i \widetilde{\mathbf{p}}_i = \mathbf{K}_i \begin{bmatrix} \mathbf{R}_i & \mathbf{t}_i \end{bmatrix} \mathbf{X}, \tag{S.102}$$

where $\widetilde{\mathbf{p}}_i = [u_i, v_i, 1]^{\mathrm{T}}$ is the homogeneous image coordinate, $\mathbf{K}_i$ is the intrinsic matrix, and $\mathbf{R}_i$ and $\mathbf{t}_i$ are the extrinsic parameters. The intrinsic, distortion, and extrinsic parameters are estimated by minimizing the checkerboard reprojection error:

$$\min_{\mathbf{K}_i, \mathbf{d}_i, \mathbf{R}_{i,q}, \mathbf{t}_{i,q}} \sum_q \sum_k \left\| \mathbf{p}_{i,q,k} - \pi\left(\mathbf{K}_i, \mathbf{d}_i, \mathbf{R}_{i,q}, \mathbf{t}_{i,q}, \mathbf{X}_{q,k}\right) \right\|_2^2, \tag{S.103}$$

where $q$ indexes the checkerboard pose, $k$ indexes a checkerboard corner, $\mathbf{d}_i$ contains the lens-distortion coefficients, and $\pi(\cdot)$ denotes camera projection including lens distortion.

All images are first undistorted using $\mathbf{K}_i$ and $\mathbf{d}_i$. The relative pose from camera $i$ to the reference camera is

$$\mathbf{T}_{i \to r} = \begin{bmatrix} \mathbf{R}_{ri} & \mathbf{t}_{ri} \\ \mathbf{0}^{\mathrm{T}} & 1 \end{bmatrix}. \tag{S.104}$$

For a target located approximately on a working plane $\Pi_i : \mathbf{n}_i^{\mathrm{T}} \mathbf{X}_i = d_i$, the mapping from camera $i$ to the reference camera is described by the plane-induced homography

$$\mathbf{H}_{i \to r}^{\mathrm{cal}} = \mathbf{K}_r \left( \mathbf{R}_{ri} + \frac{\mathbf{t}_{ri} \mathbf{n}_i^{\mathrm{T}}}{d_i} \right) \mathbf{K}_i^{-1}. \tag{S.105}$$

The sign of the translation term depends on the plane convention. Equation (S.105) follows $\mathbf{n}_i^{\mathrm{T}} \mathbf{X}_i = d_i$.

In practice, the checkerboard is placed near the expected target plane. The homography can then be estimated directly from the detected checkerboard corners:

$$\widehat{\mathbf{H}}_{i \to r}^{\mathrm{cal}} = \arg\min_{\mathbf{H}} \sum_k \left\| \mathbf{p}_{r,k} - \pi_h\left(\mathbf{H} \widetilde{\mathbf{p}}_{i,k}\right) \right\|_2^2, \tag{S.106}$$

where $\pi_h([x, y, w]^{\mathrm{T}}) = [x/w, y/w]^{\mathrm{T}}$ denotes homogeneous normalization. A robust estimator may be used to reject incorrectly detected corners. Because the camera array is mechanically fixed, the calibrated transformations are stored and reused for subsequent image sequences.

The registered image is obtained by inverse warping:

$$I^r_{i,m,c}(\mathbf{x}) = I_{i,m,c}\left[\pi_h\left(\left(\mathbf{H}^{\mathrm{cal}}_{i\to r}\right)^{-1}\widetilde{\mathbf{x}}\right)\right], \tag{S.107}$$

where $m$ is the frame index, $c \in \{R, G, B\}$ is the color channel, and interpolation is used at noninteger coordinates.

#### S.1.4.2 Adaptive registration as an optional refinement

A single calibration homography is exact only for points on the selected working plane. If the target depth differs from the checkerboard depth, residual parallax may remain after calibration-based registration. The calibration result is retained for undistortion and initial alignment, and a depth-dependent translation is then estimated from the scattering images.

The initially registered images are converted to the CIELab color space. A region of interest $\mathcal{R}$ is extracted from the Gaussian-smoothed $L^*$ channel using the Canny operator:

$$\mathcal{R} = \mathrm{ROI}\left[\mathrm{Canny}\left(G_\sigma * L^*\right)\right]. \tag{S.108}$$

The $a^*$ and $b^*$ channels are combined to preserve chromatic contrast under scattering:

$$C_i(u, v) = \left[\frac{\mathrm{Var}(b^*_i)}{\mathrm{Var}(a^*_i)} + \lambda\right]^{1/2} \left|a^*_i(u, v) - \overline{a^*_i}\right| + \left|b^*_i(u, v) - \overline{b^*_i}\right|. \tag{S.109}$$

Let $z_0$ be the checkerboard working distance. For a candidate target depth $z$, the residual disparity relative to $z_0$ is modeled as

$$\delta u_i(z) = f_x B_{x,i}\left(\frac{1}{z} - \frac{1}{z_0}\right), \qquad \delta v_i(z) = f_y B_{y,i}\left(\frac{1}{z} - \frac{1}{z_0}\right), \tag{S.110}$$

where $B_{x,i}$ and $B_{y,i}$ are the signed baseline components relative to the reference camera, and $f_x$

and $f_y$ are the focal lengths in pixel units. The residual transformation and refined homography are

$$\mathbf{S}_i(z) = \begin{bmatrix} 1 & 0 & \delta u_i(z) \\ 0 & 1 & \delta v_i(z) \\ 0 & 0 & 1 \end{bmatrix}, \qquad \mathbf{H}_{i\to r}(z) = \mathbf{S}_i(z)\mathbf{H}^{\mathrm{cal}}_{i\to r}. \tag{S.111}$$

Candidate depths are evaluated by fusing the warped chromatic feature maps. Correct alignment produces sharper target structure and a larger variance within the target region:

$$z^* = \arg\max_{z\in\mathcal{Z}} \ \mathrm{Var}_{\mathbf{x}\in\mathcal{R}} \left[ \frac{\sum_{i=1}^{N} M_i(\mathbf{x}; z)\mathcal{W}_{\mathbf{H}_{i\to r}(z)}\{C_i\}(\mathbf{x})}{\sum_{i=1}^{N} M_i(\mathbf{x}; z)} \right], \tag{S.112}$$

where $\mathcal{Z}$ is the candidate depth range and $M_i(\mathbf{x}; z)$ is a binary validity mask. If calibration alone provides sufficient alignment, this refinement is omitted and $\mathbf{S}_i = \mathbf{I}_3$.

#### S.1.4.3 Multi-frame and multi-view fusion

After registration, the final image is obtained by averaging all valid observations:

$$\widehat{I}_c(\mathbf{x}) = \frac{\displaystyle\sum_{i=1}^{N}\sum_{m=1}^{M} w_{i,m}(\mathbf{x})M_i(\mathbf{x})I^r_{i,m,c}(\mathbf{x})}{\displaystyle\sum_{i=1}^{N}\sum_{m=1}^{M} w_{i,m}(\mathbf{x})M_i(\mathbf{x})}, \tag{S.113}$$

where $M_i(\mathbf{x})$ is the validity mask and $w_{i,m}(\mathbf{x})$ is the fusion weight. Uniform weights are used when the cameras have comparable noise levels.

For a matrix form, let $\mathbf{x}_{i,m,c}$ be the vectorized input image, $\mathbf{P}_i$ the resampling matrix for undistortion and geometric warping, and $\mathbf{A}_{i,m}$ a diagonal matrix containing the validity mask and fusion weights. The fusion is

$$\widehat{\mathbf{x}}_c = \mathbf{D}^{\dagger}\sum_{i=1}^{N}\sum_{m=1}^{M} \mathbf{A}_{i,m}\mathbf{P}_i\mathbf{x}_{i,m,c}, \qquad \mathbf{D} = \sum_{i=1}^{N}\sum_{m=1}^{M} \mathbf{A}_{i,m}, \tag{S.114}$$

where $\mathbf{D}^{\dagger}$ is the pseudoinverse of the diagonal overlap matrix. This normalization prevents intensity attenuation near the boundary of the common field of view.

Calibration-based registration is used by default because it is stable and does not depend on visible scene features. Adaptive registration is used only when the target depth is unknown or differs enough from the checkerboard plane to cause noticeable residual parallax. Both methods assume a mechanically fixed camera array and negligible scene motion during multi-frame acquisition.

#### S.1.4.4 Field-of-View Fusion Error Analysis

A single calibration homography is exact only for points on the selected working plane $\Pi_i$. We now quantify the residual disparity given by Eq. (S.110) for the outdoor camera-array configuration used in the field experiments. The baseline is $B = 0.2\,\mathrm{m}$, the calibration distance is $z_0 = 700\,\mathrm{m}$, the effective focal length is $f = 1000\,\mathrm{mm}$, and the pixel pitch is $p = 6.5\,\mu\mathrm{m}$ (Table S.6), giving a focal length in pixel units of

$$f_{px} = f/p \approx 1.54 \times 10^5 \text{ pixels.} \tag{S.115}$$

Substituting these values into Eq. (S.110), a depth deviation of $\pm 10\,\mathrm{m}$ from $z_0$ produces a residual disparity of approximately $0.6$ pixels, while a deviation of $\pm 100\,\mathrm{m}$ produces a residual disparity of approximately $5.5$–$7.3$ pixels; the asymmetry reflects the nonlinear $1/z$ dependence in Eq. (S.110).

At $R = 700\,\mathrm{m}$ the object-space ground sampling distance is $\mathrm{GSD} = 4.55\,\mathrm{mm}$ per pixel (Eq. (S.144)), so the $\pm 10\,\mathrm{m}$ residual of $0.6$ pixels corresponds to a physical misalignment of approximately $2.8\,\mathrm{mm}$. This is well below the $20\,\mathrm{mm}$ smallest feature resolved after de-scattering (Sec. S.7.3, Table S.9), so the fixed calibration homography of Eq. (S.105) introduces negligible registration error over the depth range spanned by the field targets.

The fixed calibration is therefore appropriate for the quasi-planar, long-range targets used in the field experiments. This conclusion holds specifically for long-range, narrow-field-of-view scenes near the calibration depth $z_0$; scenes spanning a broad depth range would instead require either the adaptive refinement described above (Eqs. (S.108)–(S.112)) or a fully depth-aware registration scheme.

### S.1.5 Validity Analysis of previous Algorithms

In this section, we will discuss the color space adjustment mechanisms of prior-based de-scattering methods, which have been widely successful in weak scattering scenarios, as well as optimization-based de-scattering methods. These approaches generally do not change the hue domain of the scattering image but instead focus on regulation and enhancement in the S and V domains. We continue to use the ASM model as a simplified reference.

For prior-based methods, the general assumption is that the attenuation coefficient $t$ is independent of wavelength. Therefore, we can reverse the Eq.(S.70) as:

$$\begin{cases} J_{R,de} = \dfrac{I_R - A_R(1-t)}{t} \\ J_{G,de} = \dfrac{I_G - A_G(1-t)}{t} \\ J_{B,de} = \dfrac{I_B - A_B(1-t)}{t} \end{cases}. \tag{S.116}$$

Under normal circumstances, the ambient light $A$ is balanced across different bands. This is especially true for scenes with severe scattering, where performing white balance before de-scattering is necessary. Thus, with Assump. 1, the de-scattered H domain can be written as

$$H_{de} = 60 \cdot \left( \frac{J_{G,de} - J_{B,de}}{J_{R,de} - J_{B,de}} \right) = 60 \cdot \left( \frac{I_G - I_B}{I_R - I_B} \right) = H_I. \tag{S.117}$$

The S domain can be written as:

$$S_{de} = 1 - \frac{J_{B,de}}{J_{R,de}} = \frac{I_R - I_B}{I_R - A(1-t)} = \frac{I_R}{I_R - A(1-t)} \cdot S_I > S_I. \tag{S.118}$$

The V domain can be expressed as:

$$V_{de} = J_{B,de} = \frac{I_R - A(1-t)}{t} = \frac{I_R - A(1-t)}{t \cdot I_R} \cdot V_I < V_I. \tag{S.119}$$

From the above equations, we can conclude that prior-based de-scattering methods essentially do not change the H domain of the original scattered image. Instead, they enhance the S domain and lower the V domain through estimation of $A$ and $t$, making the de-scattered fog image's color

distribution more perceptible to the human eye. However, this does not solve the problem of rapid information loss in the hue domain due to a large amount of noise.

Next, we discuss the mechanism of image enhancement methods. Here, we take the widely used Retinex algorithm as an example. In conventional Retinex methods, V decomposition is performed directly, and V enhancement is achieved through convex optimization. The final enhanced RGB image is obtained by inverting the HSV image. Thus, it is easy to see that this method only regulates and enhances the V domain without changing the H and S domains.

The Retinex algorithm can also be converted to a low-light enhancement problem before inversion (*20*) , which can be written as:

$$J = 1 - \mathcal{R}(1 - I), \tag{S.120}$$

where $\mathcal{R}$ denotes the Retinex algorithm. The authors comprehensively discussed the connection between this method and prior-based methods, mainly in terms of regulation in the three RGB channels.

Here, we use the HSV space to further explore its deeper color regulation characteristics. Denote $M = \mathcal{R}(1-I)$, then the original image can be written as $J = 1 - M$. Since Retinex adjusts the image in the V domain, it does not change the relative value ratio of the three RGB channels when converted back to the RGB space. Taking a red object as an example, the relationship for converting HSV to RGB is as follows:

$$\begin{cases} I_R = V_I \\ I_G = V_I \cdot ((H_I - 1) \cdot S_I + 1) \\ I_B = V_I \cdot (1 - S_I) \end{cases} . \tag{S.121}$$

Therefore, we can simplify the Retinex-enhanced image as $M = T(1 - I)$, where $T > 1$ is the enhancement factor matrix. Therefore, we have:

$$\begin{cases} J_{R,de} = 1 - T(1 - I_R) \\ J_{G,de} = 1 - T(1 - I_G) \\ J_{B,de} = 1 - T(1 - I_B) \end{cases} . \tag{S.122}$$

The H domain of the de-scattered image can be written as:

$$H_{de} = 60 \cdot \left( \frac{J_{G,de} - J_{B,de}}{J_{R,de} - J_{B,de}} \right) = H_I. \tag{S.123}$$

While the S domain can be expressed as:

$$S_{de} = 1 - \frac{J_{B,de}}{J_{R,de}} = \frac{I_R - I_B}{I_R + (1/T - 1)} = \frac{V_I}{V_I - (1 - 1/T)} S_I. \tag{S.124}$$

The V domain can be written as:

$$V_{de} = J_{R,de} = \frac{1 - T(1 - V_I)}{V_I} V_I. \tag{S.125}$$

In the above equations, the function of $T$ is similar to that of $1/t$.

Both prior-based methods and image enhancement methods essentially modulate the S and V domains of the scattering image. However, prior-based methods rely on the relatively accurate ASM atmospheric light model, allowing for a more precise estimation of the attenuation coefficient $t$ through prior constraints. In contrast, Retinex and other image enhancement methods depend solely on optimizing the V domain and only estimate the relative relationship of an equivalent attenuation coefficient. However, they cannot obtain an accurate attenuation coefficient, resulting in slightly inferior overall color performance.

An induction of existing physically-driven de-scattering algorithms reveals that while these approaches have achieved notable success in weak scattering scenarios, they are based on the premise that Mie scattering-induced light attenuation is wavelength-independent, which allows modifications only in the S and V domains, but not in the H domain. According to Sec.S.1.2.2, which derives the SNR in the HSV domain, the H domain often becomes the first to be submerged in noise due to its extremely low SNR. Therefore, relying solely on traditional de-scattering enhancement algorithms does not yield effective results in strong scattering environments.

## S.2 PCDP framework

This section will discuss the effectiveness of the proposed PCDP de-scattering framework. Stability and effectiveness are among the most critical considerations for the instrument system's task of target recovery in strong scattering environments.

**Analysis of Scattering Influence Factors:** As analyzed in the preceding sections, dominant speckle noise in strong scattering scenes leads to severe information loss in the color space. Targeted optimization based on existing algorithms can significantly enhance the instrument's de-scattering performance.

The ASM model can be compromised in strong scattering scenarios due to multiple factors. Before light enters the imaging system, the light that the system is set to receive primarily consists of target ballistic light $I_{\text{tar}}$ and ambient light $I_{cir}$:

$$I = I_{\text{tar}} + I_{cir}. \tag{S.126}$$

Existing de-scattering approaches generally assume that the ambient light $I_{cir}$ is constant, which is reasonable when scattering is weak. In such cases, the scattering coefficient is small, and the attenuation of light due to scattering within the tangential range of the image is limited. However, this assumption breaks down in strong scattering scenarios. The sun does not guarantee uniform illumination of the earth, generally presenting a certain angle with the ground. In strong scattering, the tangential scattering of the scene cannot be ignored, resulting in overall non-uniform ambient light value. Furthermore, in addition to Mie scattering, Rayleigh scattering also occurs during light transmission, due to the presence of small water particles and other dust particles in the air. Therefore, the air exhibits selective scattering for different wavelengths, causing the scattered image to display a gradient effect in the overall background color, significantly affecting the overall color perception of the image.

Furthermore, considering the light entering the optical system, it is known from the imaging formula Eq. (S.4) that light of the same intensity, upon entering the optical system, will experience energy attenuation following a $(\cos\theta)^4$ law due to off-axis deviations. This is also a common vignetting effect observed in camera lenses, leading to non-uniform distribution of light energy. Minute dust particles on the lens surface can also cause extremely subtle changes in the optical

transmission system. Under weak scattering conditions, these effects are not significant and do not affect the dominant factors in de-scattering. However, under strong scattering conditions, where the ballistic light signal is minimal, any slight perturbation in energy can lead to substantial deviations in the recovery of target information. Therefore, eliminating the aforementioned impacts is a non-negligible issue in strong scattering environments.

A pseudocode of the proposed PCDP framework is provided in Alg. S.1. Our framework is divided into the following parts: light field comprehensive correction, Denoising of H and S domains, Enhancement of S and V domains, and V adjustment of the scene. These steps effectively solve the challenge of recovering target information in common strong scattering conditions.We assume that the original scattered image received by the scene is denoted by $I$:

**Scattering-aware illumination compensation** Lens and Dust Correction: After the imaging system has been calibrated, the orientation of the device is adjusted in fog to face the distance or a whiteboard, ensuring that the instrument system only receives information on light distribution. This completes the collection of the bright field image $P_{\text{sys}}$ for the system's integrated field of view. The corrected image is given by $I_{\text{com}} = \frac{I}{P_{\text{sys}}} \cdot \text{mean}(I)$. This operation effectively compensates for lens vignetting and the impact of lens dust on imaging. For outdoor scene observation, this step is indispensable, as image disruption caused by dust on the lens is very difficult to correct in post-processing algorithms. Illumination compensation: We use Legendre orthogonal polynomials for global illumination estimation. The commonly used multiscale average filtering for illumination estimation cannot exclude target information, thereby hindering accurate prediction of the complex global light field. By using Legendre orthogonal polynomials and adjusting the order of fitting, we can effectively exclude object information and then make a precise estimate of the global illumination, $I_{\text{est}}$. This is particularly important when dealing with outdoor lighting and changes in the concentration of scattering media. The compensated image is given by $I_{\text{com}} = \frac{I_{\text{com}}}{I_{\text{est}}}$.

**HSV decoupled processing:** After the above steps, the non-uniform background information in the image has been largely corrected. We have discussed the characteristics of the color space and its SNR. In strong scattering scenarios, the hue domain $H$ generally retains the original target information but has the lowest SNR. Existing de-scattering methods do not restore the $H$ domain; hence, to recover the original color information of the $H$ domain, we use edge-preserving filtering guided by the fused V domain to denoise the image, effectively reducing the noise in

all color channels, especially in the $H$ domain as well as $S$ domain: $I_{\text{de}} = D_{\Omega}(I_{\text{com}}, V_{I_{com}}^{fusion})$. In our approach, we use $V_{\text{fusion}}$ as a guide image, calculated as the weighted average of the RGB channels, for de-scattering enhancement, as it has the highest SNR. Finally, to eliminate color casts we use white balance operations to correct the colors of the image, $I_{\text{de}} = \text{WB}(I_{\text{de}})$, the algorithm WB performs white balance by shifting the fog's signal cluster in HS space back to the origin. By discretizing polar HS coordinates into a mapping $sp(sx, sy)$, it compensates for the cluster's deviation via a global translation, restoring a centered color distribution for accurate reconstruction.

**Adaptive value reconstruction:** when the noise has been sufficiently suppressed, we consider enhancing the saturation and value domains of the image, which is particularly important for the human eye to recognize de-scattered target information. In strong scattering scenarios, many prior-based schemes often fail due to extremely low SNR. Here, we use a de-scattering enhancement scheme based on low-light enhancement inversion. The color is finally adjusted by $L$, where a larger $L$ results in more thorough de-scattering, and vice versa, $I_{\text{out}} = \frac{1-I_D}{L}$.The above de-scattering enhancement operations can be represented in the HSV domain as follows:

$$\begin{cases} H_J = H_I \\ S_J = \dfrac{V_I}{V_I - (1 - \frac{L}{T})} S_I \\ V_J = 1 - \dfrac{T}{L}(1 - V_I) \end{cases} . \tag{S.127}$$

Further denoising is then performed using median filtering $M_{\Omega}$ and guided edge-preserving filtering $D_{\Omega}$. In complex outdoor environments, to reduce computation time and enhance algorithm stability, it is advisable not to use complex denoising operations.

**V adjustment:** Since the aforementioned operations do not strictly rely on the ASM model but are based on Retinex-optimized enhancement, the overall image undergoes a value adjustment to achieve 75% of full value. This adjustment results in the best perceptual quality of the de-scattered image.The complete PCDP de-scattering algorithm process is as follows:

**Algorithm S.1** Pseudocode of the PCDP

**Require:** $I$, $P_{sys}$, $w$, $lel$, $M$, $N$
**Ensure:** $Res$

**Scattering-aware illumination compensation:**
1. $I_{com} = I/P_{sys} \cdot \text{mean}(I) \leftarrow$ lens and dust correction
2. $I_{est} = \sum_{n=0}^{N} \sum_{m=0}^{M} c_{nm} P_n(x') P_m(y')$
3. $I_{com} = I_{com}/I_{est} \leftarrow$ illumination compensation

**HSV decoupled processing:**
4. $V_I^{fusion} = 0.25I^R + 0.5I^G + 0.25I^B$
5. $I_{de} = D_\Omega(I_{com}, V_{I_{com}}^{fusion})$
6. $I_{de} = WB(I_{de})$

**Adaptive value reconstruction:**
7. $I_D = 1 - I_{de} \leftarrow$ convert to dark image
8. $V' = \text{Retinex}(V_{I_D}^{fusion})$
9. $V(I_D) = V'$
10. $I_{out} = (1 - I_D)/L$
11. $I_{out} = M_\Omega(I_{out})$
12. $I_{out} = D_\Omega(I_{out})$

**V adjustment**
13. $Res = lel/\text{mean}(I_{out}) \cdot I_{out}$

**Computational complexity analysis:** Let the input image resolution be $H \times W$, with $P = HW$ pixels. The main computational stages of PCDP are summarized in Table S.1. Per-pixel corrections and fixed-window filters scale linearly with the number of pixels. The global illumination model uses a fixed-order two-dimensional Legendre basis, while airlight estimation and the frequency-domain HLRP solver contain the dominant sorting and Fourier-transform operations.

Table S.1 Computational complexity of the main stages in the PCDP framework.

| **Stage** | **Time complexity** |
|---|---|
| Per-pixel operations and fixed-window filtering, including guided and median filters | $O(P)$ |
| Legendre-polynomial illumination fitting | $O\big(CP(N+1)^2\big)$, where $C = 3$ and $N \leq 5$ |
| Airlight estimation by sorting the dark-channel pixels | $O(P \log P)$ |
| HLRP optimization using frequency-domain FFT/IFFT operations | $O(P \log P)$ |

The number of color channels $C$, polynomial order $d$, filter-window sizes, and HLRP iterations are fixed and do not increase with image size. The overall time and memory complexities therefore

reduce to

$$T(H,W) = O(HW\log(HW))\,, \qquad S(H,W) = O(HW). \tag{S.128}$$

For the $715 \times 1075$ cropped images used in this work, $P = 768{,}625 \approx 7.69 \times 10^5$. The seven core FFT/IFFT operations in the HLRP module correspond to approximately $7P\log_2 P \approx 1.05 \times 10^8$ butterfly operations. This value describes the implementation scale of the frequency-domain computation rather than the measured wall-clock time.

# S.3 Imaging Model Validation

This section experimentally validates the sensor-noise, SNR, frame-fusion, and bit-depth predictions of the imaging model developed in Sec. S.1.1. The analysis is performed in the digital domain and uses two independently acquired fog-scene data sets for calibration and held-out validation.

## S.3.1 Sensor-Domain Model and Validation Quantities

The optical signal is first converted into accumulated photoelectrons and is then mapped to digital counts. Consistent with the radiometric model in Sec. S.1.1, a compact sensor-domain description is

$$\mu_e = t_{\exp} A_{\text{pix}} \int E_\lambda QE(\lambda) \frac{\lambda}{hc_0}\, \mathrm{d}\lambda, \qquad D = \frac{\mu_e}{K_c}, \tag{S.129}$$

where $E_\lambda$ is the spectral irradiance at the sensor, $t_{\exp}$ is the exposure time, $A_{\text{pix}}$ is the photosensitive pixel area, $QE(\lambda)$ is the quantum efficiency, and $K_c$ is the input-referred conversion factor in electrons per DN. The offset-subtracted mean signal $\bar{D}$ is therefore expressed in DN.

All experimental comparisons below are performed in this digital domain. The measured temporal variance is represented by the photon-transfer model

$$\sigma_D^2 = b + a\bar{D} + c\bar{D}^2, \qquad a = \frac{1}{K_c}, \tag{S.130}$$

where $b$ is the signal-independent variance, including read-noise, dark-current, and other electronic contributions; $a\bar{D}$ is the shot-noise variance; and $c\bar{D}^2$ represents signal-proportional multiplicative

noise. Quantization noise is included through its variance, approximately $q^2/12$ for an ideal quantizer with step $q$. Thus, bit depth affects the result through the quantization step and the measured noise floor rather than defining the detection threshold by itself.

The reported SNR is an amplitude ratio,

$$\mathrm{SNR} = \frac{\left|\bar{D}_t - \bar{D}_b\right|}{\sigma_b}, \qquad \mathrm{SNR}_{\mathrm{dB}} = 20\log_{10}(\mathrm{SNR}), \tag{S.131}$$

where $\bar{D}_t - \bar{D}_b$ is the target–background contrast and $\sigma_b$ is the measured temporal standard deviation of the background.

### S.3.2 Experimental Data Description

The experimental data used throughout this section were collected at the outdoor fog site described in the supplementary materials. Two fog-scene sessions were conducted at the same location on different days and are denoted Scene 1 and Scene 2. Although the imaging site and camera configuration remained unchanged between sessions, the ambient conditions differed, yielding two statistically independent scene ensembles.

Within each scene session, distinct fog-density levels were established by adjusting the fog generator and allowing the chamber to reach a quasi-steady state before acquisition. Each steady-state condition defines one group: Scene 1 comprises Groups 1–6, and Scene 2 comprises Groups A–C. The groups correspond to different fog concentrations and span scattering conditions from moderate to strong.

For every group, the camera array acquired 10 consecutive frames within a one-second interval at the stabilized fog density. Because the fog concentration remained effectively constant over this short acquisition window, the 10 frames within each group were treated as independent, identically distributed samples of the same scattering condition. This design enabled both single-frame noise characterization and multi-frame fusion analysis using a common data set.

In the following analyses, Scene 1 (Groups 1–6) is used for model calibration, whereas Scene 2 (Groups A–C) serves as a held-out validation set for evaluating cross-scene generalization.

### S.3.3 Cross-Scene Calibration and Intrinsic Shot-Noise Fraction

Equation (S.130) was calibrated using the six Scene 1 groups and tested directly on the independent Scene 2 groups. The fitted conversion factor was $K_c = 0.1062\ \mathrm{e}^-/\mathrm{DN}$. The held-out variance biases were $0.99\%$, $3.80\%$, and $4.10\%$ for Groups A–C, respectively, corresponding to a mean absolute bias of $2.96\%$.

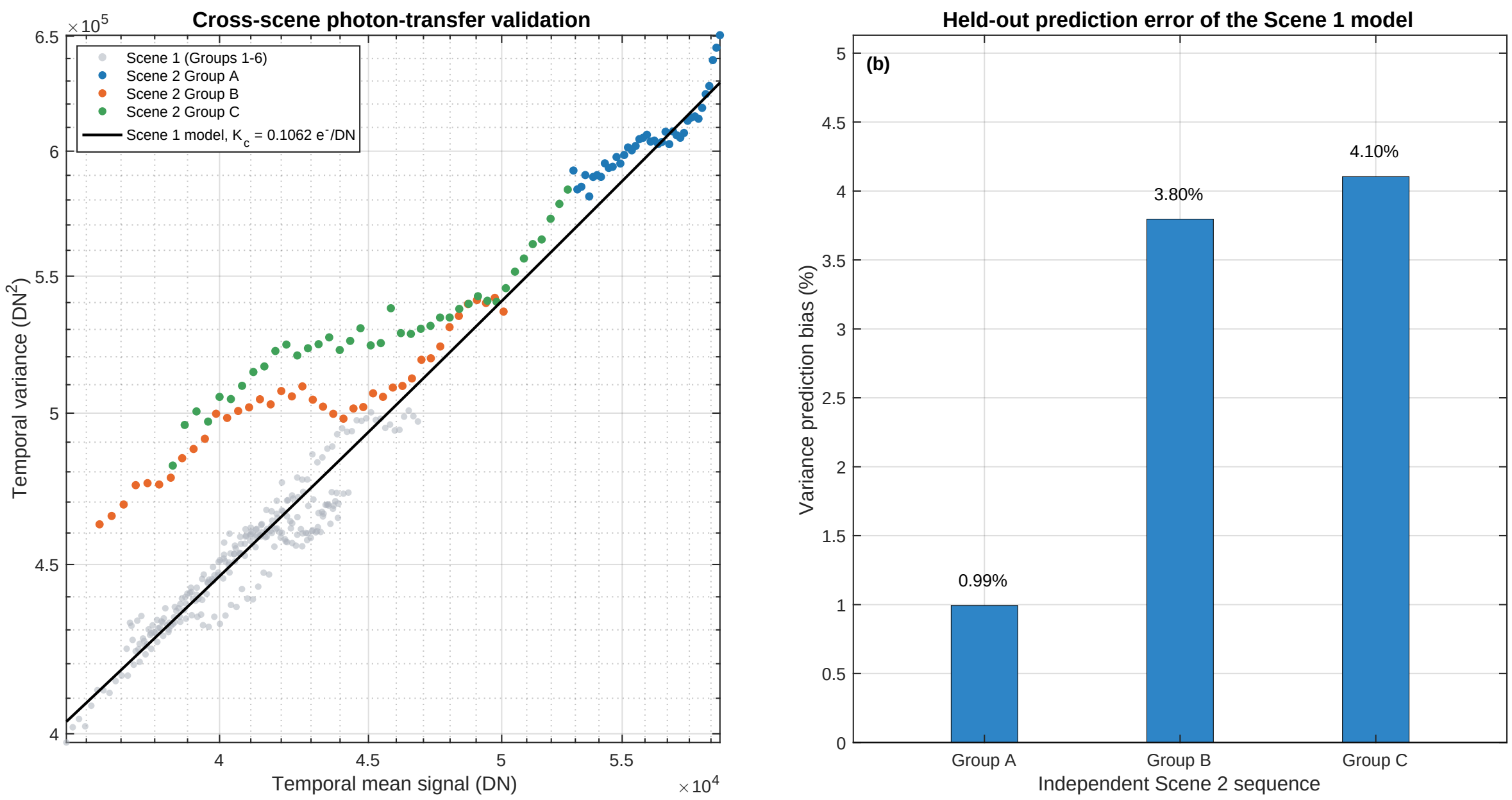


Figure S.2 **Cross-scene photon-transfer validation.** Scene 1 provides the calibration groups, whereas Scene 2 is used only for held-out prediction.

For the fitted cross-scene model, $c = 0$ and $b = 69688.98\ \mathrm{DN}^2$. The intrinsic shot-noise fraction is therefore calculated directly from the variance decomposition:

$$f_{\mathrm{shot}}(\bar{D}) = \frac{a\bar{D}}{b + a\bar{D} + c\bar{D}^2} = \frac{a\bar{D}}{b + a\bar{D}}. \tag{S.132}$$

The resulting fractions are summarized in Table S.2. Shot noise is the dominant intrinsic temporal-noise term at the tested operating points, although the nonzero signal-independent floor remains measurable.

Table S.2 **Intrinsic shot-noise fractions for the calibration and held-out data sets.**

| Data set | Mean-signal range (DN) | Intrinsic shot-noise fraction |
|---|---|---|
| Scene 1, Groups 1–6 | $38479.50–44112.41$ | $83.87\%–85.63\%$ |
| Scene 2, Groups A–C | $44882.45–56145.59$ | $85.85\%–88.35\%$ |

## S.3.4 Direct Validation of Noise and SNR

The independently calibrated noise model reproduces the measured temporal background noise for all six calibration groups. The variance prediction errors range from $-4.76\%$ to $+5.37\%$, without a systematic increase across the tested scattering conditions.

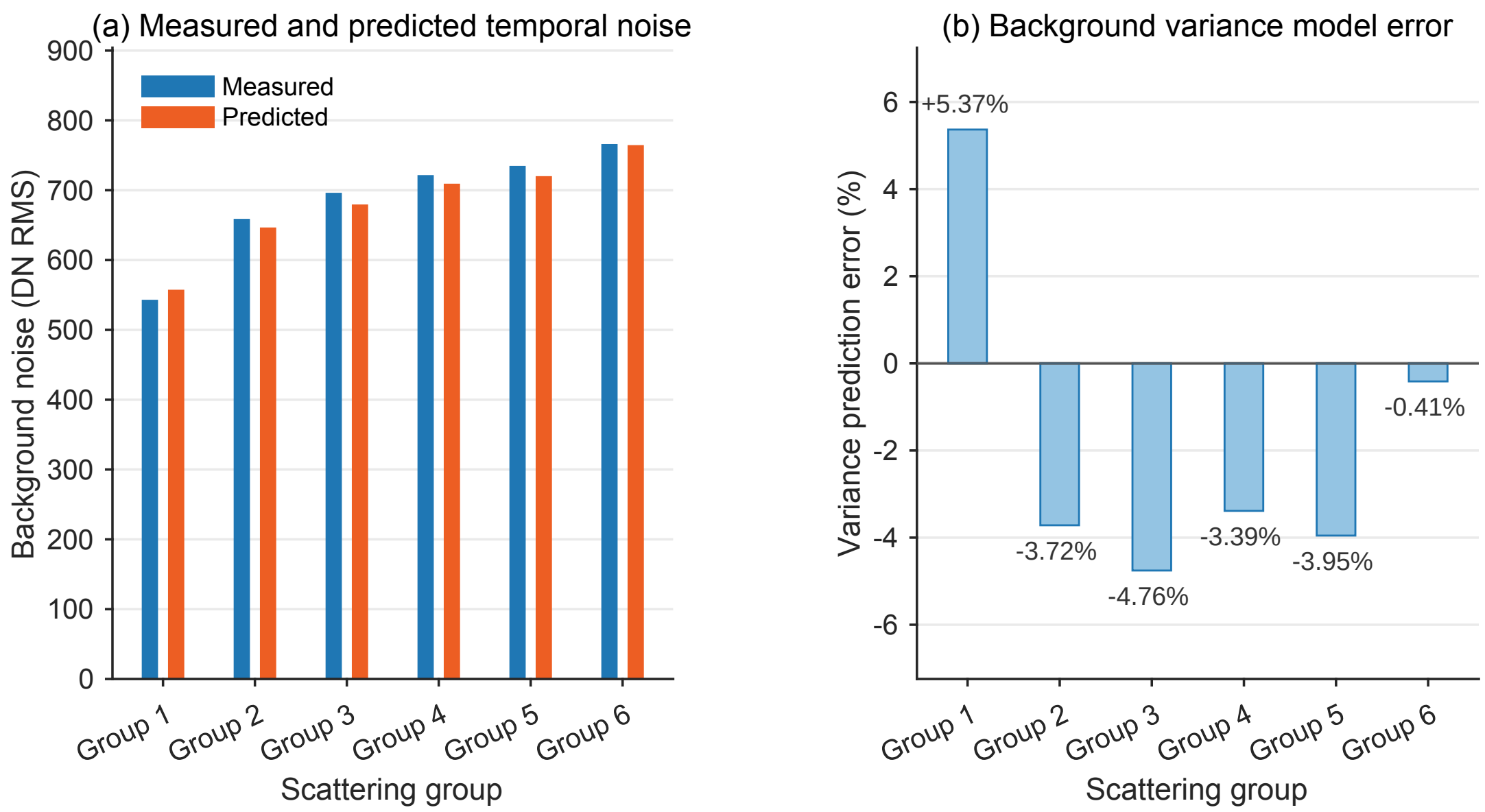


Figure S.3 **Validation of the temporal background-noise model.** The left panel compares the measured and predicted noise, and the right panel reports the variance error for each group.

Using the same measured target–background contrast and the background noise predicted by Eq. (S.130), the predicted SNR closely follows the measured SNR. The six-group comparison gives a mean absolute percentage error of $1.82\%$ and $R_0^2 = 0.9995$ for a zero-intercept agreement fit.

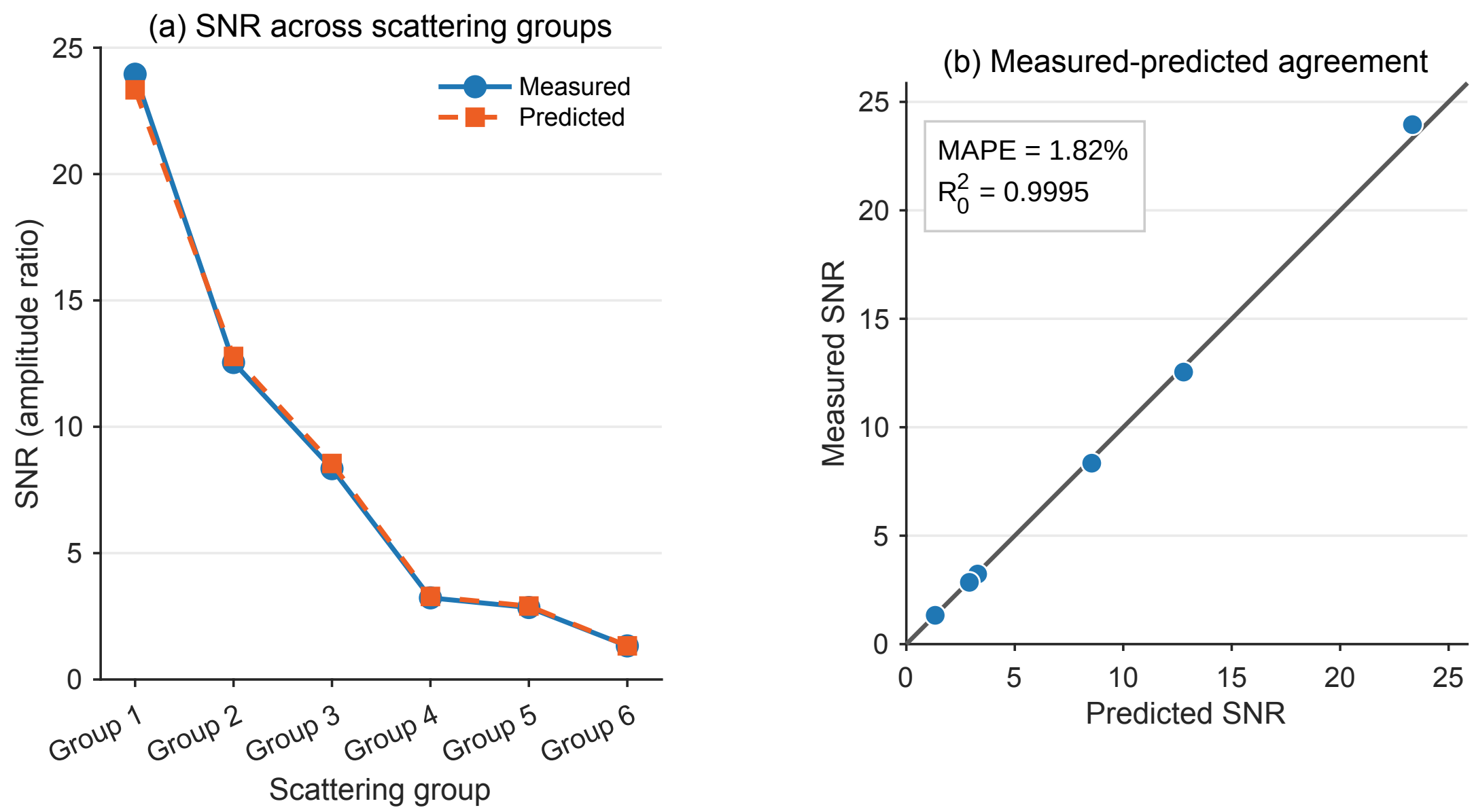


Figure S.4 **Measured and model-predicted SNR under six scattering conditions.** The predictions use the measured target–background contrast and the independently calibrated background-noise model.

### S.3.5 Validation of Frame-Number and Bit-Depth Scaling

For the average of $N_f$ statistically independent frames, the model predicts $\sigma_{N_f} = \sigma_1/\sqrt{N_f}$ and hence an SNR gain of $\sqrt{N_f}$. Measurements using one to five frames agree with this scaling: the deviations from the ideal gain remain between $-1.09\%$ and $+1.17\%$, with an overall mean absolute percentage error of $0.40\%$.

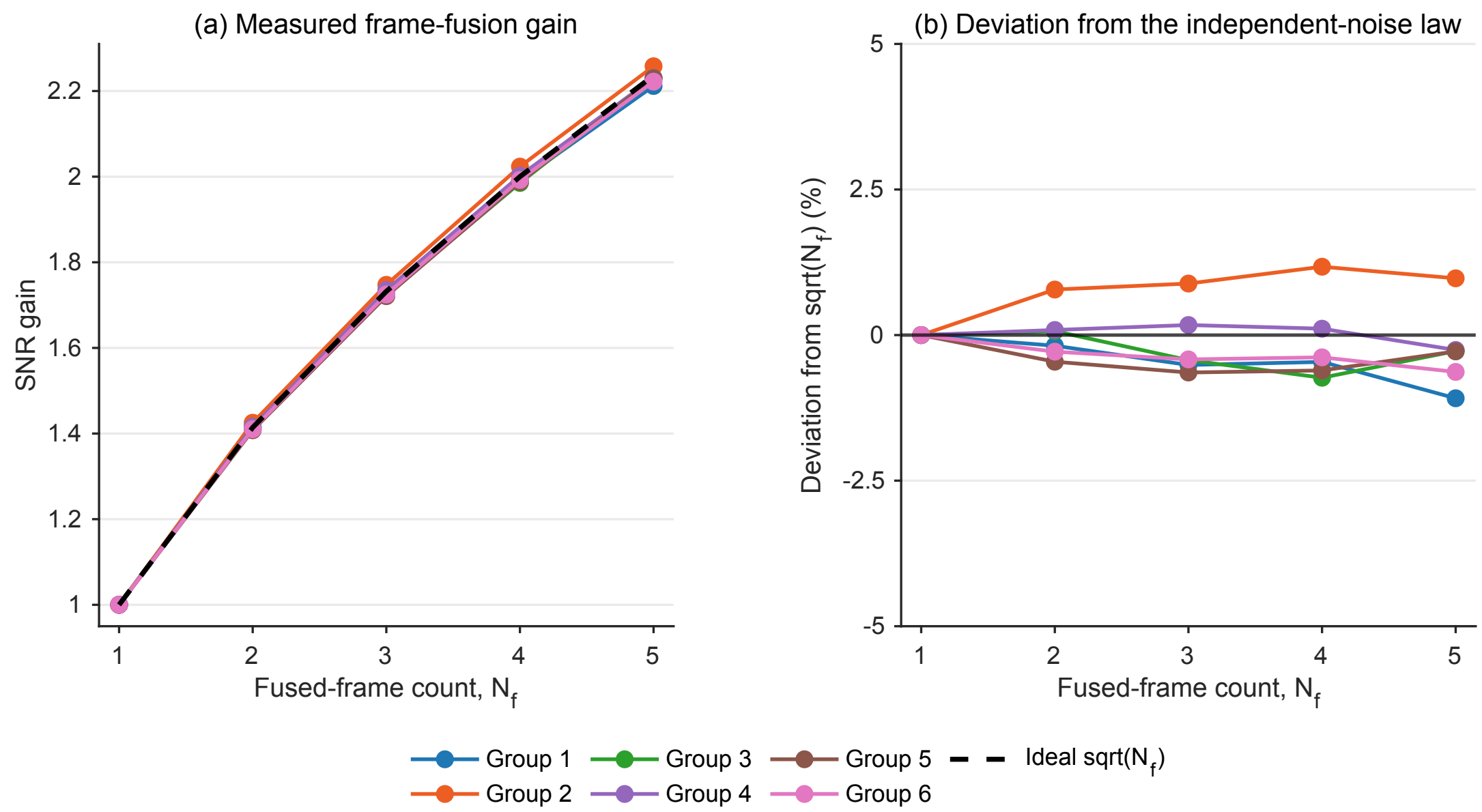


Figure S.5 **Validation of the frame-fusion gain.** The measured gain is compared with the ideal $\sqrt{N_f}$ prediction.

The bit-depth experiment separates the predicted and measured SNR from the corresponding change in the background-noise floor. From 16 to 12 bit, the SNR prediction has an overall mean absolute percentage error of $1.23\%$. The median measured SNR retention is $98.86\%$ at 12 bit, $91.75\%$ at 11 bit, and $77.62\%$ at 10 bit, showing the onset of quantization-limited degradation at the lowest settings.

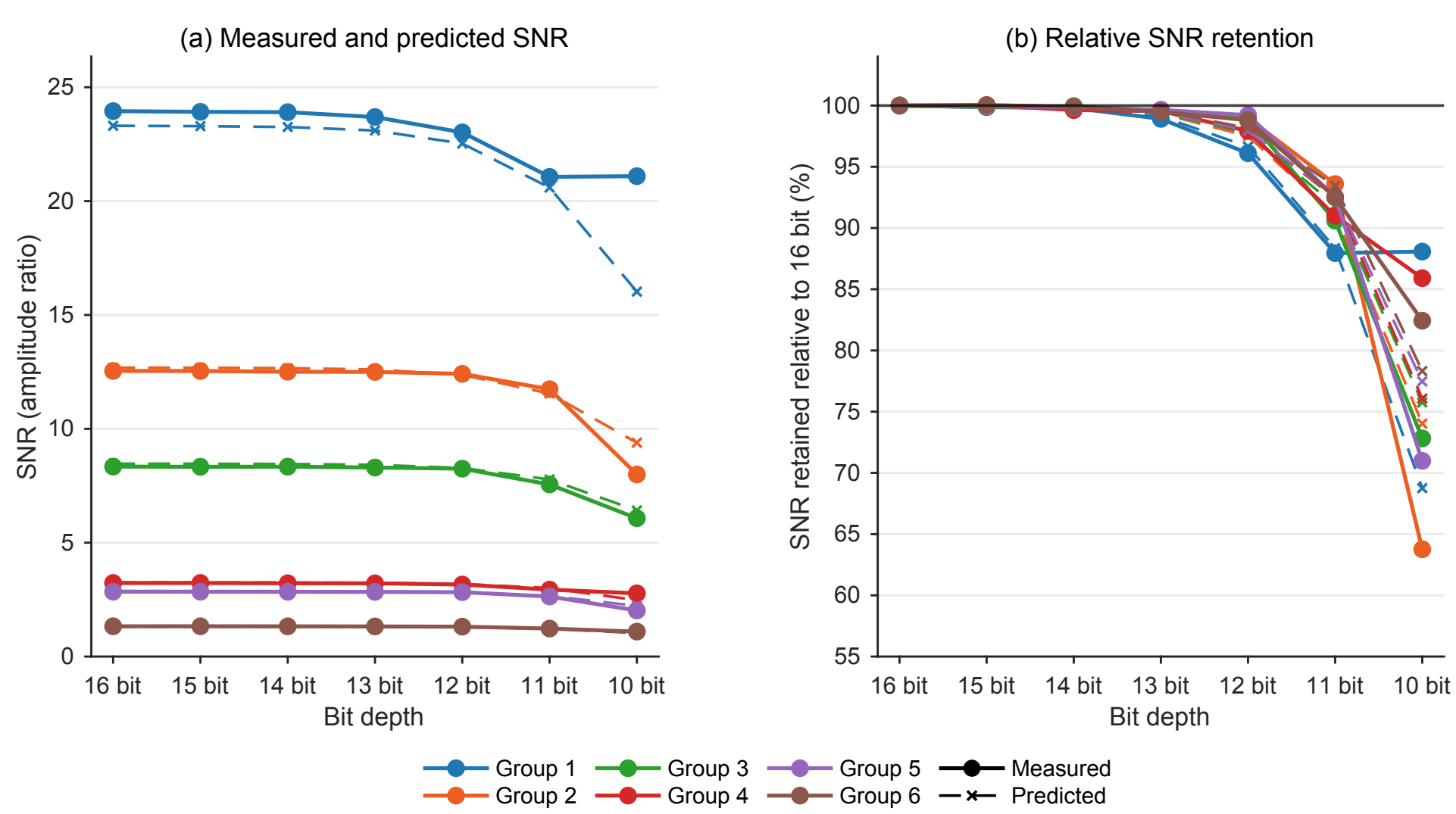


Figure S.6 **Measured and predicted SNR as a function of effective bit depth.**

Consistently, the median measured background-noise level remains close to the 16-bit reference

down to 12 bit ($101.13\%$), but increases to $109.02\%$ at 11 bit and $128.46\%$ at 10 bit. The predicted values of $102.21\%$, $108.54\%$, and $130.84\%$, respectively, reproduce this transition.

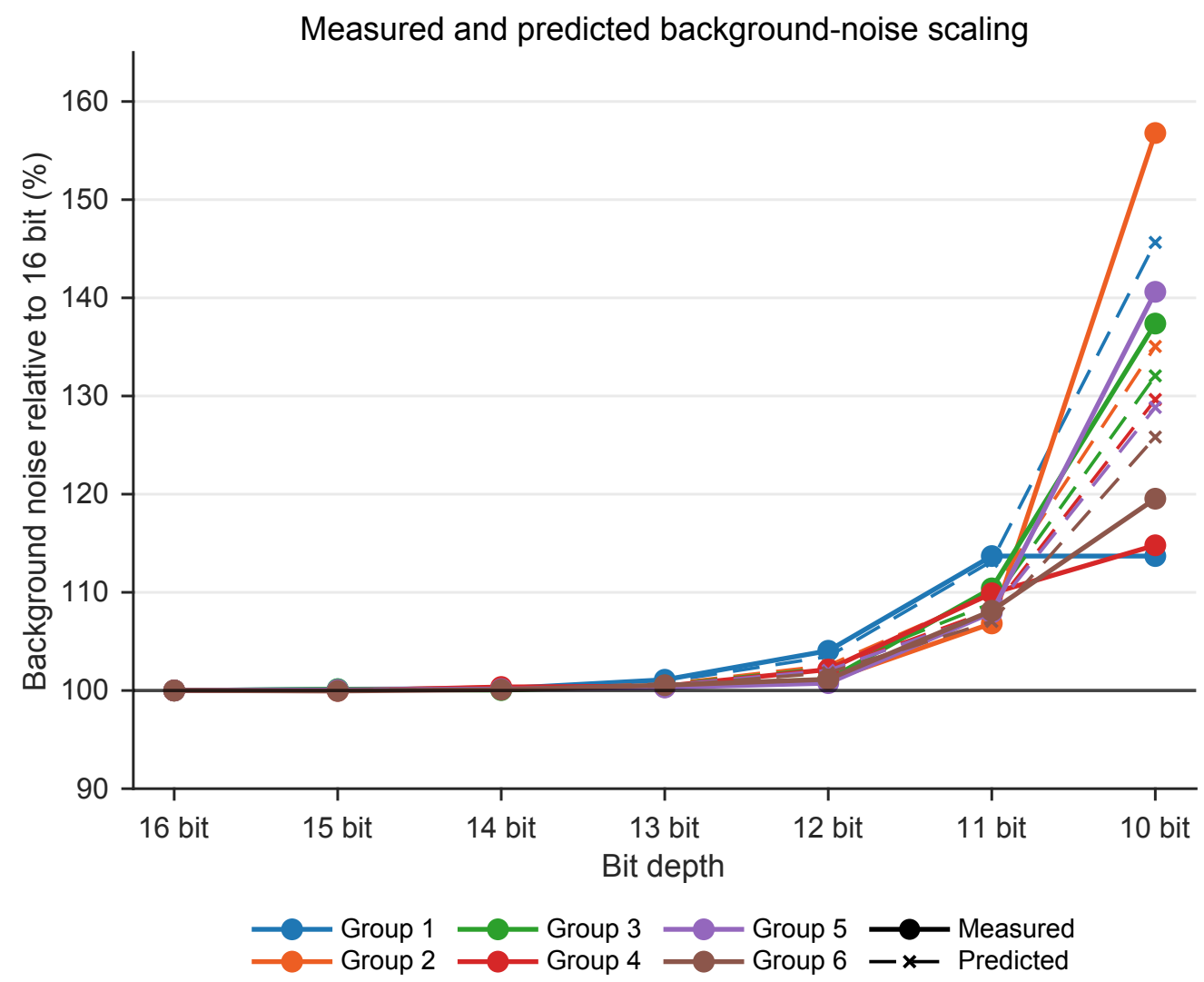


Figure S.7 **Measured and predicted background-noise scaling with effective bit depth.**

### S.3.6 Forward-Scattered Light Assumption Analysis

For volume-scattering media such as fog, forward-scattered light typically constitutes a small fraction of the received signal and has a limited effect on image degradation; it is therefore commonly neglected at the algorithmic level. This is one of the central assumptions underlying dehazing and de-scattering reconstruction, and it has been validated in numerous prior works (*15,20*),. Rather than seeking an independent quantitative proof, this section reports a qualitative check of this assumption on our own fog data, using a point-spread-function (PSF) analysis of the target's reflected light as a complementary, illustrative line of evidence alongside the established literature. Building on this PSF analysis, we further examine whether its qualitative trend is consistent with the proposed signal-to-noise ratio (SNR) model for dense-fog imaging.

The basis for using the shape of the target-reflected-light PSF to determine the presence of forward-scattered light is the atmospheric point spread function (APSF) theory developed for actively illuminated targets. When forward-scattered light is present in the system, high-frequency information is lost and the PSF broadens spatially; conversely, in the absence of forward-scattered light, the PSF remains approximately a spatial delta function.

Under passive illumination in a fog environment, modeling the PSF of the target's reflected

light first requires removing the interference of ambient light. To simplify the analysis and eliminate confounding factors, the measurements reported in this section were again performed in the fog chamber. Differencing between the color channels of the camera's raw-format image effectively cancels the interference of the ambient-light term $I_s(\mathbf{x})$ (the path-radiance/ambient contribution introduced in Sec. S.1.1); to preserve channel independence in this differencing operation, the raw-format image used here is the un-demosaiced Bayer raw output, and the sub-pixel spatial offset of at most 1 pixel introduced by the Bayer color-filter layout is neglected. Denoting the channel pair as $c_1 \in \{R, G, B\}$ and $c_2 \in \{G, B, R\}$, the channel-differenced raw image is given by

$$I^{c_1}(\mathbf{x}) - I^{c_2}(\mathbf{x}) = k_{\text{cam}}\pi^2 e^{-\beta u(\mathbf{x})} \left(J^{c_1}(\mathbf{x}) - J^{c_2}(\mathbf{x})\right) + n^{c_1}(\mathbf{x}) - n^{c_2}(\mathbf{x}), \tag{S.133}$$

where $I^c(\mathbf{x})$ and $J^c(\mathbf{x})$ denote the raw pixel value of channel $c$ at pixel $\mathbf{x}$ in the scattered and clear (fog-free) images, respectively; $k_{\text{cam}}$ is a channel-independent radiometric conversion constant of the imaging system (combining aperture, lens transmission, and sensor responsivity), which, together with the illumination and lens-vignetting nonuniformity, cancels in the channel-difference operation because it is common to both channels; $\beta$ is the attenuation coefficient, approximately wavelength-independent under the Mie-scattering condition discussed in Sec. S.1.1, and $u(\mathbf{x})$ is the propagation distance; and $n^c(\mathbf{x})$ is the pixel-wise noise realization in channel $c$, comprising the photon shot noise together with the residual sensor-noise terms characterized in Sec. S.1.1.

After removing the ambient-light interference in this way, the PSF response relating the channel difference of the original fog-free target to that of the same target under fog can be estimated. If no forward-scattered light is present, this PSF response consists of a delta function superimposed on noise; if forward-scattered light is present, the delta-function component of the PSF broadens spatially as the fog density increases. The experimental results are shown in Fig. S.8. Fig. S.8**a** shows the channel difference of the original fog-free target computed from the fog-free camera image, $k_{\text{cam}}\pi^2 \left(J^{c_1}(\mathbf{x}) - J^{c_2}(\mathbf{x})\right)$, using a green flat lettering target "T" as the reflective target, with $c_1 = G$ and $c_2 = R$. Fig. S.8**b–f** show, for the same target, the channel difference of the fog image $I^{c_1}(\mathbf{x}) - I^{c_2}(\mathbf{x})$ at increasing optical thickness ($OT = 1.5$ to $OT = 6$), together with the one-dimensional cross-section of the resulting two-dimensional reflected-light PSF taken through the peak location, as indicated by the red marker in Fig. S.8**b**. The two-dimensional PSF is obtained

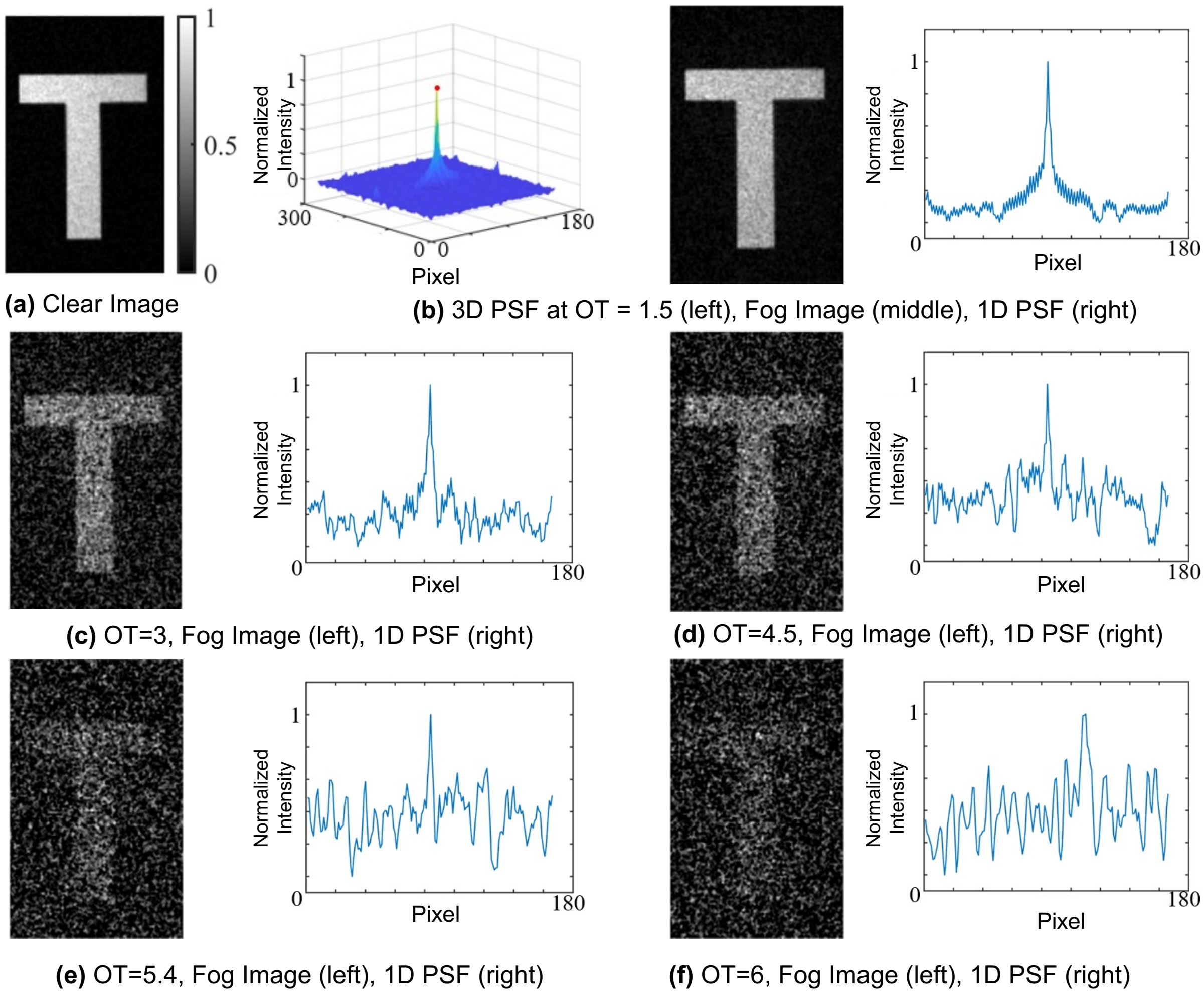


Figure S.8 **Point-spread-function analysis of the target-reflected-light channel difference under increasing fog density. a** Channel difference $k_{\text{cam}}\pi^2(J^G(\mathbf{x}) - J^R(\mathbf{x}))$ of the fog-free reference target. **b** Three-dimensional PSF at $OT = 1.5$ (left), the corresponding fog image (middle), and its one-dimensional PSF cross-section (right); the red marker indicates the peak location used for the cross-section in all panels. **c–f** Fog image (left) and one-dimensional PSF cross-section (right) at $OT = 3$, $4.5$, $5.4$, and $6$, respectively.

by

$$\text{PSF}(\mathbf{x}) = (I^{c_1}(\mathbf{x}) - I^{c_2}(\mathbf{x})) * \left\{k_{\text{cam}}\pi^2 \left(J^{c_1}(\mathbf{x}) - J^{c_2}(\mathbf{x})\right)\right\}^{-1}, \tag{S.134}$$

where $\{\cdot\}^{-1}$ denotes the inverse (deconvolution) operation.

The PSF results obtained from the recorded fog data show that, as the optical thickness $OT$ increases, the peak amplitude of the delta-function component of the PSF decreases progressively, consistent with the corresponding growth of the attenuation term $e^{-\beta u(\mathbf{x})}$ in Eq. (S.133) (equivalently, the $e^{-OT_c}$ decay of Eq. (S.62)). No visually discernible broadening of the delta-function peak

is observed across the tested range, which is qualitatively consistent with the negligible-forward-scattering assumption adopted throughout this work. We note that this observation is illustrative rather than a quantitative measurement: no peak-width metric (e.g., full width at half maximum) was extracted, and at the highest tested optical thicknesses the delta-function peak approaches the noise floor, where a modest degree of broadening could in principle be masked by noise rather than genuinely absent. Correspondingly, from Fig. S.8**b** to **f**, the target channel-difference contrast decreases without any visually apparent edge blurring, offering a further qualitative indication that the fog medium does not substantially degrade the target's high-frequency information in this regime. Moreover, as the peak of the delta-function component continues to shrink with increasing fog density, it eventually becomes indistinguishable from the noise floor, a trend qualitatively consistent with the decrease of system SNR with optical thickness predicted by Eq. (S.68). Over this same range, the noise level in Fig. S.8**b** through **f** remains visually comparable, indicating that as increasing fog density sharply attenuates the ballistic signal, the change in ambient-light intensity and total image intensity is small enough that it does not produce an obviously discernible change in the resulting photon shot-noise level. Although the decay of the ballistic signal is the primary reason the delta-function component becomes submerged in noise, this decay is physically irreversible and cannot be compensated for; recovering a delta-function signal buried in noise can therefore only be approached by suppressing the noise itself, which is consistent with the conclusion, developed throughout this section, that dense-fog reconstruction fundamentally depends on noise suppression and SNR enhancement. Finally, by the properties of the convolution operation, once the delta-function component of the PSF is well above the noise floor, as in Fig. S.8**b–d**, the target's information is directly visible in the fog image without further processing.

### S.3.7 Validation Summary

The experiments support a sensor model in which the radiometric signal is converted to photoelectrons and then to DN, while the practical detection limit is determined by the measured noise floor together with shot and quantization noise. Cross-scene prediction, direct noise and SNR comparisons, frame averaging, and bit-depth scaling are all reproduced with small experimental errors. Shot noise contributes approximately $84\%$–$88\%$ of the modeled intrinsic temporal variance

at the tested operating points. It is therefore the dominant contribution, although the measurements do not require the stronger assumption of a purely shot-noise-limited sensor.

## S.4 Test Scenarios

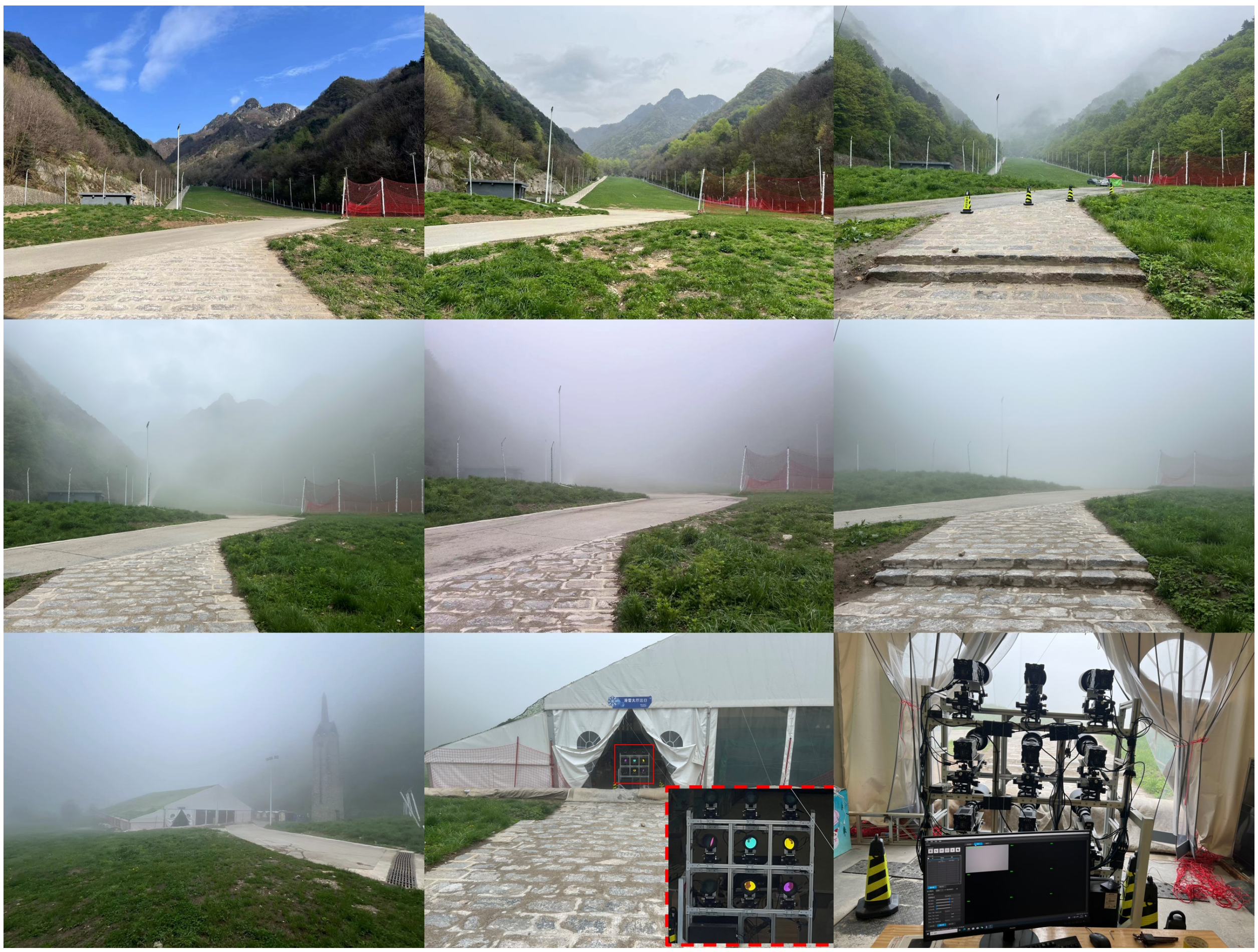

Figure S.9 **An illustration of mountainous testing scenario.**

The valley scene is situated in the Qinling Cuihua Mountain Landslide National Geopark, with a testing distance of 700 m and an altitude of approximately 1400 m. The observation elevation angle is set at 8 degrees. The primary objective is to evaluate the system's de-scattering performance under heavy fog conditions. The system operates continuously, day and night, with the acquisition software activated to capture images during foggy weather. Figure S.9 illustrates the visibility conditions of the site under different weather scenarios, as well as the layout of the observation area.

The road scene is located at the entrance of the Qinling Taiping National Forest Park, with a total length of 1,000 m, and observed from a horizontal perspective. Figure S.10 presents the observation conditions at the site under various weather scenarios (such as rain, freezing rain, snow, strong winds, and heavy fog), as well as the layout of the observation area, with continuous observation lasting over 15 days.

The indoor fog chamber and murky water testing scenarios are illustrated in Fig. S.11 and Fig. S.12, respectively.

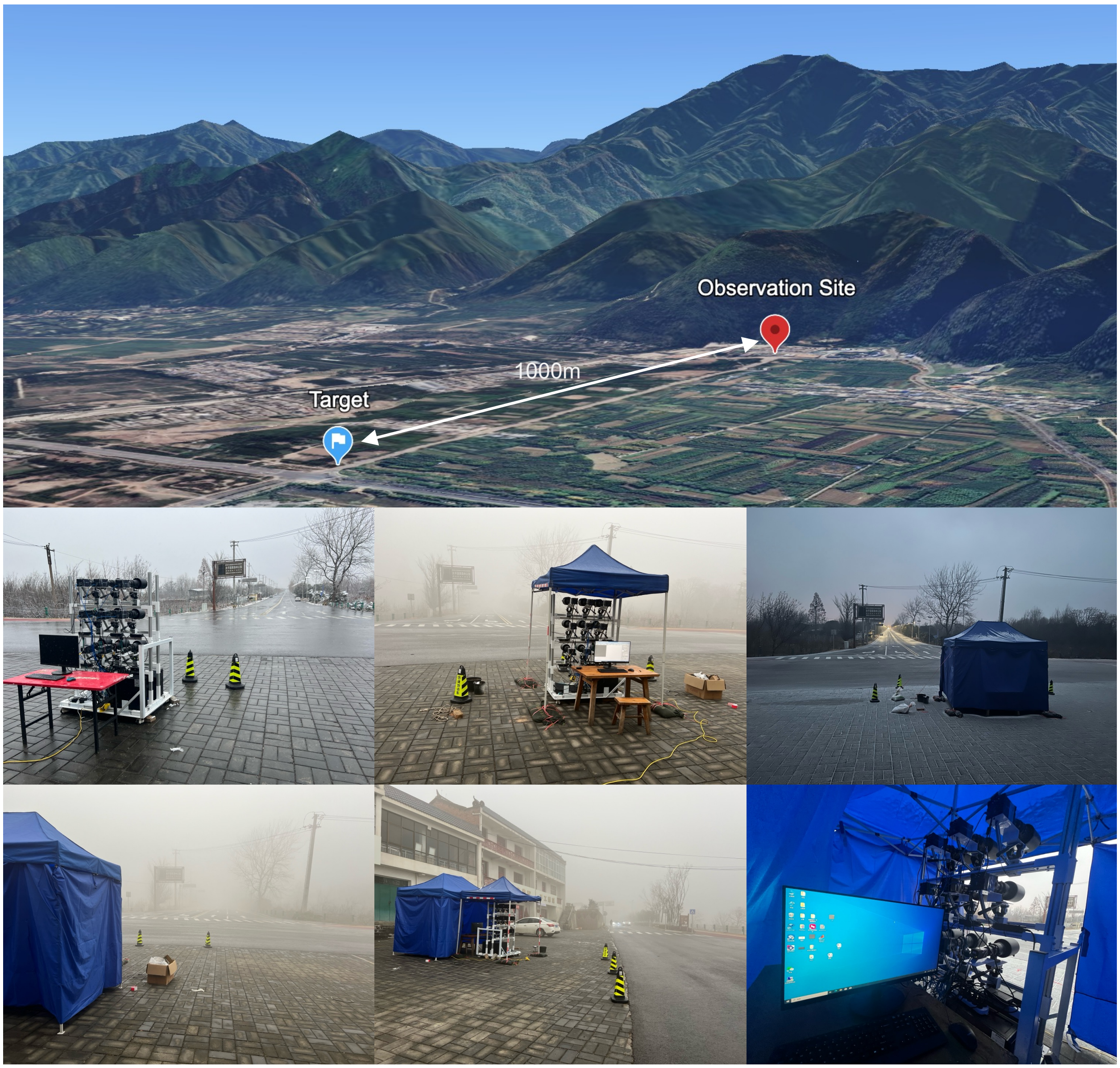


Figure S.10 **An illustration of road testing scenario**

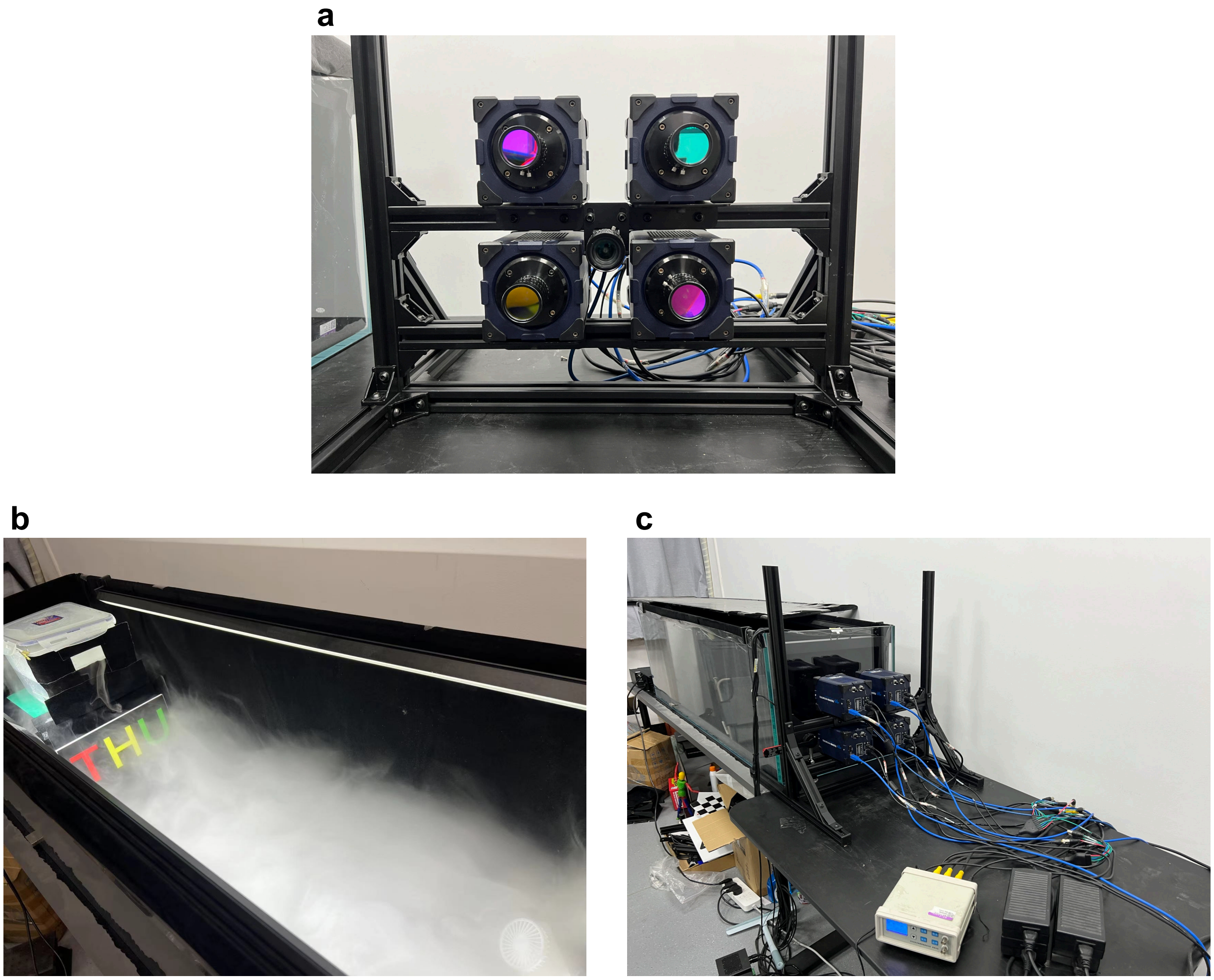


Figure S.11 **An illustration of indoor fog chamber testing scenario. a** Illustration of the indoor testing prototype of the CISA system's color imaging unit, which consists of four sCMOS cameras (Teledyne Photometrics Prime BSI) equipped with customized optical filters. The camera lenses are FA0802D lenses (focal length: 8 mm, sensor size: 2/3"). **b** Illustration of a schematic of the fog chamber used for indoor fog generation, featuring dimensions of 1.5 m × 0.5 m × 0.5 m, with fog produced by a 1.7 MHz ceramic nebulizer. **c** Overview of the indoor testing environment.

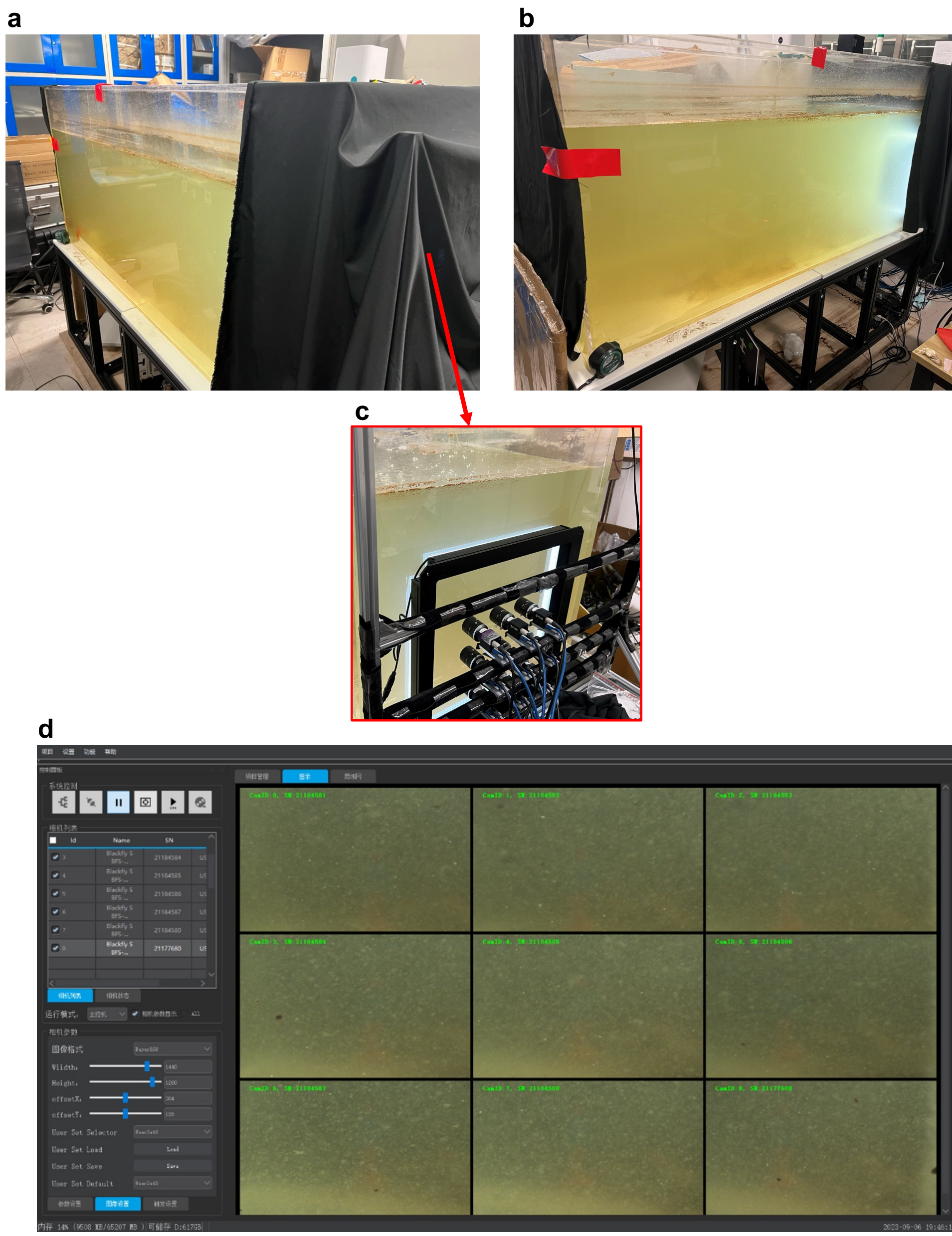


Figure S.12 **An illustration of murky water testing scenario. a-b**: The carp farming murky water body primarily consists of a high-transparency acrylic fish tank measuring 1.7 m × 0.7 m × 0.8 m, where we cultivate carp to create a murky environment. This water body contains scattering media with varying particle diameters, providing a more realistic underwater testing environment.**c** A miniaturized camera array de-scattering system featuring nine FLIR BFS-U3-31S4C cameras (12-bit depth), equipped with FA0802D lenses, with an inter-camera spacing of approximately 8 cm, along with a simulated underwater observation spotlight.**d** LUSTER Software Acquisition Interface Under Murky Water Body.

## S.5 Scattering Degree Estimation

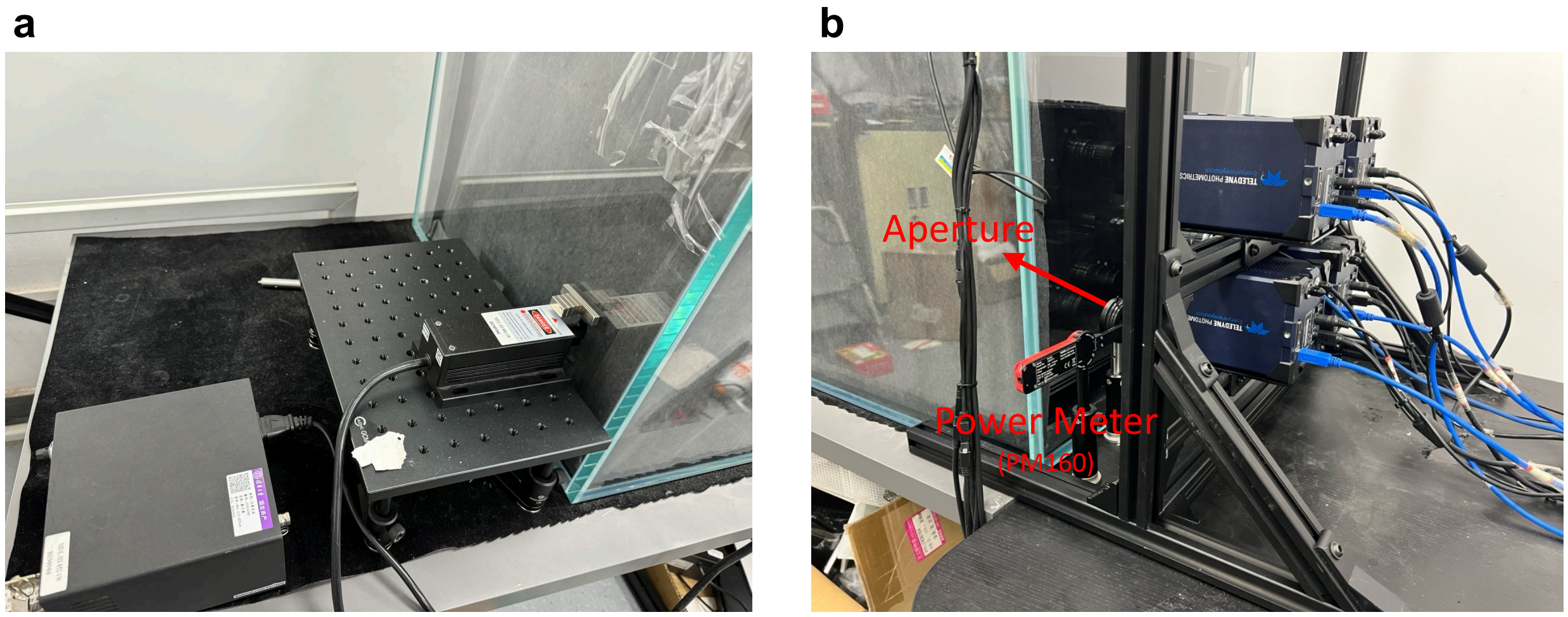


Figure S.13 **Measurement of the scattering degree. a** A photo of the employed 852 nm laser emitter (MDL-III-852-1W). **b** A photo of the Thorlabs PM-160 wireless power meter and aperture at the scattering light receiving end.

The optical thickness (OT) in the scattering medium can be expressed as:

$$OT = -\ln\left(\frac{I(d)}{I_0}\right), \tag{S.135}$$

where $I_0$ represents the initial light energy, and $I(d)$ is the remaining ballistic light energy after traveling a total distance $d$. The attenuation coefficient $\beta(\lambda)$ can be expressed as the sum of the absorption coefficient $a(\lambda)$ and the scattering coefficient $b(\lambda)$:

$$\beta(\lambda) = a(\lambda) + b(\lambda), \tag{S.136}$$

$\lambda$ represents the wavelength of light. In the scattering medium, it can also be expressed as:

$$\beta(\lambda) = -\frac{1}{d}\ln\left(\frac{I(d)}{I_0}\right). \tag{S.137}$$

We use the PM-160 wireless power meter to measure $I_0$ and $I(d)$. In the exposure logic test presented in Fig. 2 of the main text, global unified exposure and independent exposure are conducted separately, and the de-scattering results are compared under the same OT conditions.

For the murky water body tested, the same principle is used to determine $\beta(852\,\mathrm{nm})$ for the test medium. The beam attenuation coefficient is measured with a collimated 852 nm laser diode module (CNI MDL-III-852-1W) and a Thorlabs PM-160 power meter placed behind a 1 mm circular aperture, over a path of $d = 1.00\,\mathrm{m}$ through a high-transparency acrylic cell measuring $100\,\mathrm{cm} \times 15\,\mathrm{cm} \times 15\,\mathrm{cm}$. The detector half-acceptance angle is $\lesssim 10\,\mathrm{mrad}$ ($\lesssim 0.6°$), comparable to or narrower than that of standard oceanographic transmissometers (*21*), so the retrieved quantity approximates the beam attenuation coefficient $\beta$ rather than a diffuse attenuation coefficient.

The reference measurement $I_0$ is taken with the cell empty. Because water ($n \approx 1.329$ at 852 nm) is closer in refractive index to the acrylic cell ($n \approx 1.482$) than air is, the filled cell loses less light at its inner interfaces than the empty reference, by a factor of 1.074 (*22*). Correcting Eq. (S.137) for this interface-loss difference gives

$$\beta(852\,\mathrm{nm}) = 8.98\,\mathrm{m}^{-1}, \tag{S.138}$$

corresponding to an optical thickness over the 1.7 m observation path of

$$\tau = \beta L = 15.3 \tag{S.139}$$

attenuation lengths at 852 nm.

The coefficient $\beta(852\,\mathrm{nm})$ is measured in the near infrared, whereas the commonly quoted open-ocean and coastal seawater values ($0.15\,\mathrm{m}^{-1}$ and $0.298\,\mathrm{m}^{-1}$) refer to the blue–green band (*23*). Because the volume scattering phase function of the test medium also differs from that of marine hydrosols, a conversion to an equivalent open-ocean or coastal observation distance would mix spectral bands and would not correspond to equal image degradation; such a conversion is therefore not used to characterize the medium. We instead characterise the experiment directly in terms of optical thickness, and make the spectral decomposition explicit.

At 852 nm the absorption coefficient of pure water is $a_w = 4.25\,\mathrm{m}^{-1}$ (*24*), and absorption by CDOM and by phytoplankton pigments is negligible in the near infrared, so subtracting $a_w$ isolates the particulate attenuation coefficient,

$$\beta_p(852\,\mathrm{nm}) = \beta(852\,\mathrm{nm}) - a_w = 4.72\,\mathrm{m}^{-1}, \tag{S.140}$$

where $\beta_p = a_p + b_p$ combines particulate absorption $a_p$ and particulate scattering $b_p$. Particulate absorption by aquatic particles in the near-infrared is generally weak (*25*), so $a_p \ll b_p$ and $\beta_p \approx b_p$ at 852 nm. Because the suspended particles are large relative to the optical wavelength, their scattering is expected to vary more weakly with wavelength than the strong water-absorption variation in the near infrared. We therefore use the 852-nm particulate attenuation as an approximate measure of the scattering strength of the suspension, giving a scattering optical thickness

$$\tau_s \approx \beta_p L = 4.72\,\mathrm{m}^{-1} \times 1.7\,\mathrm{m} \approx 8.0, \qquad \text{(S.141)}$$

i.e., fewer than one photon in $10^3$ survives the particulate attenuation without interaction. This scattering optical thickness, reported alongside the total optical thickness at the measurement wavelength ($\tau = 15.3$, Eq. (S.139)), is used to characterize the difficulty of the de-scattering problem for this test medium.

## S.6 Design of CISA detection system

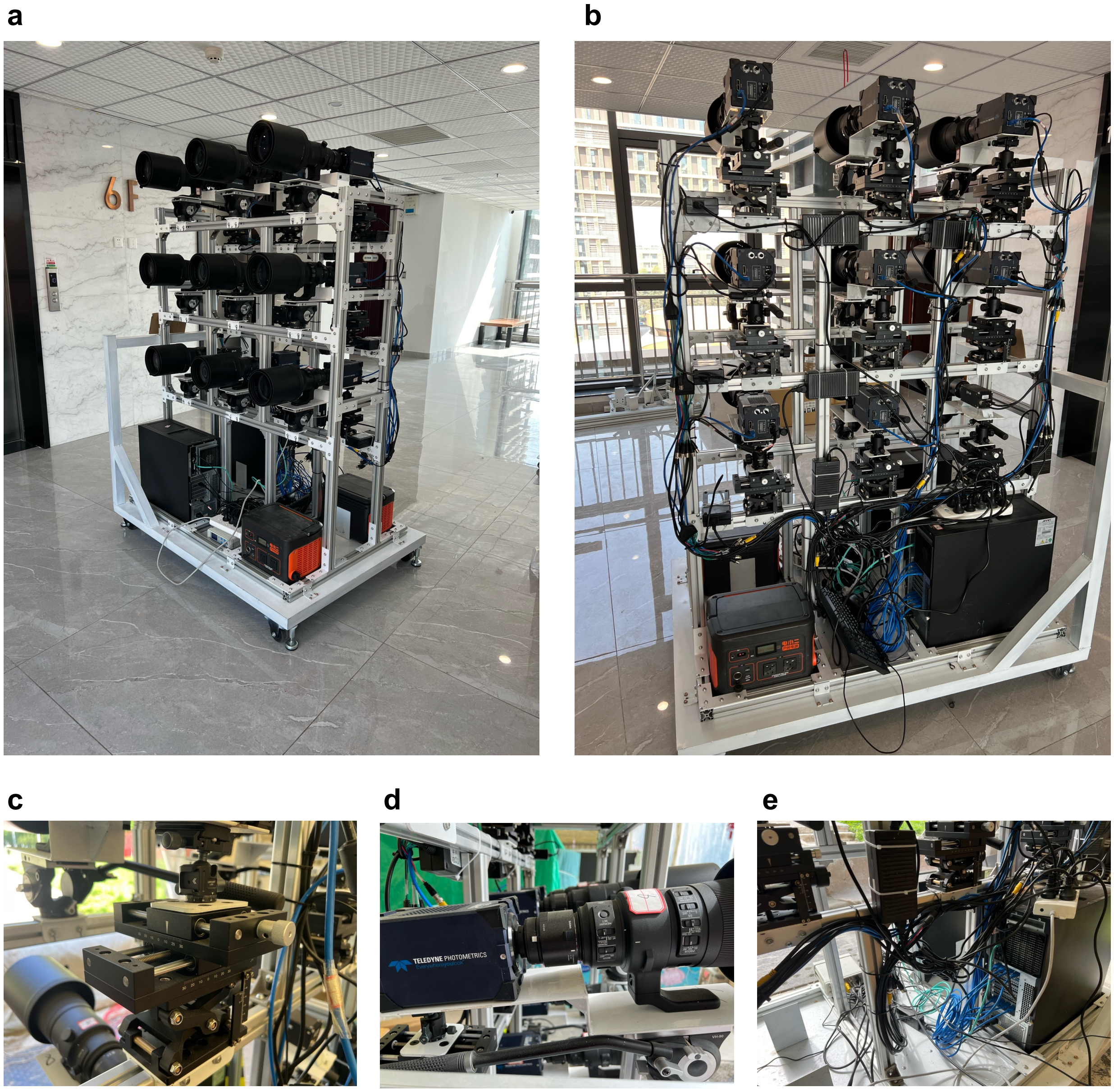


Figure S.14 **An illustration of the CISA detection system.** **a** Side View of the CISA System. **b** Rear View of the CISA System. **c** Camera gimbal and high-precision three-degree-of-freedom slider. **d** System observation angle adjustment, with an elevation angle of 8 degrees. Using different gimbals and fine-tuning sliders, the observation angle can be adjusted from -15 to 15 degrees. **e** Schematic wiring diagram between the dual host and the sCMOS camera array.

In Fig. S.14 and Fig. S.15, we present photographs of the CISA system and the information flow control. The main control host sends sequential commands to the signal synchronization trigger,

which encodes the shooting frame rate and required exposure time for each camera. Each camera captures images with its own exposure settings. The eight sCMOS cameras transmit image stream data close to their maximum capacity to the subordinate high-performance host, while the CMOS camera sends its data back to the main control host. Data collection is performed using Spinview software, and Lingyun Optical's LUSTER software is used for data visualization.

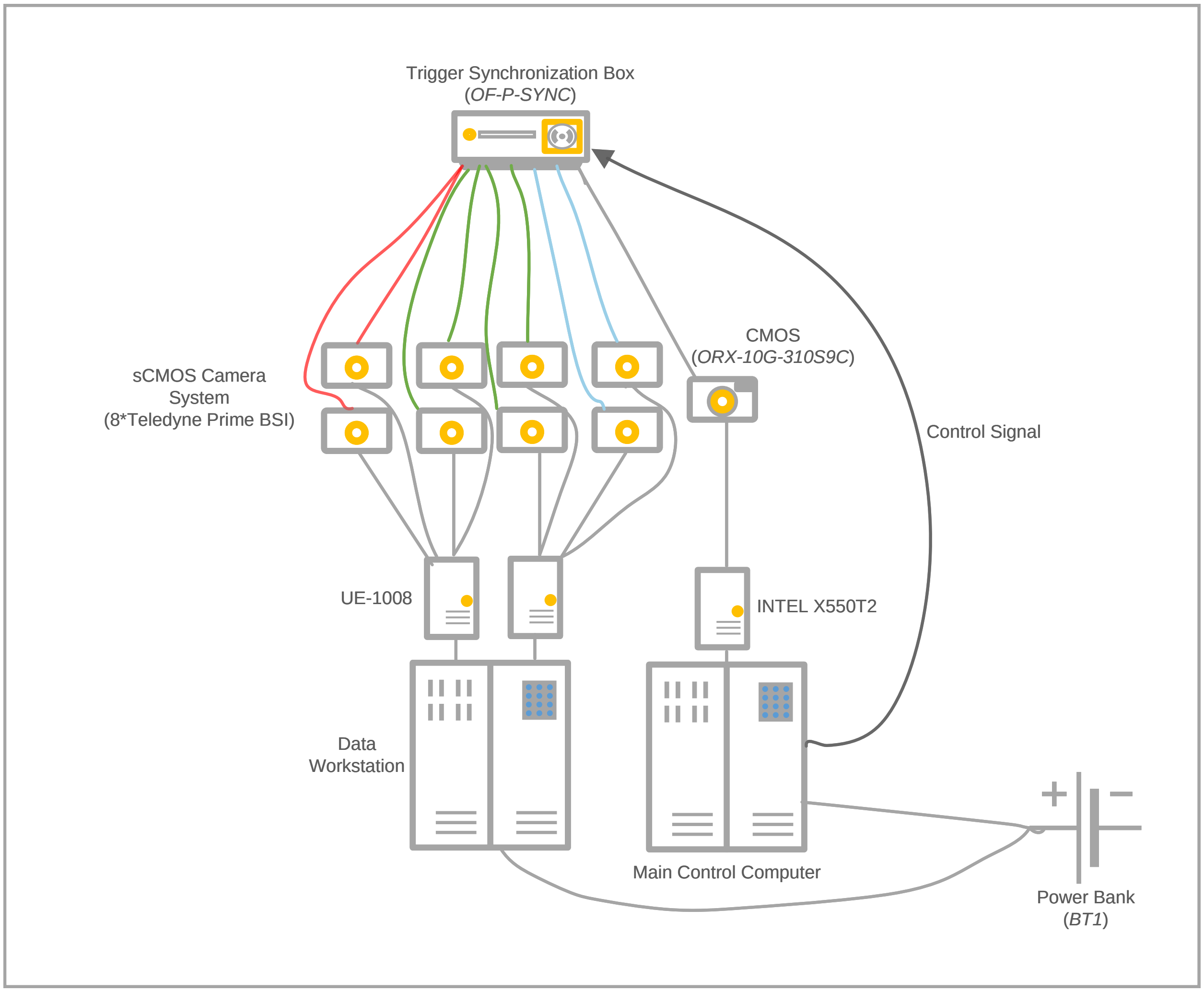


Figure S.15 **Schematic diagram of the information flow control within the system**

Table S.3 Specifications of the OF-P-SYNC-0800-02 trigger synchronization box.

| Parameter | Specification |
|---|---|
| Output signal | Square wave; 5 V logic high and 0 V logic low; 1 Hz to 999 Hz adjustable |
| Dimensions | 165 × 160 × 100 mm |
| Mass | $<$ 1000 g |
| Power supply | 12 V/1 A adapter |
| Ports | 8 BNC outputs and 2 BNC cascade ports |
| Synchronization error | $<$ 50 ns |
| Two-stage cascade delay | $<$ 600 ns |

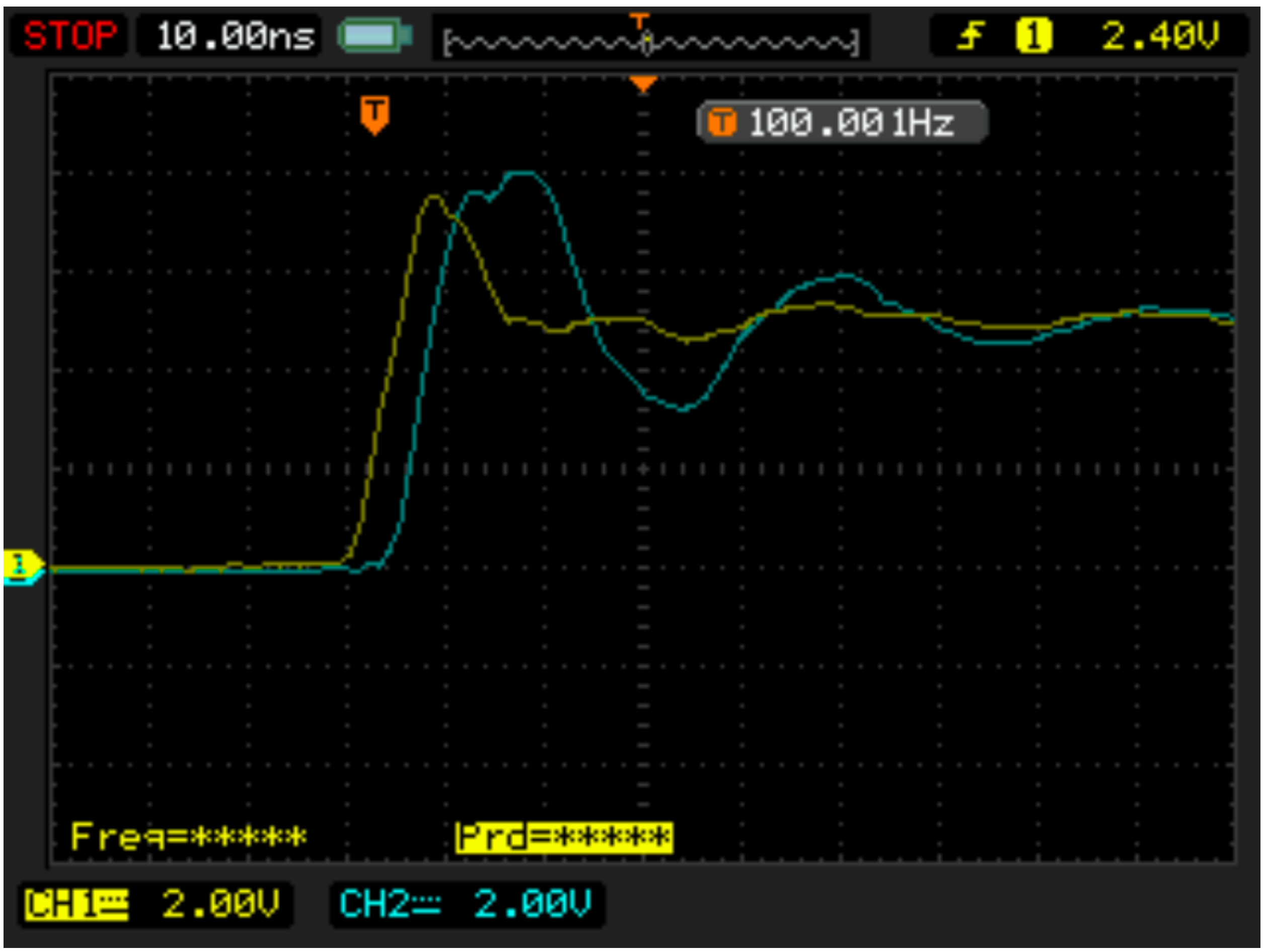


Figure S.16 **Trigger waveform of the synchronization trigger.**

All cameras are driven by a common hardware square-wave trigger generated by one OF-P-SYNC-0800-02 synchronization box. The controller provides eight BNC outputs, an adjustable frequency of 1 Hz to 999 Hz, and 5 V logic-high signals. According to the device specifications in Table S.3, the synchronization error between output channels is less than 50 ns, and the delay of two

cascaded controllers is less than $600\,\mathrm{ns}$. We also measured two output channels directly using an oscilloscope with a $10\ \mathrm{ns/div}$ time base. The measured rising-edge offset was consistent with the specified inter-channel error, as shown in Fig. S.16. Compared with the $600\,\mu\mathrm{s}$ to $1050\,\mu\mathrm{s}$ channel exposure times and the $100\,\mathrm{ms}$ frame period used in the outdoor experiments, the inter-channel trigger error is more than four orders of magnitude smaller than the exposure time and more than six orders of magnitude smaller than the frame period. It therefore has no measurable effect on spatial alignment between channels.

Table S.4 summarizes the scene conditions and acquisition settings used for the $700\,\mathrm{m}$ outdoor fog campaign. Visibility was estimated from roadside markers separated by $50\,\mathrm{m}$. The extinction coefficient and optical thickness were then obtained using the Koschmieder relation under the WMO meteorological optical range convention, with a contrast threshold of $\varepsilon = 5\%$, and the attenuation-measurement procedure described in Sec. S.5. This estimate assumes that the fog is homogeneous along the observation path. Ambient illuminance was not recorded directly, so the channel exposure times provide the relevant operational illumination reference.

Table S.4 Scene conditions and acquisition parameters for the $700\,\mathrm{m}$ outdoor fog campaign.

| Quantity | Value |
|---|---|
| Scene site | Cuihua Mountain Landslide National Geopark, Qinling Mountains |
| Observation distance | $700\,\mathrm{m}$ |
| Elevation angle | $8^\circ$ |
| Altitude | $\approx 1400\,\mathrm{m}$ |
| Weather | Overcast daylight, dense valley fog |
| Campaign period | April–May 2024 |
| Visibility $V$ ($50\,\mathrm{m}$ marker method) | $200\,\mathrm{m}$ to $700\,\mathrm{m}$ |
| Extinction coefficient $\beta$ | $4.3 \times 10^{-3}\,\mathrm{m}^{-1}$ to $1.5 \times 10^{-2}\,\mathrm{m}^{-1}$ |
| Optical thickness at $700\,\mathrm{m}$ | 3.0–10.5 |
| *Acquisition* | |
| Exposure time, R channel | $1050\,\mu\mathrm{s}$ |
| Exposure time, G1/G2 channels | $600\,\mu\mathrm{s}$ |
| Exposure time, B channel | $750\,\mu\mathrm{s}$ |
| Frame rate | $10\,\mathrm{Hz}$, synchronously triggered |
| Cameras $N_v$/frames per camera $N_f$ | 8/10 |
| Fused frames per reconstruction | 80 (20 R, 40 G, and 20 B) |
| Effective integration, R/G/B | $21\,\mathrm{ms}/24\,\mathrm{ms}/15\,\mathrm{ms}$ |
| Acquisition window per reconstruction | $1.0\,\mathrm{s}$ |
| Continuous recording per scene | $5\,\mathrm{min}$ to $10\,\mathrm{min}$ |
| Bit depth/format | 16-bit TIFF |

At $10\,\mathrm{Hz}$, ten frames from each of the eight cameras give an acquisition window of $1\,\mathrm{s}$ and 80 input frames per reconstruction. The channel allocation provides 20 red, 40 green, and 20 blue frames. Combined with the independent exposure times, this corresponds to effective integration times of $21\,\mathrm{ms}$, $24\,\mathrm{ms}$, and $15\,\mathrm{ms}$ for the red, green, and blue channels, respectively.

Table S.5 reports the main engineering characteristics of the CISA prototype. These values describe the research system used for methodological validation rather than intrinsic requirements of the imaging principle. In particular, the support frame was deliberately oversized to maintain mechanical stability during continuous unattended operation.

Table S.5 Engineering specifications of the CISA prototype.

| Quantity | Value |
|---|---|
| Total power consumption | $\approx 450\,\mathrm{W}$ |
| Dominant contribution | Two host workstations |
| Illumination | $0\,\mathrm{W}$ (outdoor system fully passive) |
| Processing speed, GPU (RTX 4090) | $\approx 25\,\mathrm{Hz}$ ($\approx 40\,\mathrm{ms}$ per frame) |
| Processing speed, CPU (MATLAB reference) | $\approx 2.5\,\mathrm{s}$ per frame |
| Acquisition window | $1.0\,\mathrm{s}$ |
| Raw data throughput | $\approx 640\,\mathrm{MiB\,s^{-1}}$ |
| Per frame | $8\,\mathrm{MiB}$ ($2048^2$ pixels, 16-bit) |
| Per reconstruction | $640\,\mathrm{MiB}$ (80 frames) |
| Overall dimensions | $1000 \times 1000 \times 1800\,\mathrm{mm}$ |
| Mass | $\approx 160\,\mathrm{kg}$ |
| Inter-camera spacing | $20\,\mathrm{cm}$ |
| Operating temperature range | $-20\,^{\circ}\mathrm{C}$ to $30\,^{\circ}\mathrm{C}$ |
| Approximate component cost | ≈US$50,000 |

The end-to-end latency is dominated by the $1\,\mathrm{s}$ acquisition window. GPU reconstruction requires approximately $40\,\mathrm{ms}$ per output frame, while the remaining time is associated with data transfer and pipeline overhead. The measured power, dimensions, and mass are mainly determined by the two workstations and the support frame. The outdoor optical system itself uses no active illumination and remains fully passive.

## S.7 Resolution Budget of the CISA Detection System

This section separates the diffraction limit, detector sampling limit, and smallest feature recovered after de-scattering at the field-experiment distance of $R = 700\,\mathrm{m}$. The approximately $2\,\mathrm{cm}$ value reported in the main text refers to the smallest resolved chart feature, not to the optical or detector cutoff.

### S.7.1 Instrumental resolution

Each channel uses a SIGMA 500 mm F4 objective with a $2\times$ teleconverter. The teleconverter doubles the focal length and working $f$-number but leaves the entrance pupil unchanged:

$$D = \frac{500\,\mathrm{mm}}{4} = 125\,\mathrm{mm}, \qquad f = 1000\,\mathrm{mm}, \qquad f/\# = \frac{f}{D} = 8. \tag{S.142}$$

The detector has $2048 \times 2048$ pixels with pitch $p = 6.5\,\mu\mathrm{m}$. The main parameters are listed in Table S.6. These values give a single-channel field of view of $0.763°$ and a footprint of $9.32\,\mathrm{m}$ at $700\,\mathrm{m}$, consistent with the smaller common field of view of the converging camera array.

Table S.6 Parameters used in the resolution budget.

| Symbol | Quantity | Value | Basis |
|---|---|---|---|
| $f$ | Effective focal length | $1000\,\mathrm{mm}$ | 500 mm objective with $2\times$ teleconverter |
| $D$ | Entrance-pupil diameter | $125\,\mathrm{mm}$ | $500/\mathrm{F}4$; unchanged by the teleconverter |
| $f/\#$ | Working $f$-number | 8 | $f/D$ |
| $p$ | Detector pitch | $6.5\,\mu\mathrm{m}$ | Prime BSI detector |
| $N$ | Pixels per side | 2048 | Prime BSI detector |
| $R$ | Object distance | $700\,\mathrm{m}$ | Field experiment |
| $\lambda$ | Band centers (B/G/R) | 450/550/630 nm | Custom bandpass filters |

For incoherent imaging through a clear circular pupil, the object-space cutoff period and Rayleigh separation are (*26*)

$$\Lambda_{\min}^{\mathrm{diff}} = \frac{\lambda R}{D}, \qquad \boxed{\delta_{\mathrm{diff}} = \frac{1.22\,\lambda R}{D}}. \tag{S.143}$$

Table S.7 shows that the Rayleigh separation is $3.1\,\mathrm{mm}$ to $4.3\,\mathrm{mm}$ across the three bands.

Table S.7 Diffraction and detector-sampling quantities at $R = 700\,\mathrm{m}$.

| Band | $\lambda$ (nm) | $\nu_c^{\mathrm{obj}}$ ($\mathrm{cycle\,m^{-1}}$) | $\Lambda_{\mathrm{min}}^{\mathrm{diff}}$ (mm) | $\delta_{\mathrm{diff}}$ (mm) | $Q$ |
|---|---|---|---|---|---|
| B | 450 | 396.8 | 2.52 | 3.07 | 0.554 |
| G | 550 | 324.7 | 3.08 | 3.76 | 0.677 |
| R | 630 | 283.4 | 3.53 | 4.30 | 0.775 |
| Detector Nyquist frequency | $\nu_{\mathrm{Nyq}}^{\mathrm{obj}} = 109.9\,\mathrm{cycle\,m^{-1}}$, $\Lambda_{\mathrm{Nyq}} = 9.10\,\mathrm{mm}$ (ideal period) | | | | |

The object-space ground sampling distance is

$$\mathrm{GSD} = \frac{pR}{f} = 4.55\,\mathrm{mm}. \tag{S.144}$$

The ideal Nyquist criterion requires two samples per spatial period. The minimum period is therefore $2\,\mathrm{GSD} = 9.10\,\mathrm{mm}$, corresponding to a bar width of one GSD, or one pixel per bar. This limit depends on favorable alignment between the bar pattern and the pixel grid. Using the more robust criterion of two pixels per bar, the minimum feature width is

$$\delta_{\mathrm{samp}} = 2\,\mathrm{GSD} = 9.10\,\mathrm{mm}. \tag{S.145}$$

Hereafter, the resolution values are compared as feature widths. The Rayleigh value is formally a two-point separation rather than a bar width, but this distinction does not affect the limiting mechanism because the diffraction value remains well below the sampling limit.

The sampling parameter is (*27*)

$$Q = \frac{\lambda(f/\#)}{p}. \tag{S.146}$$

The values $Q = 0.55$–$0.78$ are below the critical value of 2, so the channels are sampling-limited rather than diffraction-limited. The practical instrumental limit is therefore

$$\delta_{\mathrm{inst}} = \max(\delta_{\mathrm{diff}}, \delta_{\mathrm{samp}}) = 9.10\,\mathrm{mm}. \tag{S.147}$$

We use the larger value because diffraction and sampling are alternative resolution limits. Combining them in quadrature using the green-band Rayleigh value would give $9.9\,\mathrm{mm}$ and would not change the conclusion.

Multi-camera averaging mainly improves SNR and reduces sensitivity to sampling phase. A

strict super-resolution gain is not included in this conservative value.

### S.7.2 Atmospheric turbulence

For uniform Kolmogorov turbulence, the plane-wave and spherical-wave Fried parameters are (*28*)

$$r_0^{(\mathrm{pw})} = \left(0.423\, k^2 C_n^2 R\right)^{-3/5}, \qquad r_0^{(\mathrm{sw})} = \left(0.423\, k^2 C_n^2 R \frac{3}{8}\right)^{-3/5} \approx 1.80\, r_0^{(\mathrm{pw})}, \tag{S.148}$$

where $k = 2\pi/\lambda$. The finite-range target is better represented by the spherical-wave form, while the plane-wave form provides a conservative bound. The corresponding long-exposure FWHM scale is (*29*)

$$\boxed{\delta_{\mathrm{turb}} \approx \frac{0.98\,\lambda R}{r_0}}. \tag{S.149}$$

The reconstruction combines 10 frames per camera over $1\,\mathrm{s}$, and the camera baseline is $20\,\mathrm{cm}$. The fused result therefore averages partially decorrelated turbulence realizations. Table S.8 gives estimates at $550\,\mathrm{nm}$ over a range of $C_n^2$ values.

Table S.8 Estimated turbulence-limited resolution at $R = 700\,\mathrm{m}$ and $\lambda = 550\,\mathrm{nm}$.

| $C_n^2$ ($\mathrm{m}^{-2/3}$) | $r_0^{(\mathrm{pw})}$ (cm) | $\delta_{\mathrm{turb}}^{(\mathrm{pw})}$ (mm) | $r_0^{(\mathrm{sw})}$ (cm) | $\delta_{\mathrm{turb}}^{(\mathrm{sw})}$ (mm) |
|---|---|---|---|---|
| $1 \times 10^{-16}$ | 44.44 | 0.8 | 80.05 | 0.5 |
| $1 \times 10^{-15}$ | 11.16 | 3.4 | 20.11 | 1.9 |
| $1 \times 10^{-14}$ | 2.80 | 13.5 | 5.05 | 7.5 |
| $5 \times 10^{-14}$ | 1.07 | 35.3 | 1.92 | 19.6 |

The campaign was conducted under overcast conditions with dense valley fog. Such fog can occur in a stable boundary layer with weak turbulence, although the turbulence strength remains dependent on the site and the stage of fog development (*30, 31*). We therefore use $C_n^2 = 1 \times 10^{-15}\,\mathrm{m}^{-2/3}$ as a representative weak-turbulence case and retain $1 \times 10^{-14}\,\mathrm{m}^{-2/3}$ as a pessimistic bound. The spherical-wave estimates are then $1.9\,\mathrm{mm}$ and $7.5\,\mathrm{mm}$, respectively. Both are below the instrumental limit, indicating that turbulence was unlikely to be the main resolution limit. The path was inclined by approximately $8^\circ$ over valley terrain, and no scintillometer was deployed; the uniform-$C_n^2$ model should therefore be read as a bound rather than a measurement of the actual

path.

### S.7.3 Combined budget and measured chart

For an approximate engineering budget, the instrumental, turbulence, and residual-scattering scales are combined in quadrature:

$$\delta_{\rm sys}^2 \approx \delta_{\rm inst}^2 + \delta_{\rm turb}^2 + \delta_{\rm scat}^2. \tag{S.150}$$

Equation (S.150) treats the contributions as equivalent Gaussian blur scales. It is an engineering approximation: detector sampling is a bandwidth and aliasing limit rather than a convolution blur, so the equation is used only for an order-of-magnitude comparison and not as a rigorous MTF model.

For the representative and pessimistic spherical-wave cases, the instrumental and turbulence terms give

$$\sqrt{\delta_{\rm inst}^2 + \delta_{\rm turb}^2} = \begin{cases} 9.29\,\mathrm{mm}, & C_n^2 = 1\times10^{-15}\,\mathrm{m}^{-2/3}, \\ 11.8\,\mathrm{mm}, & C_n^2 = 1\times10^{-14}\,\mathrm{m}^{-2/3}. \end{cases} \tag{S.151}$$

Because $20\,\mathrm{mm}$ was the finest feature on the chart, the experiment establishes $\delta_{\rm sys} \le 20\,\mathrm{mm}$ rather than an equality. Equation (S.150) therefore gives only an upper bound on the equivalent residual-scattering scale:

$$\delta_{\rm scat} \le \sqrt{(20\,\mathrm{mm})^2 - \delta_{\rm inst}^2 - \delta_{\rm turb}^2} \le 17.8\,\mathrm{mm}. \tag{S.152}$$

The last value is the conservative limit obtained by omitting the nonnegative turbulence term. It is consistent with residual scattering but does not prove that scattering alone set the resolution.

The finest grid-chart element had a line width of $w = 20\,\mathrm{mm}$. At $700\,\mathrm{m}$,

$$w = 4.40 \text{ pixels}, \qquad 2w = 40\,\mathrm{mm} = 8.79 \text{ pixels}, \qquad \nu = 25.0\,\mathrm{cycle\,m}^{-1}. \tag{S.153}$$

The chart frequency therefore lies well below the detector Nyquist frequency: the finest chart element was substantially oversampled, at 8.79 pixels per period. The reported $2\,\mathrm{cm}$ resolution is

Table S.9 Approximate resolution budget at $R = 700\,\mathrm{m}$ and $\lambda = 550\,\mathrm{nm}$. The scattering contribution is a chart-based upper bound.

| Term | Origin | Contribution (mm) |
|---|---|---|
| $\delta_{\mathrm{diff}}$ | Diffraction for $D = 125\,\mathrm{mm}$ | 3.8 |
| $\delta_{\mathrm{samp}}$ | Practical detector sampling, $2\,\mathrm{GSD}$ | 9.1 |
| $\delta_{\mathrm{inst}}$ | **Instrumental limit** | **9.1** |
| $\delta_{\mathrm{turb}}$ | Spherical-wave estimate: representative/pessimistic | 1.9/7.5 |
| $\delta_{\mathrm{scat}}$ | Equivalent residual-scattering upper bound | $\leq 17.8$ |
| $\delta_{\mathrm{sys}}$ | **Chart-based resolution bound** | $\leq 20$ |

the smallest calibrated feature that remained visible after de-scattering. Because it was also the finest feature on the chart, the experiment shows that the minimum resolvable feature was no larger than $2\,\mathrm{cm}$; it does not determine the exact MTF cutoff.

This result is distinct from the fusion size error of less than $2\,\mathrm{cm}$ at $1000\,\mathrm{m}$. At that distance, the GSD is $6.50\,\mathrm{mm}$, so the stated fusion error corresponds to about 3.08 pixels of residual inter-camera misalignment. It describes registration accuracy rather than spatial resolution.

Overall, the system is sampling-limited at the instrument level, with a practical feature-width limit of $9.1\,\mathrm{mm}$. Under the representative fog-condition estimate, turbulence contributes about $1.9\,\mathrm{mm}$ and is not the limiting factor. The chart establishes only that the minimum resolvable feature was no larger than $20\,\mathrm{mm}$, which bounds the equivalent residual-scattering scale at $17.8\,\mathrm{mm}$. The reported $2\,\mathrm{cm}$ value is therefore a conservative, target-limited result that lies 2.2 times above the instrumental limit; the system was not operating at its optical limit.

## S.8 System De-scattering Capability Analysis

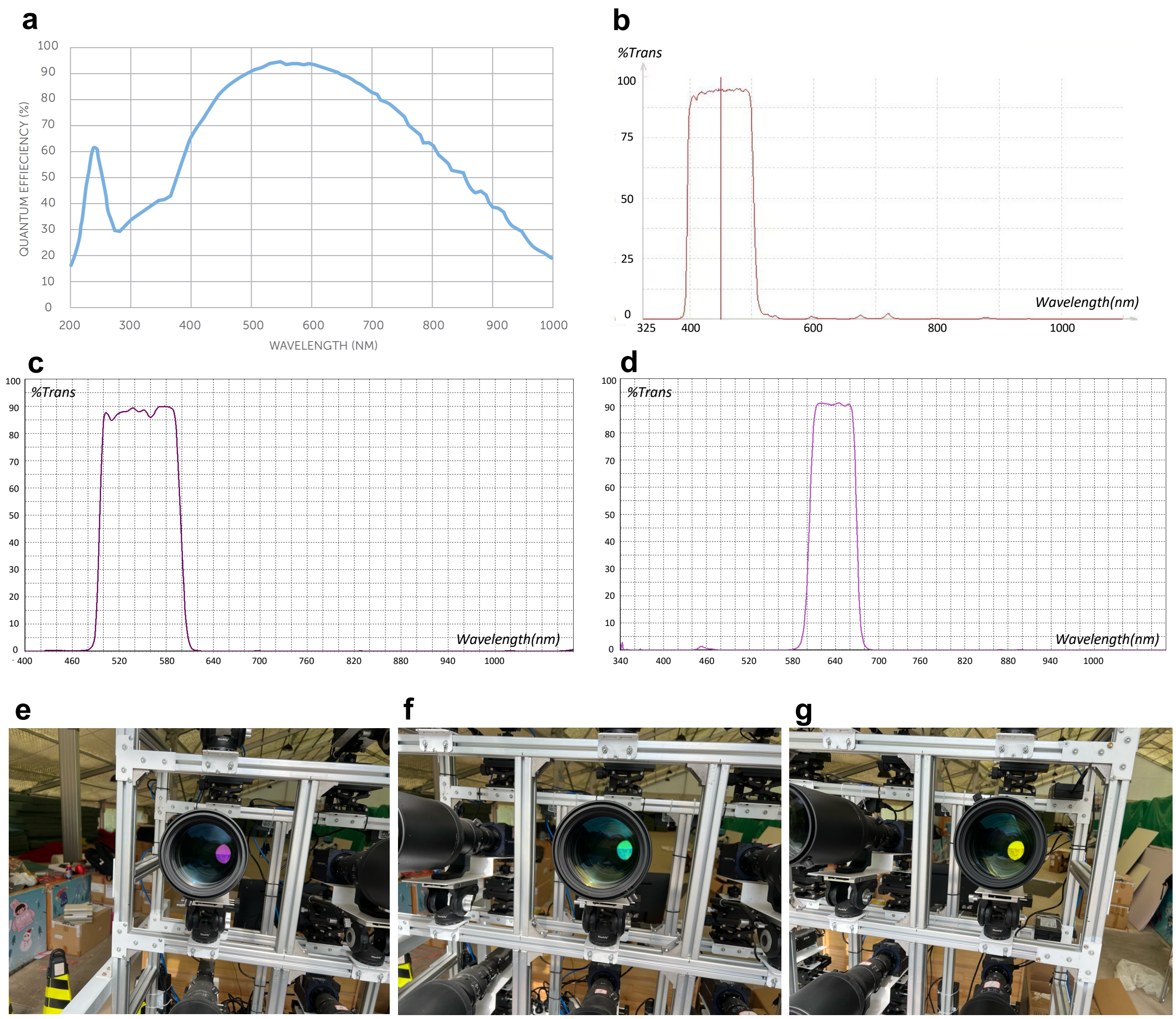


Figure S.17 **Diagram of the CISA detection system's optical filter bandpass and sCMOS spectral response. a** sCMOS spectral response.**b** Red bandpass range. **c** Green bandpass range. **d** Blue bandpass range. **e** Side view of the lens with a red filter. **f** Side view of the lens with a green filter. **g** Side view of the lens with a blue filter.

The prerequisite for the CISA system to achieve effective color imaging in highly scattering environments is that its color performance must be largely consistent with that of conventional CMOS cameras, capturing the true colors of the target to the greatest extent and making it suitable for human observation. We conducted color testing for the CISA system.Figure S.17 show the CISA detection system's optical filter bandpass. Figure S.18 **a** shows the indoor low-light imaging test of the CISA imaging unit, with the imaging target being colorful ”THU” letters. The CISA system

is highly sensitive to the reflected highlights of the target, and at its operating depth, it is able to restore the texture and color information of the target with high fidelity. Figure S.18 **(b-c-d-e)** present the imaging results of color charts on roads and in valleys. The images inside the red box are the color images output after the fusion of multiple sCMOS cameras in the CISA system, while the images outside the red box are the imaging results from a factory-calibrated CMOS camera (FLIR ORX-10G-310S9C). From extensive testing, it is clear that our CISA system, by using multiple sCMOS cameras and image optical flow remapping to output Raw images, followed by ISP processing steps, produces images nearly identical to those from a standard CMOS camera, with a high level of confidence in the overall image performance.

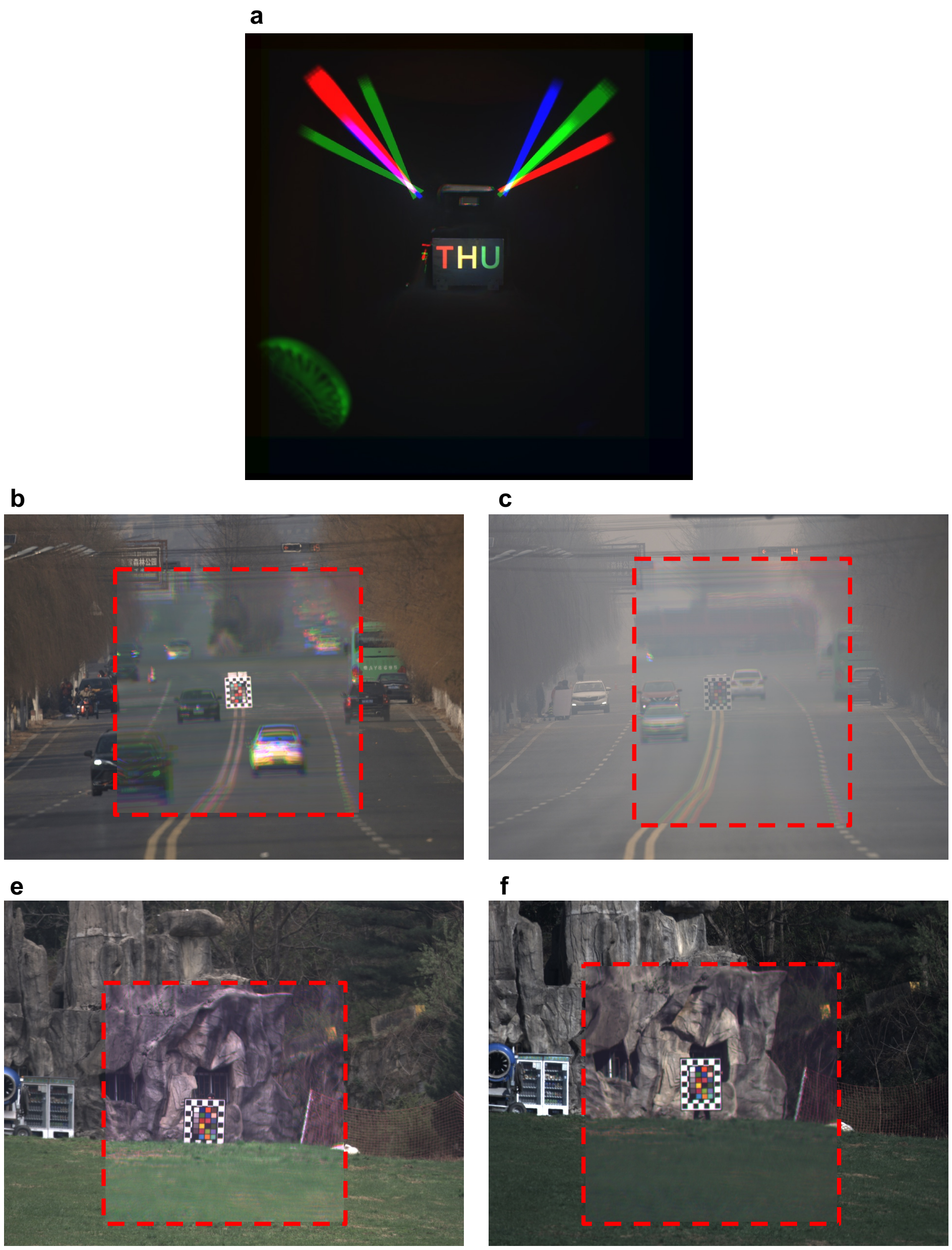


Figure S.18 **Color performance of the CISA detection system.**

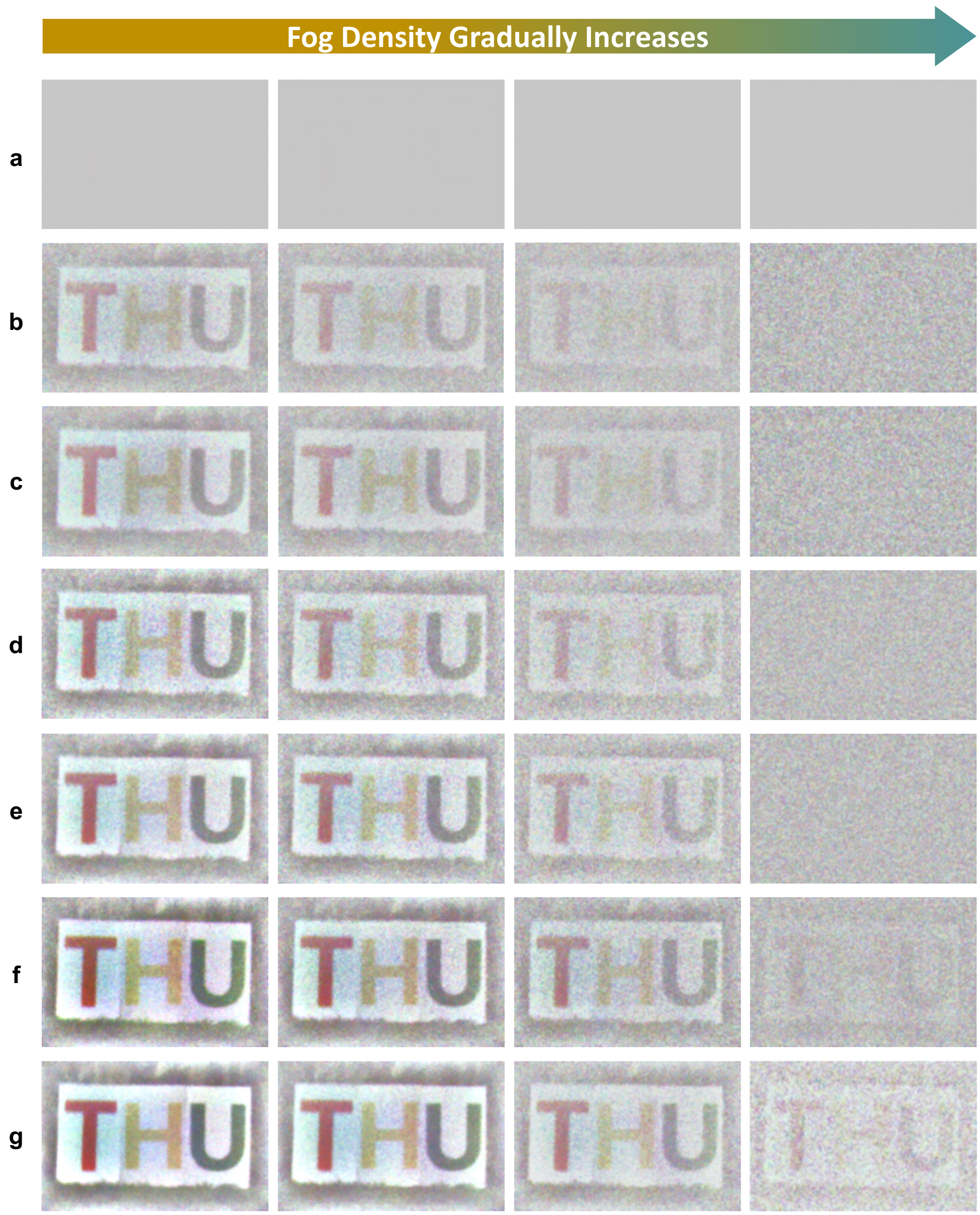


Figure S.19 **Effect of Fusion Strategy on De-scattering Performance. a** Scattered image. **b** De-scattering results from a single imaging unit with 1-frame fusion. **c** De-scattering results from a single imaging unit with 2-frame fusion. **d** De-scattering results from dual imaging units with 1-frame fusion. **e** De-scattering results from dual imaging units with 2-frame fusion. **f** De-scattering results from a single imaging unit with 10-frame fusion. **g** De-scattering results from dual imaging units with 10-frame fusion.

A single imaging unit refers to a set of four sCMOS cameras equipped with RGGB filters. The CISA system has a total of two color imaging units. Experimental results Fig S.19 indicate that the greater the number of fused frames, the better the overall de-scattering effect. Therefore,

within the same shooting time, using more imaging units can effectively improve de-scattering performance and smooth out the uneven distribution of highlights in the background. However, this benefit exhibits a trend of diminishing marginal returns, necessitating a balance between system complexity and de-scattering performance.

## S.9 Quantitative validation of color reconstruction

We evaluated color reconstruction from four aspects: the spectral response of the imaging system, color rendering under clear conditions, color preservation through fog, and person detection using the recovered images. Color differences are reported using CIEDE2000 ($\Delta E_{00}$), together with its weighted lightness, chroma, and hue components where appropriate.

### S.9.1 Spectral response of the imaging system

Each color channel in the CISA array uses a monochrome sCMOS camera and a customized bandpass filter. The spectral response of channel $c$ is

$$S_c(\lambda) = T_{\text{filter},c}(\lambda) QE(\lambda), \qquad c \in \{R, G, B\}, \tag{S.154}$$

where $T_{\text{filter},c}(\lambda)$ is the measured filter transmission and $QE(\lambda)$ is the spectral response of the sCMOS sensor. The measured passbands and sensor response are shown in Fig. S.17. The sensor responds over approximately $200\,\text{nm}$ to $1000\,\text{nm}$. The blue, green, and red filters transmit approximately $400\,\text{nm}$ to $500\,\text{nm}$, $500\,\text{nm}$ to $600\,\text{nm}$, and $580\,\text{nm}$ to $680\,\text{nm}$, respectively. These passbands cover most of the visible spectrum and enable color imaging. The filters define the three color bands, while the same sensor response applies to all channels. The recorded color is therefore determined by the optical passbands rather than by a color-filter array on the detector.

### S.9.2 ColorChecker comparison under clear conditions

A 24-patch ColorChecker Classic target was recorded synchronously by CISA and a commercial CMOS camera under the same clear-condition illumination. Patch means were measured from the central area of each patch and compared with the manufacturer's D50 CIE Lab values. Patches

2, 7, 12, 16, and 19 were excluded because at least one channel was clipped. White balance was estimated only from neutral patches 20–23 using a diagonal transform. The remaining 14 chromatic patches formed an independent validation set.

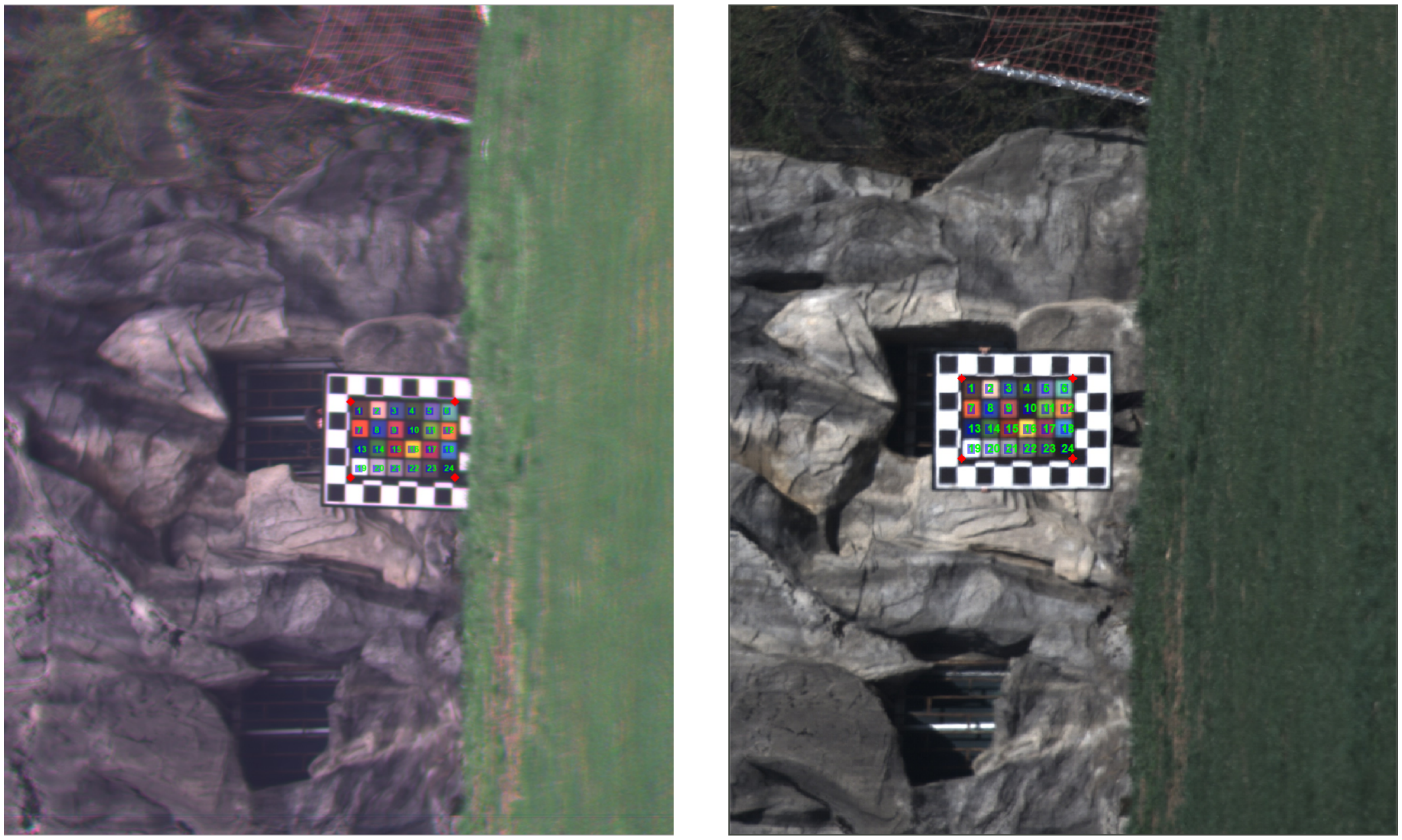

Figure S.20 **ColorChecker acquisition and ROI registration.** The left and right panels show the CISA and commercial CMOS measurements, respectively. Red markers indicate the registered chart corners, and the numbered overlays identify the 24 measured patches.

Table S.10 ColorChecker results after white balance. Weighted component values follow the CIEDE2000 formulation. Gain standard deviations were obtained by leave-one-out cross-validation over neutral patches 20–23.

| Metric | CISA (sCMOS) | Commercial CMOS |
|---|---|---|
| Mean $\Delta E_{00}$, chromatic patches | 8.939 | 8.997 |
| Mean $\lvert\Delta L'/S_L\rvert$ | 5.810 | 6.061 |
| Mean $\lvert\Delta C'/S_C\rvert$ | 4.092 | 3.745 |
| Mean $\lvert\Delta H'/S_H\rvert$ | 3.824 | 4.174 |
| Red gain $g_R$ | 0.6662 | 0.9358 |
| Blue gain $g_B$ | 0.6944 | 0.8381 |
| $\sigma(g_R)$ | 0.0335 | 0.0435 |
| $\sigma(g_B)$ | 0.0153 | 0.0059 |
| Mean held-out neutral $\Delta E_{00}$ | 5.788 | 4.165 |

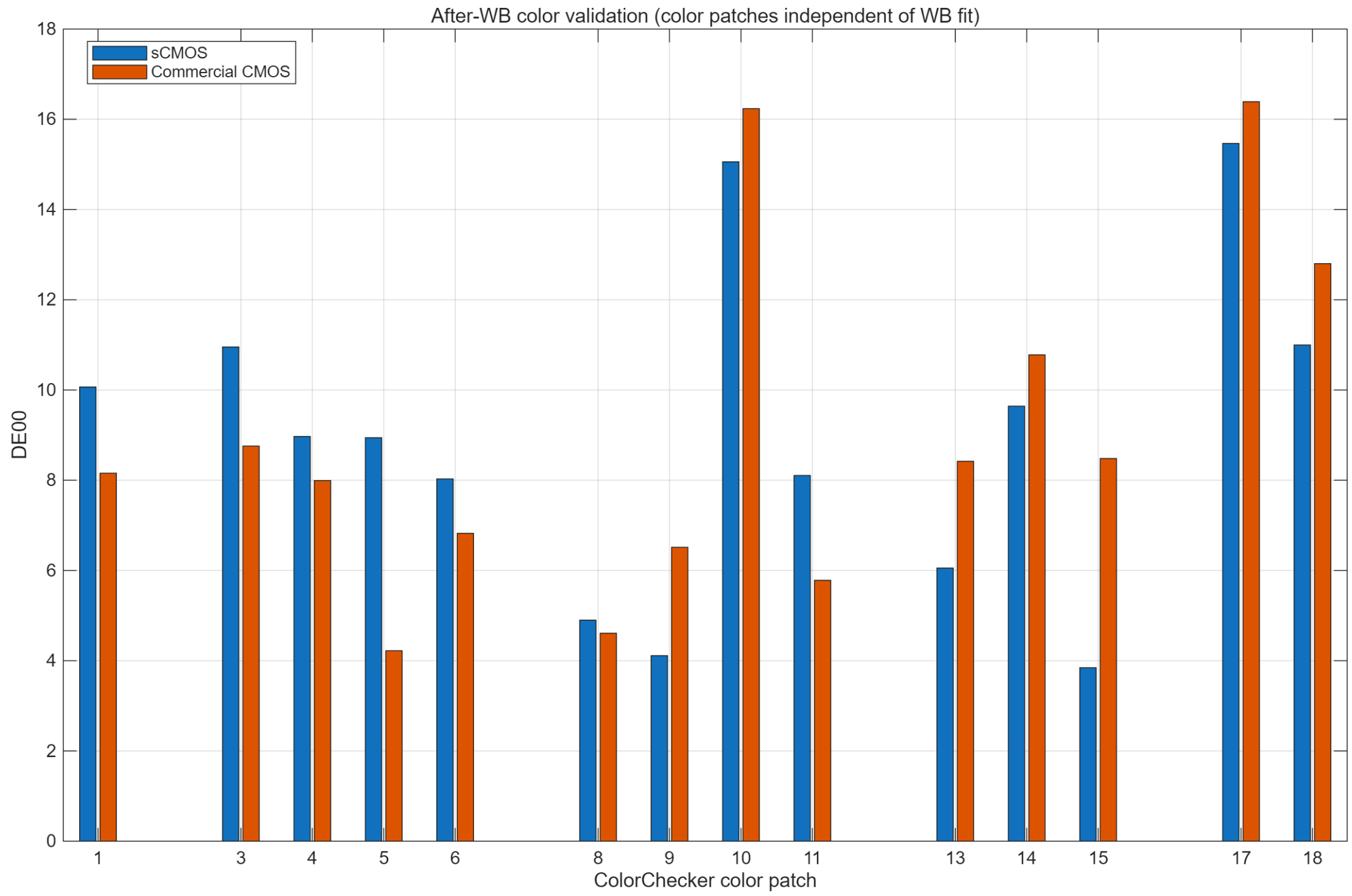


Figure S.21 **Patchwise color difference after white balance.** The $\Delta E_{00}$ values are reported for the 14 chromatic patches that were independent of the white-balance fit.

The patchwise results in Fig. S.21 show similar error distributions for the two systems, with the largest errors occurring on several of the same patches. Neither system gives consistently lower errors across all colors. Their mean $\Delta E_{00}$ values differ by only 0.059, indicating comparable color rendering without scattering. The leave-one-out results also show that the red gain is more sensitive to the choice of neutral reference than the blue gain. These measurements used 8-bit images without a color-correction matrix, and the commercial-camera chart crop was $112 \times 84$ pixels. The results therefore provide a controlled comparison between the two imaging chains rather than a certification of absolute colorimetric accuracy.

### S.9.3 Color preservation through fog

Clear-condition commercial-camera images of the same traffic targets were used as references. Four regions (`go`, `stop`, `turn`, and `40`) were measured at four increasing optical thicknesses. Region means were converted to CIE Lab after a common lightness normalization. Because hue becomes ill-defined as chroma approaches zero, we use the absolute hue-angle deviation $|\Delta h_{ab}|$ as

the primary measure and report the chroma retention $C^*_{\mathrm{test}}/C^*_{\mathrm{ref}}$ separately.

Table S.11 Mean absolute hue-angle deviation from the clear-condition reference, averaged over four traffic-sign regions. Lower values indicate better hue preservation. Values for the nearly achromatic scattered images are included only for reference.

| Method | Level 1 | Level 2 | Level 3 | Level 4 | Mean |
|---|---|---|---|---|---|
| Scattered image | 35.28 | 67.49 | 75.29 | 80.48 | 64.63 |
| CMOS + Rop$^+$ | 86.29 | 89.98 | 92.95 | 119.83 | 97.26 |
| CISA + Rop$^+$ | 69.59 | 80.90 | 90.02 | 97.12 | 84.41 |
| CISA + PCDP | **21.50** | **22.67** | **33.62** | **34.83** | **28.15** |

Table S.12 Mean chroma retention relative to the clear-condition reference. Unity indicates matched chroma.

| Method | Level 1 | Level 2 | Level 3 | Level 4 |
|---|---|---|---|---|
| Scattered image | 0.01 | 0.01 | $\approx 0$ | 0.00 |
| CMOS + Rop$^+$ | 0.40 | 0.34 | 0.27 | 0.35 |
| CISA + Rop$^+$ | 0.99 | 0.92 | 0.89 | 0.89 |
| CISA + PCDP | 1.95 | 1.78 | 1.59 | 0.99 |

PCDP gives the smallest hue-angle deviation at every optical thickness, with a four-level mean of $28.15°$. Its advantage is largest at Level 4. The four-level mean deviations are $4.21°$ for `go`, $20.91°$ for `turn`, $39.58°$ for `40`, and $47.92°$ for `stop`. The `stop` region reaches $60.66°$ and $63.74°$ at Levels 3 and 4, respectively.

The chroma results qualify this improvement. PCDP is oversaturated at Levels 1–3 and approaches the reference chroma at Level 4. Its present advantage is therefore the preservation of hue direction, or chromatic identity, rather than exact recovery of color magnitude.

### S.9.4 Downstream person detection

We evaluated the four image types using a COCO-pretrained YOLO26m detector without fine-tuning. To remove the resolution advantage of the PCDP output, all images were bicubically resampled to $123 \times 82$ pixels before inference. The detector used a fixed input size and a confidence threshold of 0.01. Three fixed target positions were evaluated at each of four levels, and a missed target was assigned zero confidence.

Table S.13 Person detection after all images were resampled to a common resolution. Mean confidence includes zeros for missed detections.

| Method | Detected targets | Mean confidence |
|---|---|---|
| Scattered image | 0/12 | 0.000 |
| CMOS + Rop$^+$ | 2/12 | 0.021 |
| CISA + Rop$^+$ | 8/12 | 0.280 |
| CISA + PCDP | **12/12** | **0.698** |

Only CISA + PCDP detects all 12 targets. Repeating the evaluation at $184 \times 123$ pixels and at native resolution leaves the detection counts unchanged, while the mean confidence changes by less than 0.006. This result is a comparative test on one scene rather than a general detection benchmark, and the reported confidence values are not calibrated probabilities.

## S.10 Additional ablation study

### S.10.1 The impact of lens vignetting and dust

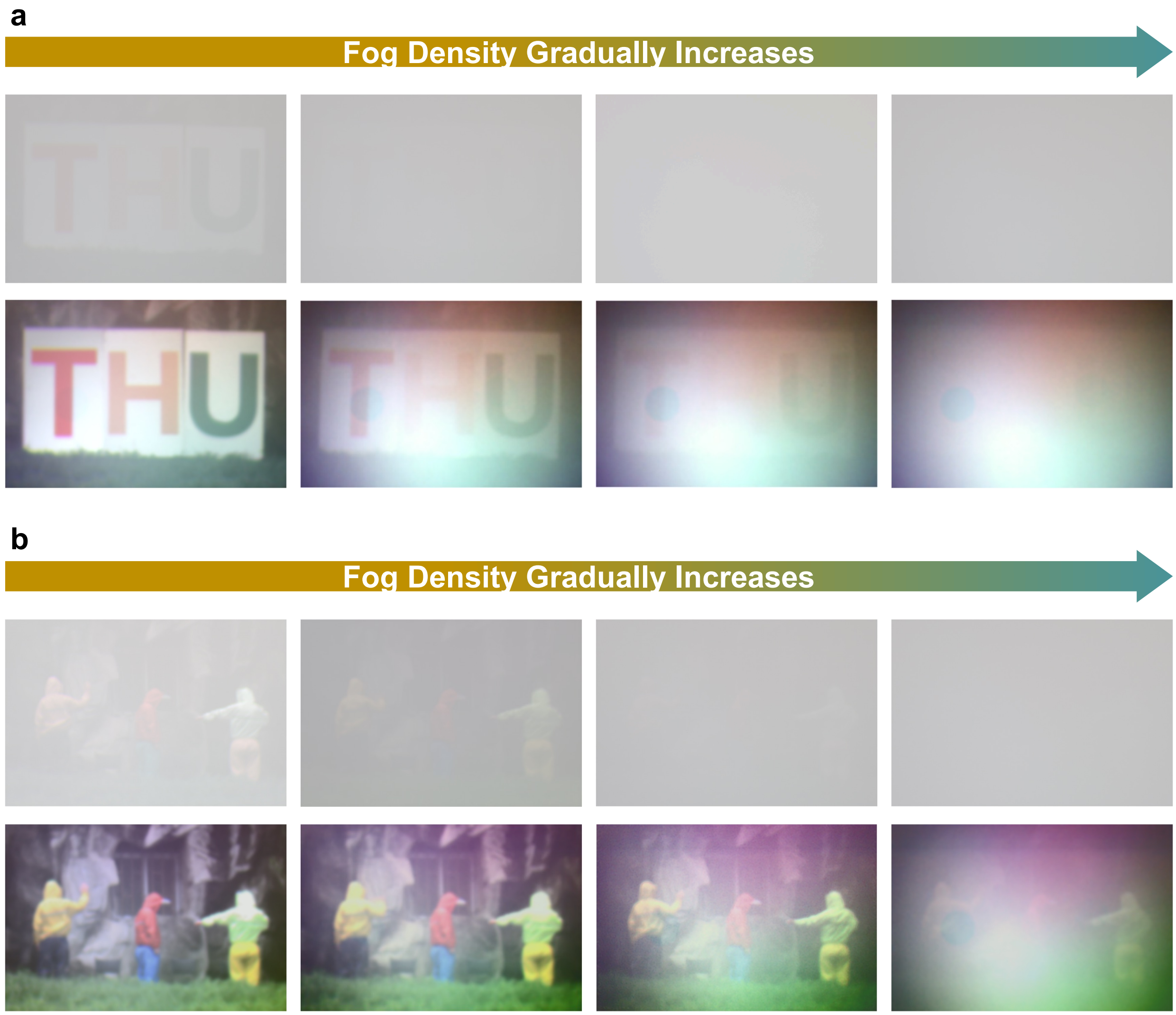


Figure S.22 **Effect of de-scattering enhancement under different scattering levels.** All results are obtained from the PCDP method without using the light-field comprehensive correction module.

Without using the proposed light field comprehensive compensation module, it can be observed from Fig. S.22**a** and **b** that for planar and 3D targets, the de-scattering results show that in cases of weak scattering, lens vignetting and dust do not significantly affect the de-scattering effect. However, as the scattering level increases, lens vignetting gradually disturbs the image, and circular spots caused by lens dust become more apparent. Single de-scattering algorithms alone cannot

solve these issues.

## S.10.2 The impact of illumination direction

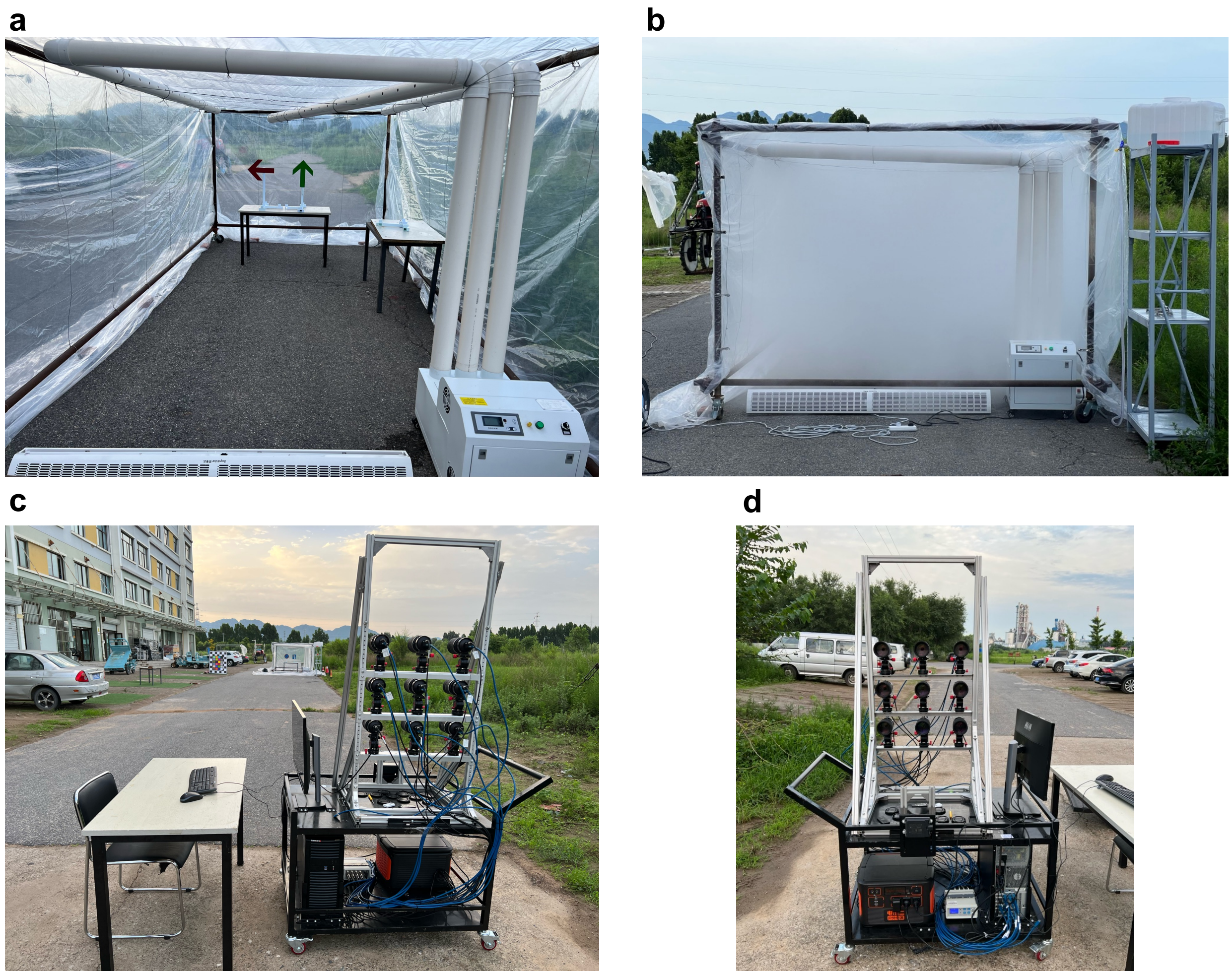

Figure S.23 **Natural illumination and artificial fog system. a** Outdoor fog generation system. **b** Artificial fog in a real scene. **c** Rear view of the scattering data collection end **d** Front view of the scattering data collection end.

To verify the effect of sunlight direction on de-scattering, we set up an outdoor fog generation system under natural illumination to conduct de-scattering tests at different times of the day. Specifically, we used a mist machine to construct a semi-enclosed fog box, as shown in Figure S.13, with dimensions of 6 m × 3 m × 2 m. By covering the fog box with plastic film around its perimeter, we ensured that natural sunlight could illuminate the target normally while confining the dispersion of the mist. The target object to be observed was placed inside the semi-enclosed fog box, with

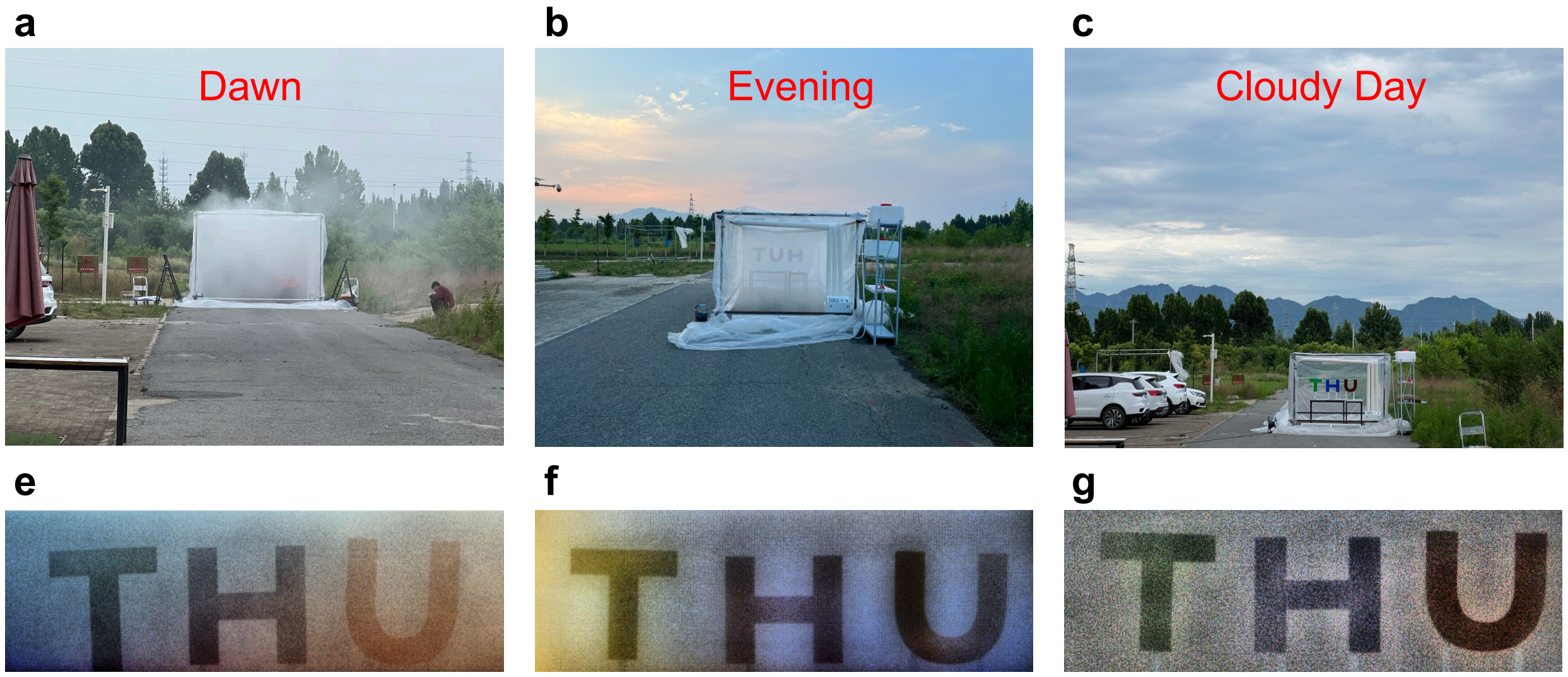


Figure S.24 **De-scattering effect under different sunlight angles at various times of the day.** **a-b-c** correspond to de-scattering tests conducted in the early morning (5 AM to 7 AM), evening (6 PM to 8 PM), and during overcast or rainy weather. **d-e-f** represent the de-scattering results under these weather conditions.

no film obstructing the space between the target and the image acquisition module of the system. The air curtain machine placed at the edge of the fog box served two purposes: (1) improving the uniformity of the mist inside the fog box through air circulation, and (2) forming an air curtain wall at the edge of the fog box, which not only isolated the internal and external air to enhance the stability of the dispersed mist but also prevented reflection interference on the camera caused by fully covering the fog box with film. The image acquisition module of the system consisted of nine FLIR BFS-U3-123S6C cameras (pixel size of $3.45 \times 10^{-6}$ m, maximum resolution of 4112×3008, maximum bit depth of 12 bits, and maximum frame rate of 30 Hz) paired with 135 mm fixed-focus lenses, a Continental ARS408 millimeter-wave radar, a square wave signal generator, a portable power supply, and a host computer, with a target observation distance of 50 m.

From Fig. S.24, it can be observed that due to differences in the sunlight's angle of incidence on the ground at different times of the day, the morning sunlight comes from the east, while in the evening, it comes from the west. On overcast or rainy days, due to significant scattering by the clouds, the direction of illumination can be considered relatively uniform. As a result, it can be seen that after de-scattering, the background light gradient in Fig. S.24 **a** and **b** is reversed, which corresponds to the opposite sunlight angles in the morning and evening. In contrast, the background in Fig. S.24 **c** appears more uniform. Therefore, in strong scattering scenes, the angle

of illumination from the light source cannot be ignored.

### S.10.3 Key Parameter Evaluation of the PCDP Algorithm

These ablation experiments in S.25 and S.14 clarify the rationale behind parameter selection and optimization:

**Effect of the $w$ parameter in HSV enhancement.** We evaluated the impact of the $w$ parameter in the HSV enhancement module with five representative values ($w = 1.2, 1.6, 2.0, 2.4, 2.8$). The results are shown in the first column of Fig. S.25. Larger $w$ values lead to stronger de-scattering effects.

**Effect of the inversion operator parameter $L$.** To assess the influence of the inversion operator parameter $L$, we tested five representative values ($L = 0.8, 0.9, 1.0, 1.1, 1.2$) while keeping other parameters fixed. The results are shown in the second column of Fig. S.25. Smaller $L$ values result in more pronounced de-scattering.

**Effect of the Legendre polynomial order $N$.** The choice of $N$ represents a balance between model complexity and fitting accuracy. We tested orders $N = 2, 3, 4, 5, 6$, and the results are shown in the third column of Fig. S.25. While higher $N$ can compensate the global illumination better, it also increases computational cost, requiring a trade-off in selecting the optimal order.

Table S.14 Observed effect of key parameters in de-scattering.

| Parameter | Tested values | Default | Observed Effect | Fig. S.25 |
|---|---|---|---|---|
| $w$ | 1.2, 1.6, 2.0, 2.4, 2.8 | 2.4 | $\uparrow w \Rightarrow$ stronger de-scattering | Col. 1 |
| $L$ | 0.8, 0.9, 1.0, 1.1, 1.2 | 0.9 | $\downarrow L \Rightarrow$ stronger de-scattering | Col. 2 |
| $N$ | 2, 3, 4, 5, 6 | 5 | $\uparrow N$ improves illumination fitting but increases cost. | Col. 3 |

### S.10.4 Quantitative Indoor Performance Comparison Testing of Imaging Units

This section presents quantitative indoor performance testing of the imaging units and algorithms to validate imaging performance under the experimental conditions described in Section S.5. The upper-left corner of the figure S.26 shows a clear, haze-free reference image, while the right

half displays the imaging results along with their corresponding SSIM metrics: from left to right, the degree of scattering gradually increases. From top to bottom, row **(a)** shows the original scattered images; row **(b)** displays the de-scattering results obtained using the DCP algorithm on a CMOS camera; row **(c)** displays the de-scattering results using our PCDP algorithm on a CMOS camera; row **(d)** presents the de-scattering results of a single imaging unit in the CISA system using PCDP under a global uniform exposure mechanism; and row **(e)** shows the de-scattering results of a single CISA imaging unit using PCDP under an independent exposure mechanism, captured at full well capacity. As indicated by the SSIM metric results, our imaging units combined with PCDP consistently achieve the highest performance scores across all scattering conditions.

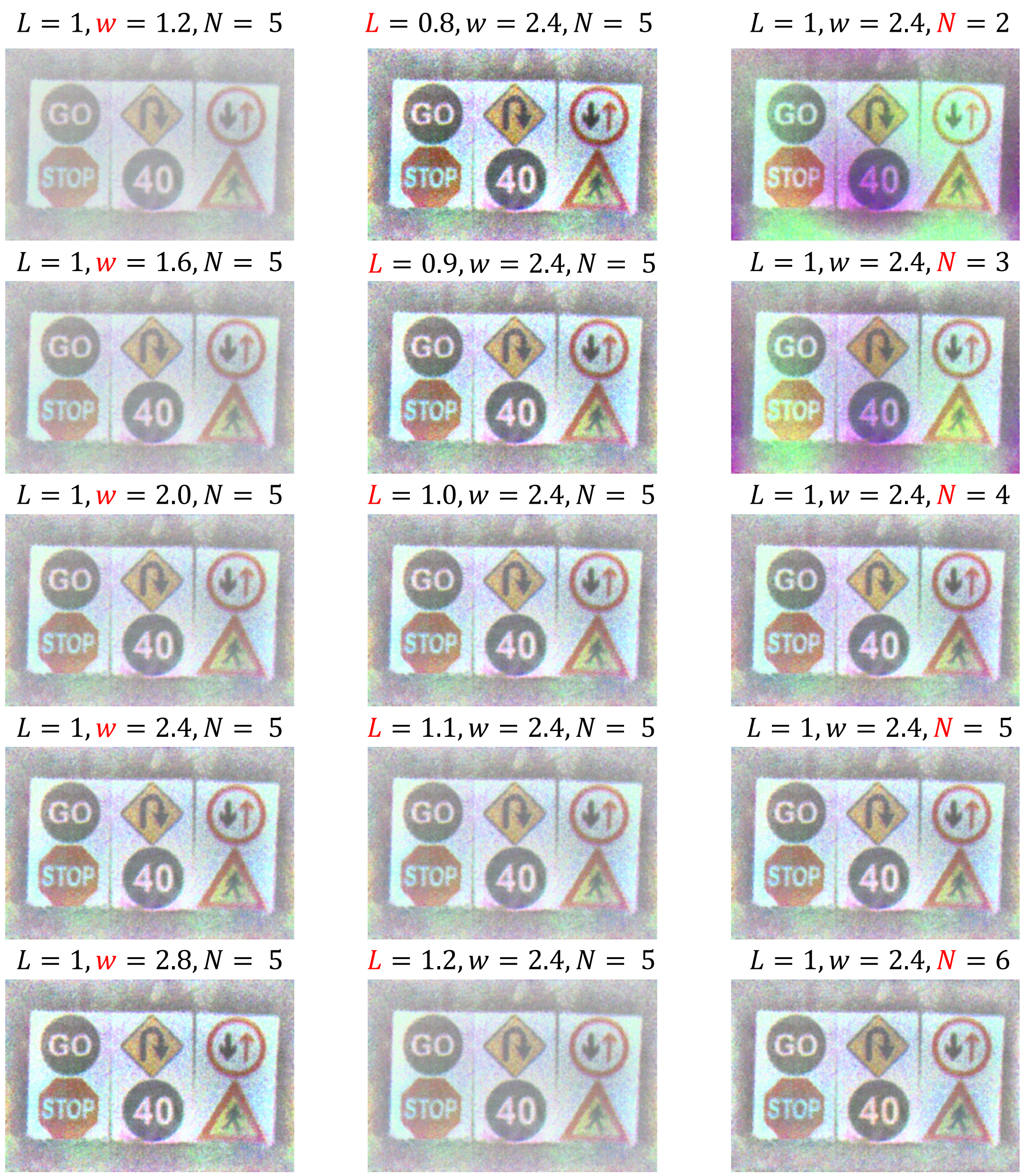


Figure S.25 **De-scattering performance comparison of different key parameters.**

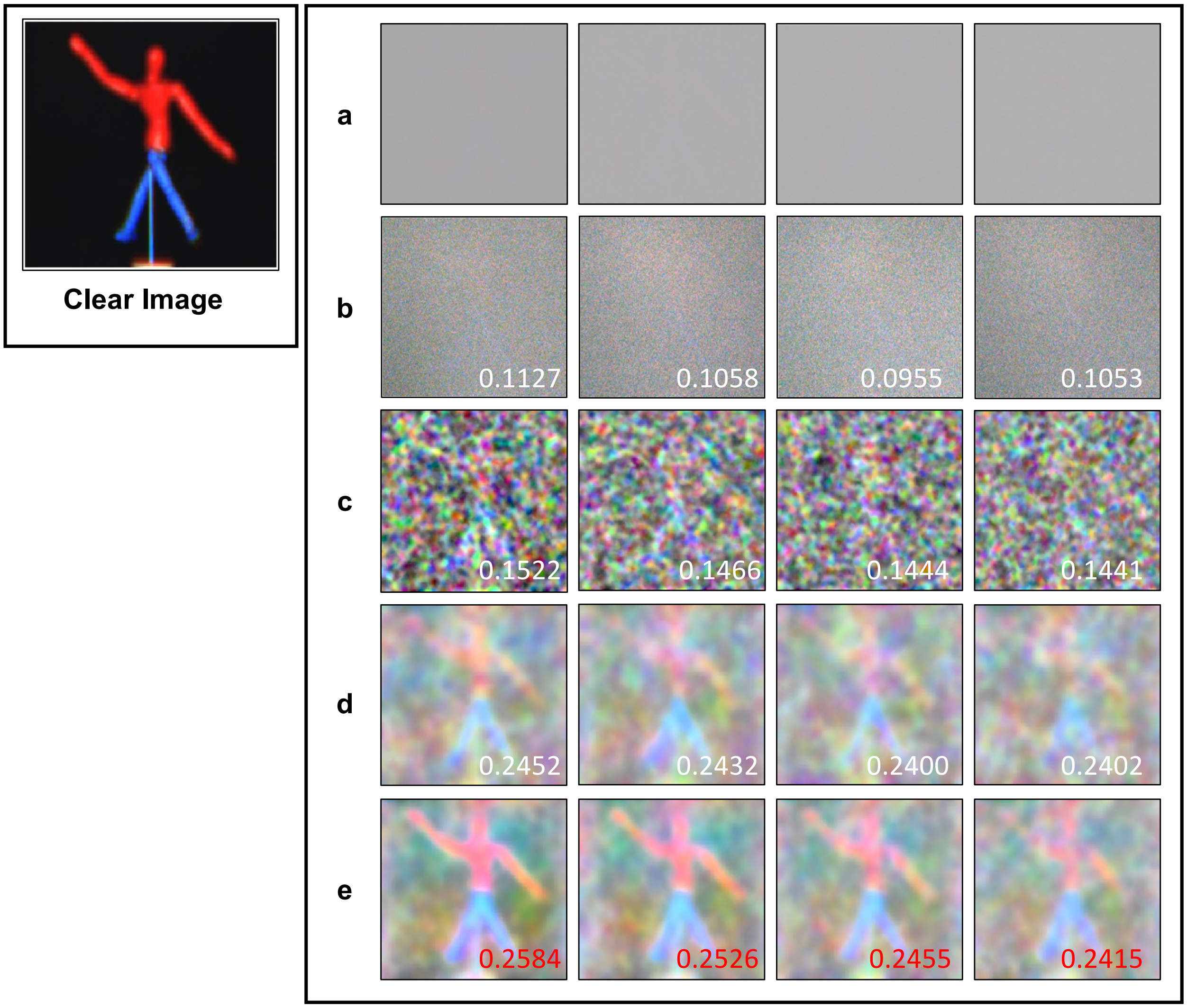


Figure S.26 **Quantitative Indoor Performance Comparison Testing of Imaging Units**

### S.10.5 Quantitative Analysis of Imaging Performance Factors

This section uses the experiments in Figs. S.19 and S.26 to quantify the effects of the reconstruction algorithm, detection platform, exposure strategy, array size, and number of fused frames. All images were resampled onto a common grid and evaluated using the same fixed regions of interest (ROIs). Levels 1–4 denote four increasing fog concentrations from left to right in Figs. S.19 and S.26.

**Stepwise ablation chain.** The five rows of Fig. S.26 form a stepwise ablation chain. Only one factor is changed at each step: the reconstruction algorithm (b→c, with the same hardware and global exposure), the detection platform (c→d, with the same algorithm and global exposure), and the exposure strategy (d→e, with the same hardware and algorithm). The gains at the four fog concentrations are summarized in Table S.15.

Table S.15 Stepwise contributions derived from the SSIM values in Fig. S.26.

| Step | Isolated variable | Level 1 | Level 2 | Level 3 | Level 4 | Mean |
|---|---|---|---|---|---|---|
| b→c | Algorithm (DCP → PCDP) | +35.1% | +38.6% | +51.2% | +36.9% | **+40.4%** |
| c→d | Platform (CMOS → CISA unit) | +61.1% | +65.9% | +66.2% | +66.7% | **+65.0%** |
| d→e | Exposure (global → channel-specific) | +5.4% | +3.9% | +2.3% | +0.5% | **+3.0%** |

The same order is observed at all four fog concentrations. The detection platform provides the largest contribution. The reconstruction algorithm provides a substantial additional improvement over DCP, while channel-specific exposure gives a smaller but consistently positive improvement.

The gain from channel-specific exposure decreases from 5.4% at Level 1 to 0.5% at Level 4. At lower fog concentrations, the spectral channels retain enough dynamic range for channel-specific exposure to reduce the original mismatch. Near the imaging limit, the target signals in all channels approach the noise floor, leaving less usable dynamic range to redistribute.

The platform and exposure steps were also checked using registered target ROIs. This analysis gave mean improvements of 38.8% for the platform and 3.3% for the exposure strategy, consistent with Table S.15. This check was limited to the comparisons in which the CISA images retained enough structure for reliable registration.

**Array size and frame count at a matched measurement budget.** Figure S.19 compares

the number of imaging units and fused frames. We define the effective number of measurements, $N$, as the product of these two quantities. Condition c (one unit and two frames) and condition d (two units and one frame) therefore have the same measurement budget, $N = 2$. Their contrast-to-noise ratios (CNRs) are listed in Table S.16. The uncertainty is the standard deviation among four non-overlapping spatial subregions within each ROI. It represents spatial nonuniformity rather than independent experimental repeats, so the paired comparisons are exploratory.

Table S.16 Contrast-to-noise ratio at the matched effective measurement budget $N = 2$.

| Level | CNR (c) | CNR (d) | d/c | Paired $p$ |
|---|---|---|---|---|
| 1 | $5.69 \pm 0.70$ | $5.33 \pm 0.18$ | 0.94 | 0.36 |
| 2 | $4.36 \pm 0.52$ | $4.10 \pm 0.42$ | 0.94 | 0.73 |
| 3 | $1.83 \pm 0.60$ | $1.94 \pm 0.98$ | 1.06 | 0.63 |
| 4 | $0.11 \pm 0.15$ | $0.30 \pm 0.23$ | – | Below threshold |

No clear difference is observed at the three fog concentrations for which the target remains detectable. At the same measurement budget, distributing measurements across two imaging units gives a result comparable to averaging two frames from one unit. Level 4 is excluded from the ratio analysis because both conditions are below the detection threshold.

At each detectable fog concentration, the CNR increases monotonically with $N$. Separate log–log fits for Levels 1–3 give exponents of 0.29, 0.34, and 0.39, respectively (Fig. S.27). Changing the Gaussian detrending width from 6 to 12 pixels changes these exponents by less than 0.03. The exponents are below the shot-noise-limited value of 0.5. The background residual standard deviation is at least 13 times the local quantization-noise estimate in every condition, showing that this difference is not caused by image quantization. Residual inter-unit registration errors and changes in the fog field during fusion both limit the averaging gain. Under the Rose criterion (CNR$\geq 3$), Levels 1 and 2 reach reliable detection at $N = 1$, Level 3 requires $N = 10$, and Level 4 remains below the threshold at $N = 20$.

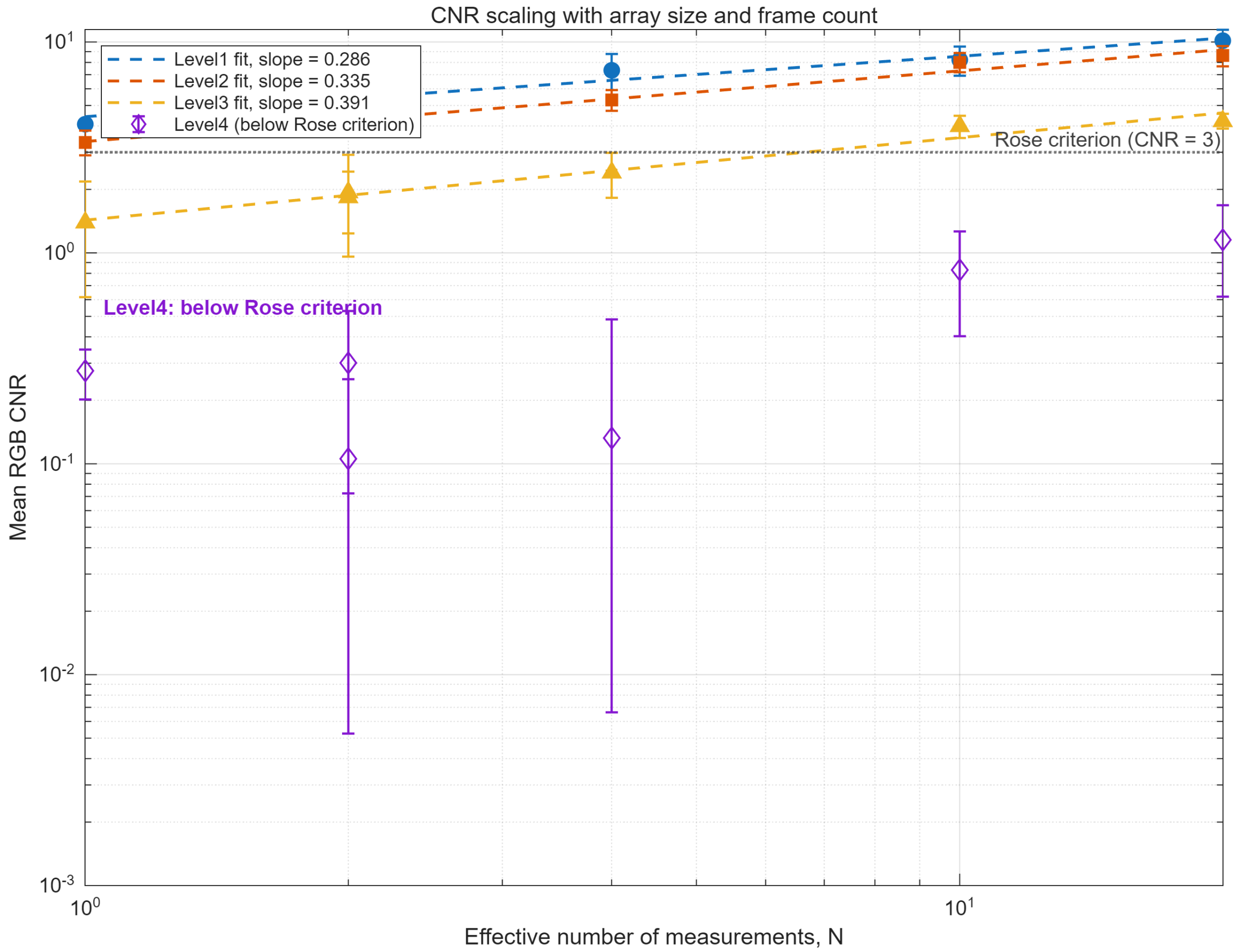


Figure S.27 **CNR as a function of the effective number of measurements.** Levels 1–3 are fitted separately on log–log axes. Level 4 is shown with open markers and is not fitted because all points remain below the Rose criterion. Error bars denote the standard deviation among four non-overlapping spatial subregions.

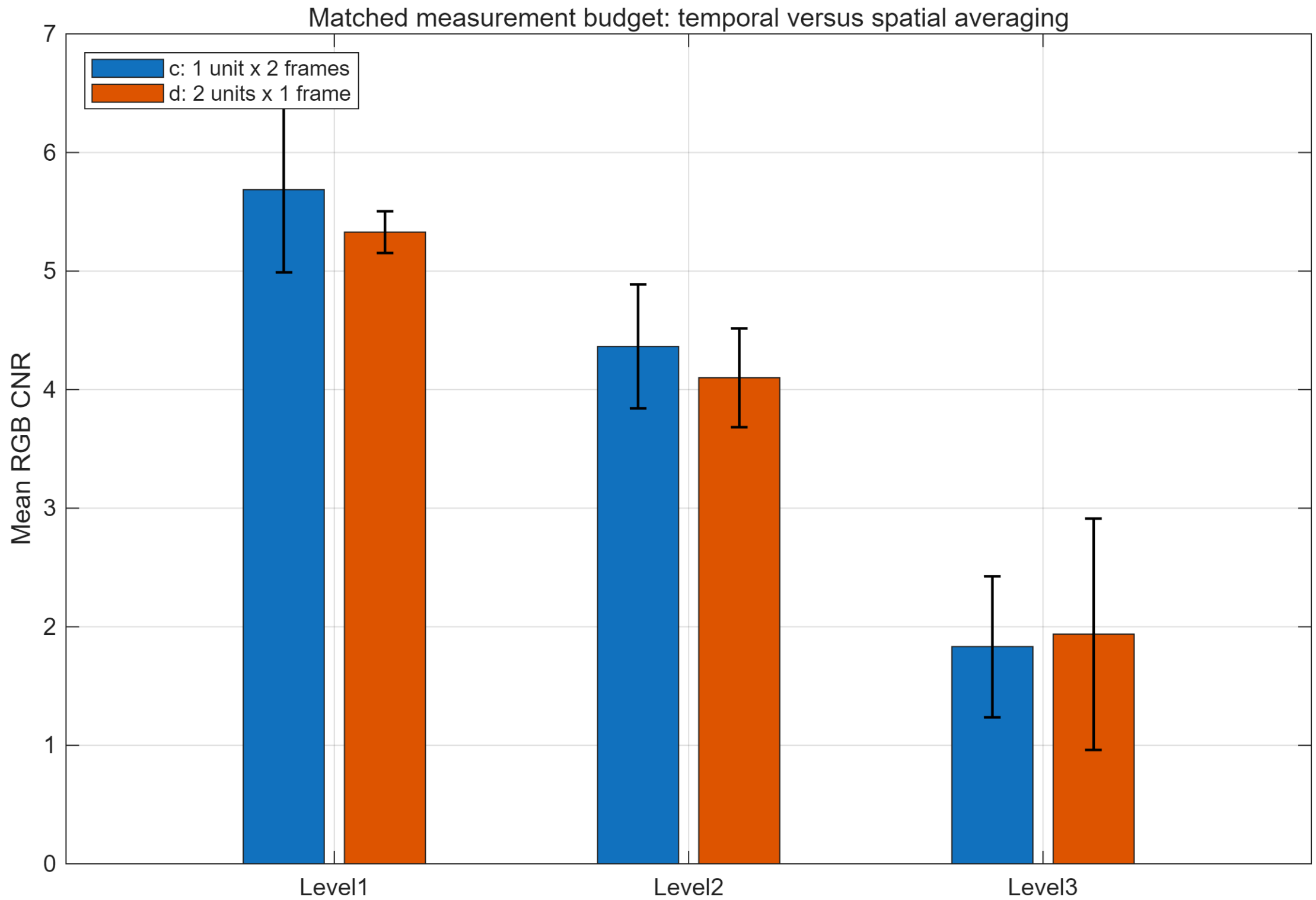


Figure S.28 **Matched-budget comparison of temporal and spatial averaging.** Condition c uses one imaging unit and two frames, while condition d uses two imaging units and one frame. Both conditions have $N = 2$. Level 4 is not shown because it is below the detection threshold. Error bars denote spatial-subregion standard deviations.

**Bit depth and full-well capacity.** Quantization is not a limiting factor for either detection system. For a full-well capacity of 10,575 electrons and 12-bit digitization, the quantization-noise standard deviation is 0.75 $\mathrm{e}^{-}$ rms. For a full-well capacity of 54,000 electrons and 16-bit digitization, it is 0.24 $\mathrm{e}^{-}$ rms. At the measured background level of approximately $10^{3}$–$10^{4}$ electrons, the corresponding shot noise is approximately 30–100 $\mathrm{e}^{-}$ rms. Quantization noise therefore contributes less than 0.1% of the total noise power in both systems and has a negligible effect on the observed performance difference.

**Temporal coherence of the scattering medium.** Sequential exposure bracketing requires the individual exposures to sample the same scattering pattern. We analyzed 10 consecutive images for each fog condition, acquired at 10 frames $\mathrm{s}^{-1}$, and measured the inter-frame correlation at the characteristic fog-cluster scale of 8–80 pixels over one frame period of 100 ms. After correction for the finite-sample null value, the correlations are 0.638 for the lower-fog sequence, 0.234 for medium fog, and 0.004 for dense fog. Under dense fog, the fog pattern is effectively decorrelated

within one frame period. Sequentially bracketed exposures therefore do not sample the same fog realization.

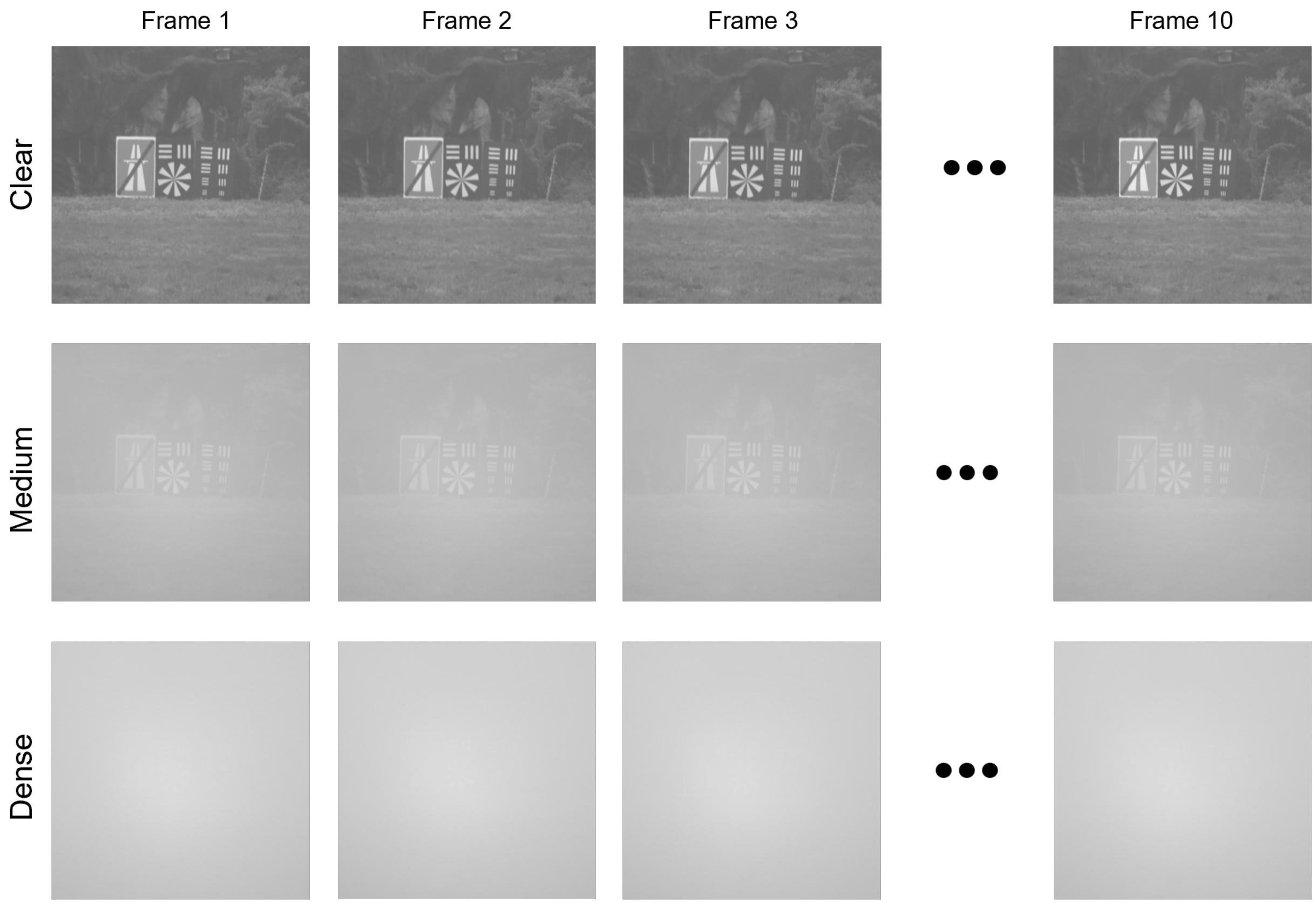


Figure S.29 **Representative images used for the temporal-correlation test.** The rows show the lower-, medium-, and dense-fog conditions from top to bottom. Selected frames from each 10-frame sequence are displayed. Consecutive images were acquired at 10 frames $s^{-1}$ with a 100-ms interval.

### S.10.6 De-scattering performance comparison of different algorithms

In Figs. S.30, S.31, S.32, and S.33, we compare the de-scattering results of various algorithms, including DCP (*15*), SLP (*32*), Rop$^+$ (*33*), Multifusion (*34*), and our PCDP algorithm. The results show that in strong scattering scenarios, issues such as lens vignetting, dust, and low SNR cause existing algorithms to struggle to recover target details. In contrast, our de-scattering framework demonstrates superior adaptability, effectively restoring both the contours and color information of the target.

### S.10.7 De-scattering performance comparison of different algorithms after preprocessing

To verify the effectiveness of the light field correction module proposed in our scheme, we first apply our correction module to the original image for preprocessing. Subsequently, we use DCP, SLP, $Rop^+$, Multifushion, and our PCDP algorithm to process the image respectively. Figure S.34, S.35 and S.36 illustrate that compared to scenarios without the correction module, all methods significantly improve the de-scattering quality, with target information no longer submerged in a chaotic background. This fully demonstrates the effectiveness of our light field correction module. On the other hand, the performance of these methods still falls short of our proposed strong scattering de-scattering framework, highlighting the significant advantages of the PCDP algorithm.

### S.10.8 Effectiveness of the self-guided filtering module

In strong scattering scenes, the use of V-guided filtering (VGF) operations has a significant effect on the recovery of target information. Based on S.1.5, existing prior-based de-scattering methods generally do not alter the H domain but instead enhance the saturation and value domains. However, based on the discussion of the color space in scattering scenes, the H domain has the lowest SNR, meaning that the hue information of the target is drowned in noise. Additionally, while the saturation domain has a moderate SNR, its noise is still significantly larger than that of the V domain. Therefore, it is necessary to perform denoising in the saturation domain while performing enhancement operations. To verify this theory, we conducted separate tests using the SLP method and our method in Fig S.37. The test procedure involved obtaining de-scattering results both without and with the VGF operation. It can be clearly observed that both the SLP method and our method exhibit similar phenomena, where the VGF operation significantly improves image quality. We then swapped the results in the HSV space. We found that if the de-scattered image used the non-denoised H domain, the overall image noticeably lost hue information. If the non-denoised S domain was used, the image noticeably lost saturation information. However, if the non-denoised V domain was used, the overall image quality degraded, but it did not significantly affect the human eye's ability to distinguish color information. Therefore, this matches our theory: denoising in the H domain and denoising and enhancing in the S domain are essential.

As shown in Fig. S.38, S.39 and S.40, using edge-preserving filtering significantly enhances the de-scattering effect of all methods, which is consistent with the theory in Sec. S.1.2.2. Since existing methods can only enhance the S and V domains but cannot address the high noise in the H domain, color information is submerged in noise. By leveraging the preprocessing of self-guided edge-preserving filtering, the aforementioned issues are effectively resolved, and the color information of the target is very effectively recovered, significantly improving the de-scattering performance of all methods.

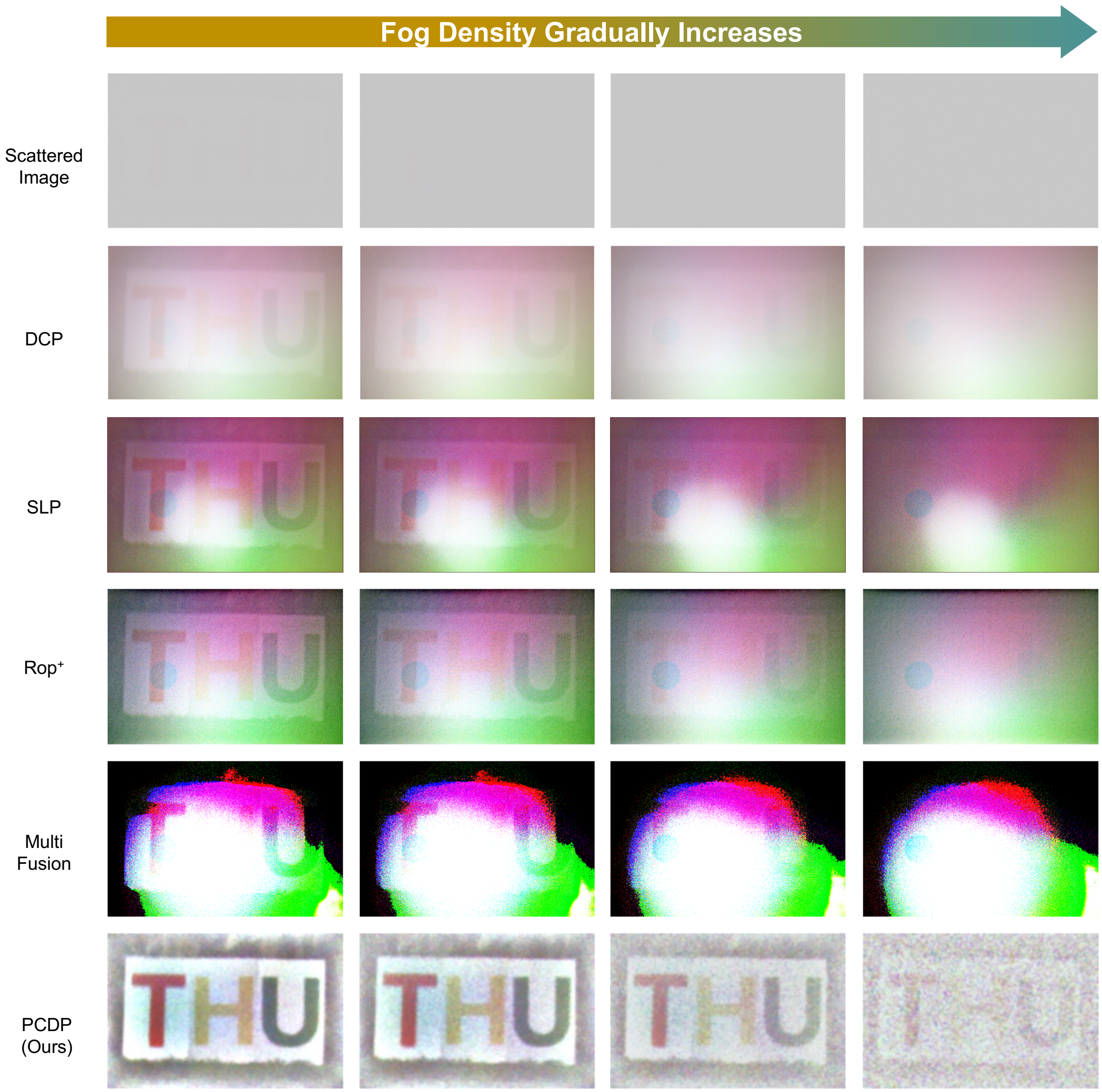


Figure S.30 **De-scattering results of the planar target "THU" letters.**

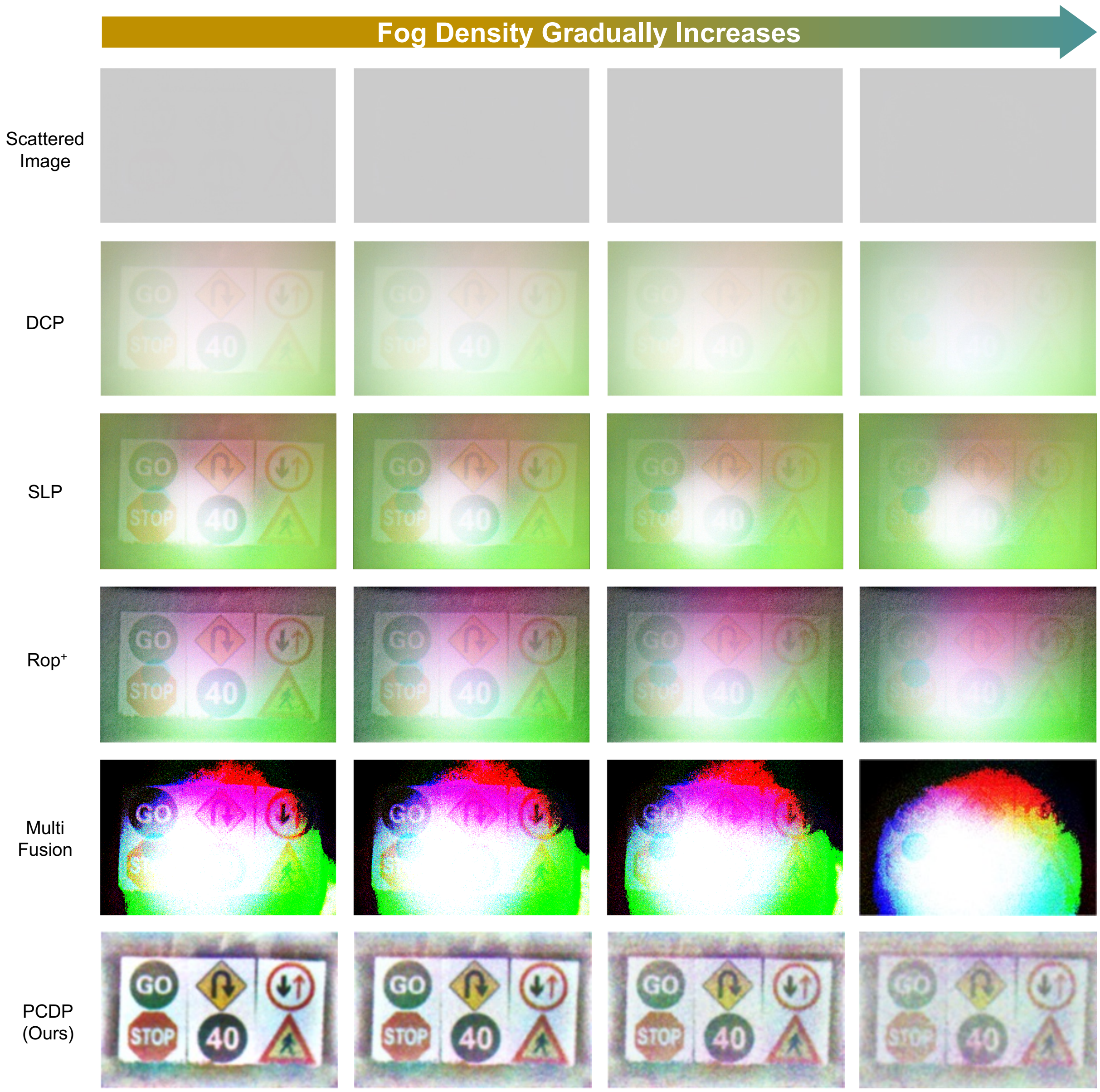


Figure S.31 **De-scattering results of the traffic signs.**

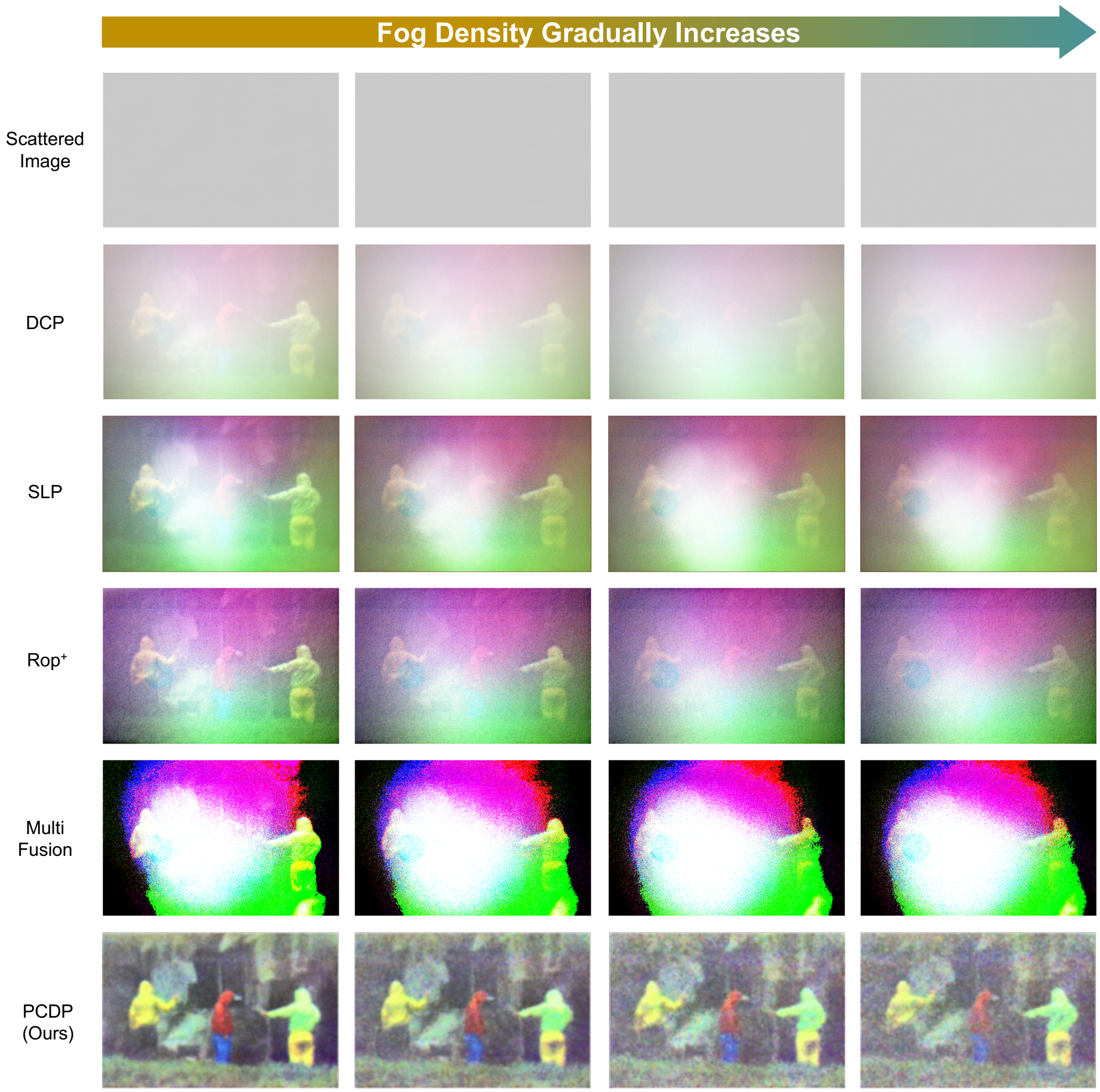


Figure S.32 **De-scattering results of the pedestrian postures.**

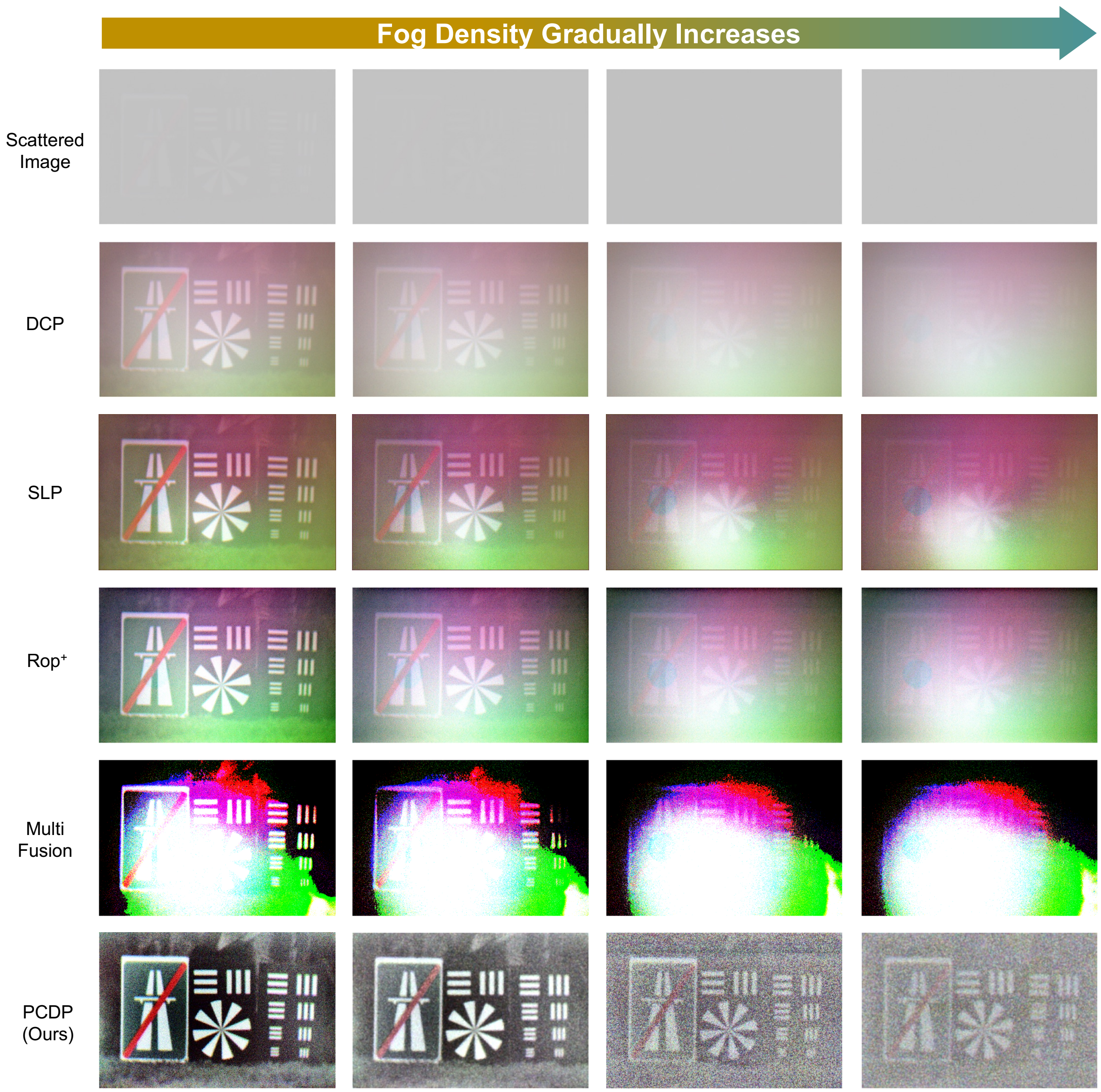


Figure S.33 **De-scattering results of the resolution chart.**

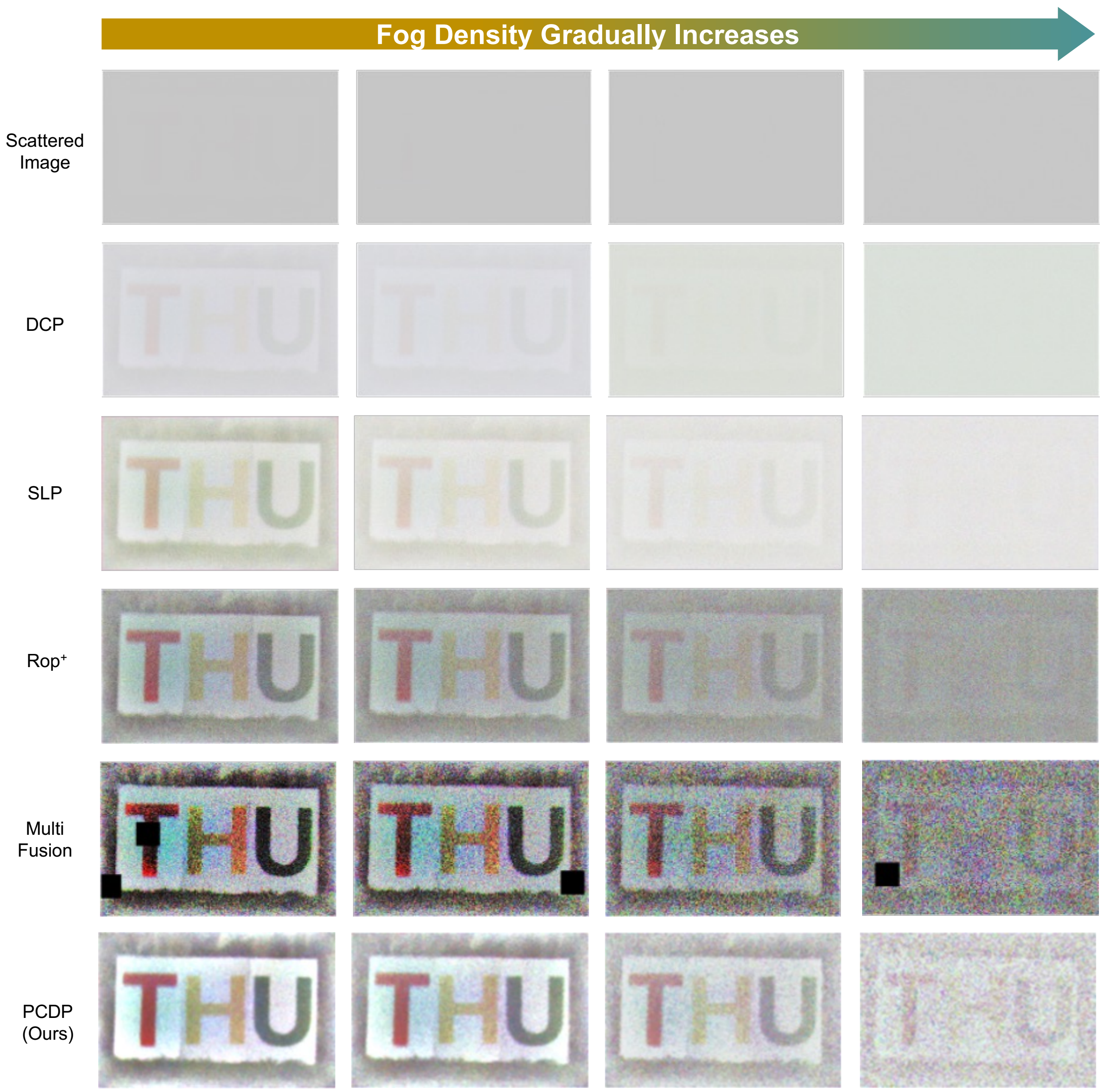


Figure S.34 **De-scattering results of the "THU" letters after using our light field comprehensive correction module.**

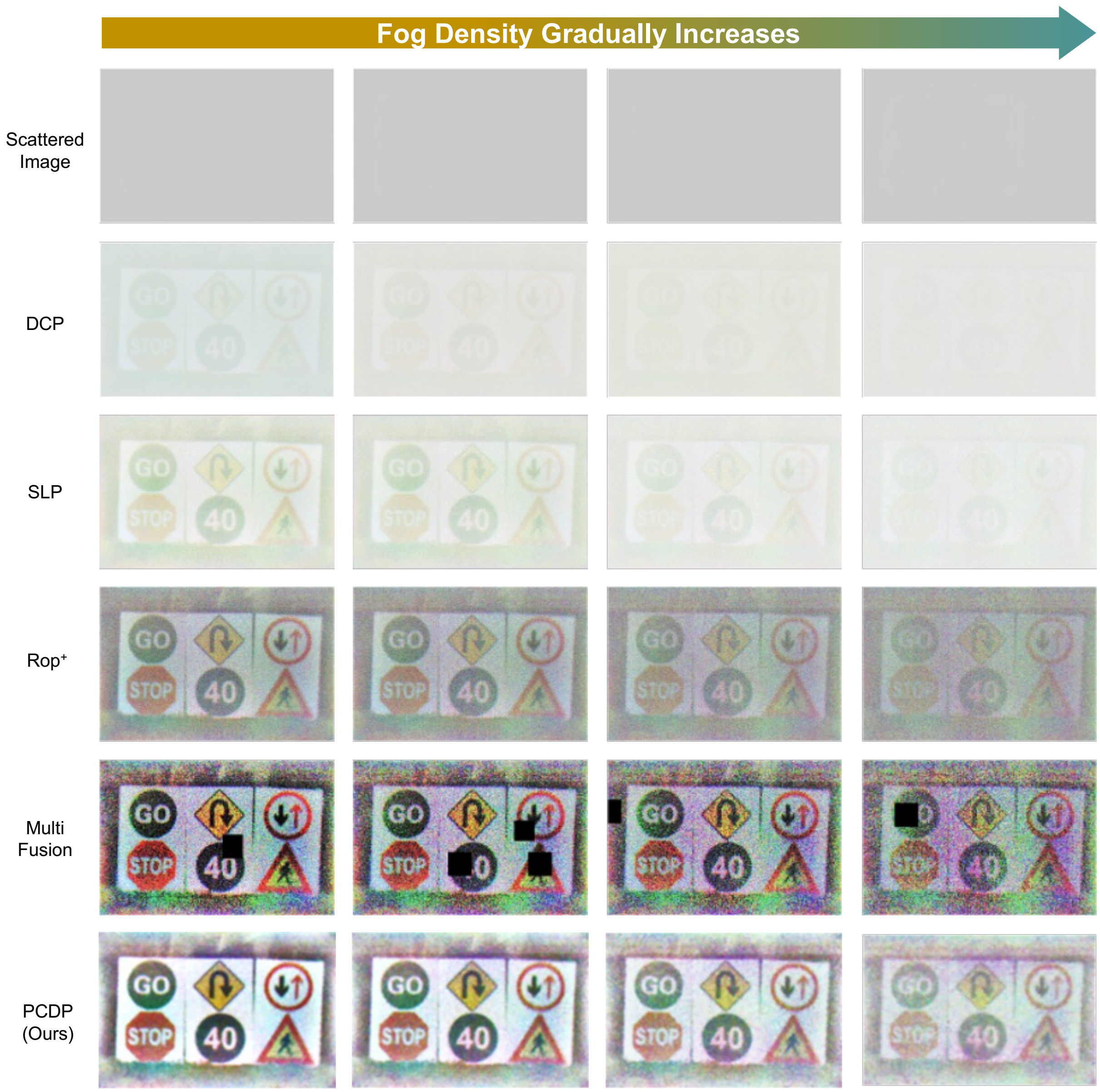


Figure S.35 **De-scattering results of traffic signs after using our light field comprehensive correction module.**

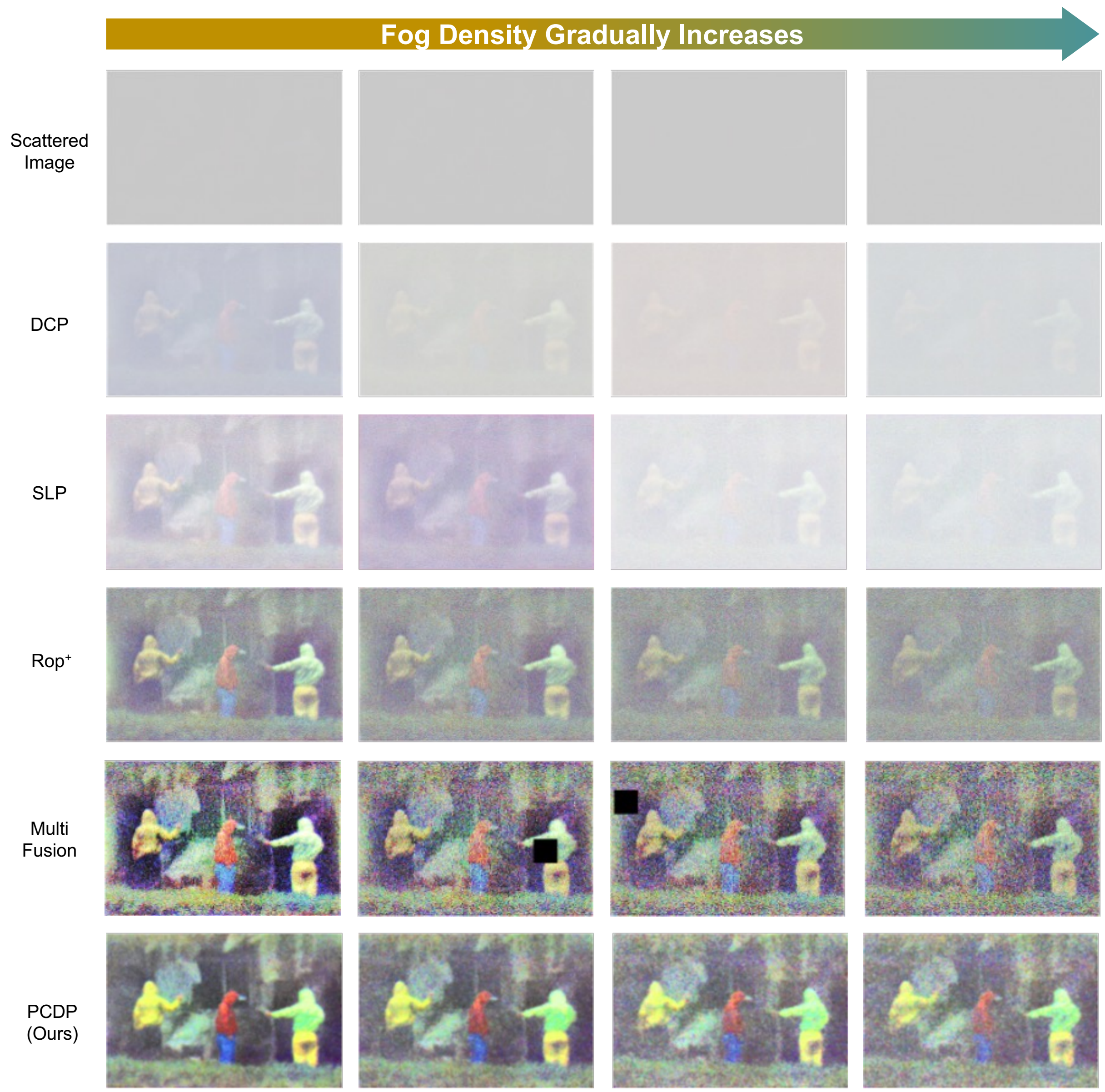


Figure S.36 **De-scattering results of pedestrian postures after using our light field comprehensive correction module.**

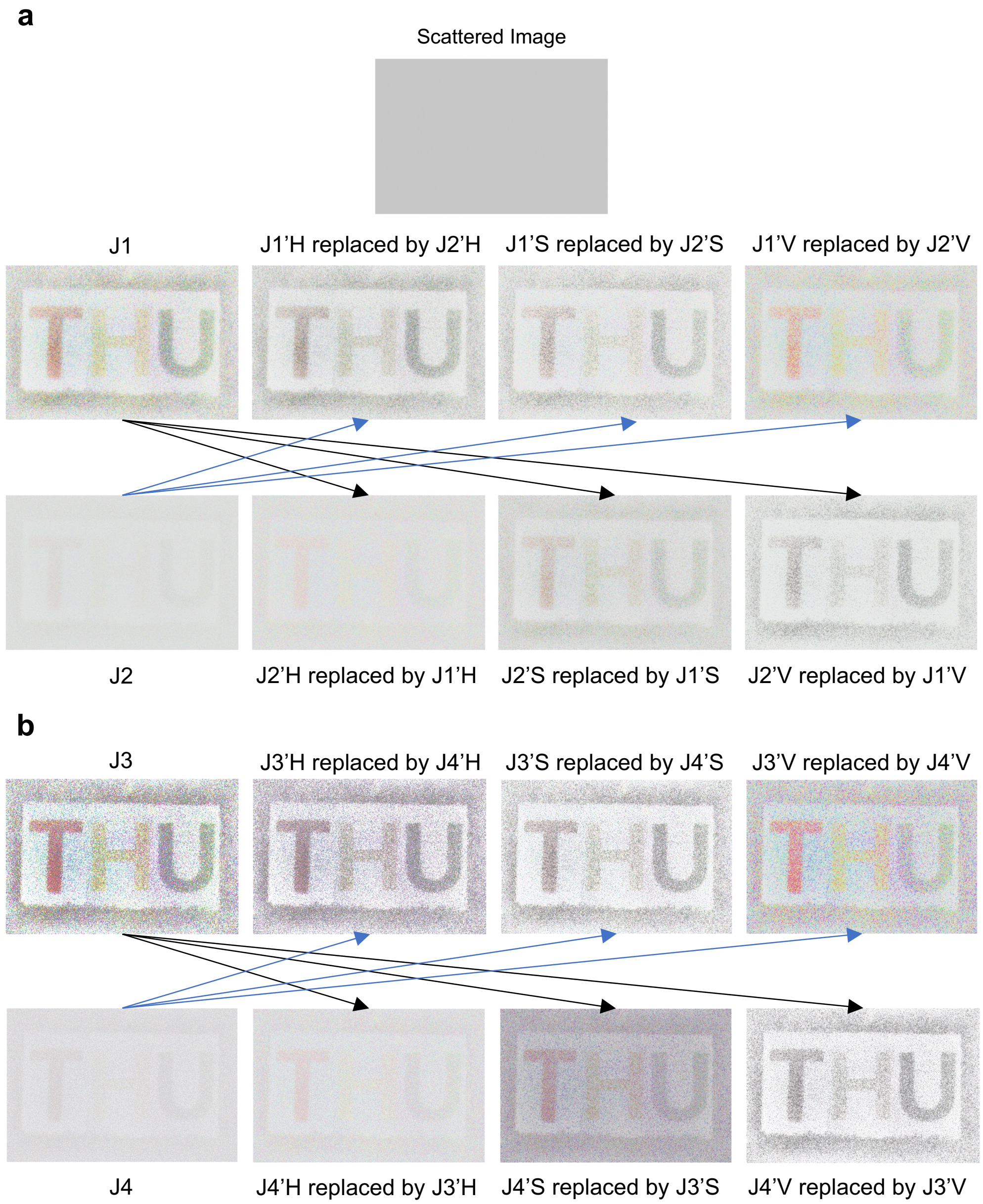


Figure S.37 **The regulation mechanism of the small template VGF module in the HSV space for de-scattering. a** Ablation experiment on the enhancement effect of VGF on the SLP method. J1:Results using the SLP method. J2:Results using the VGF+SLP method. **b** Ablation experiment on the enhancement effect of VGF on our method.J3:Results using our de-scattering enhancement module. J4:Results using VGF+our de-scattering enhancement module.

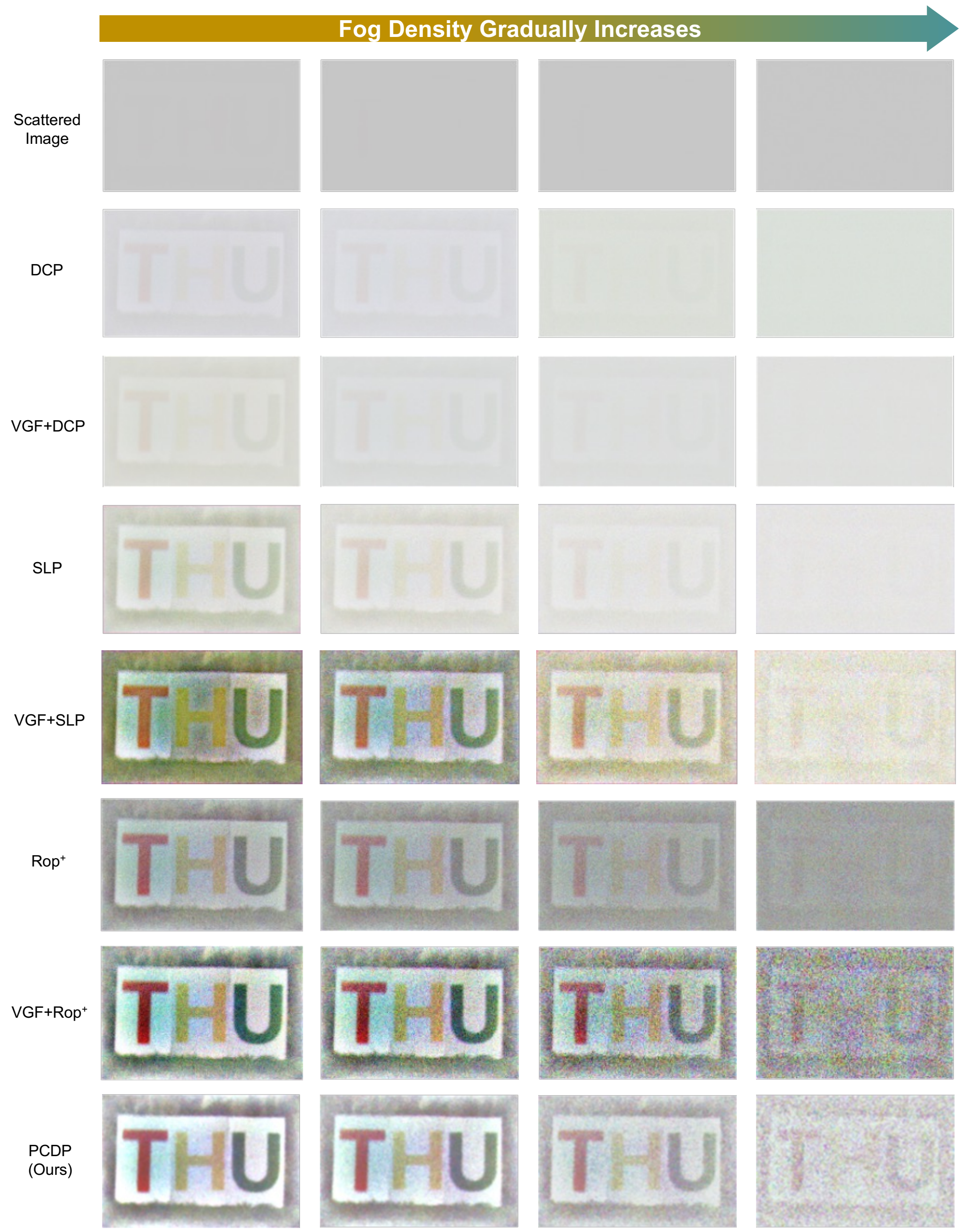


Figure S.38 **Performance improvement of with VGF operation for the "THU" letters observation.**

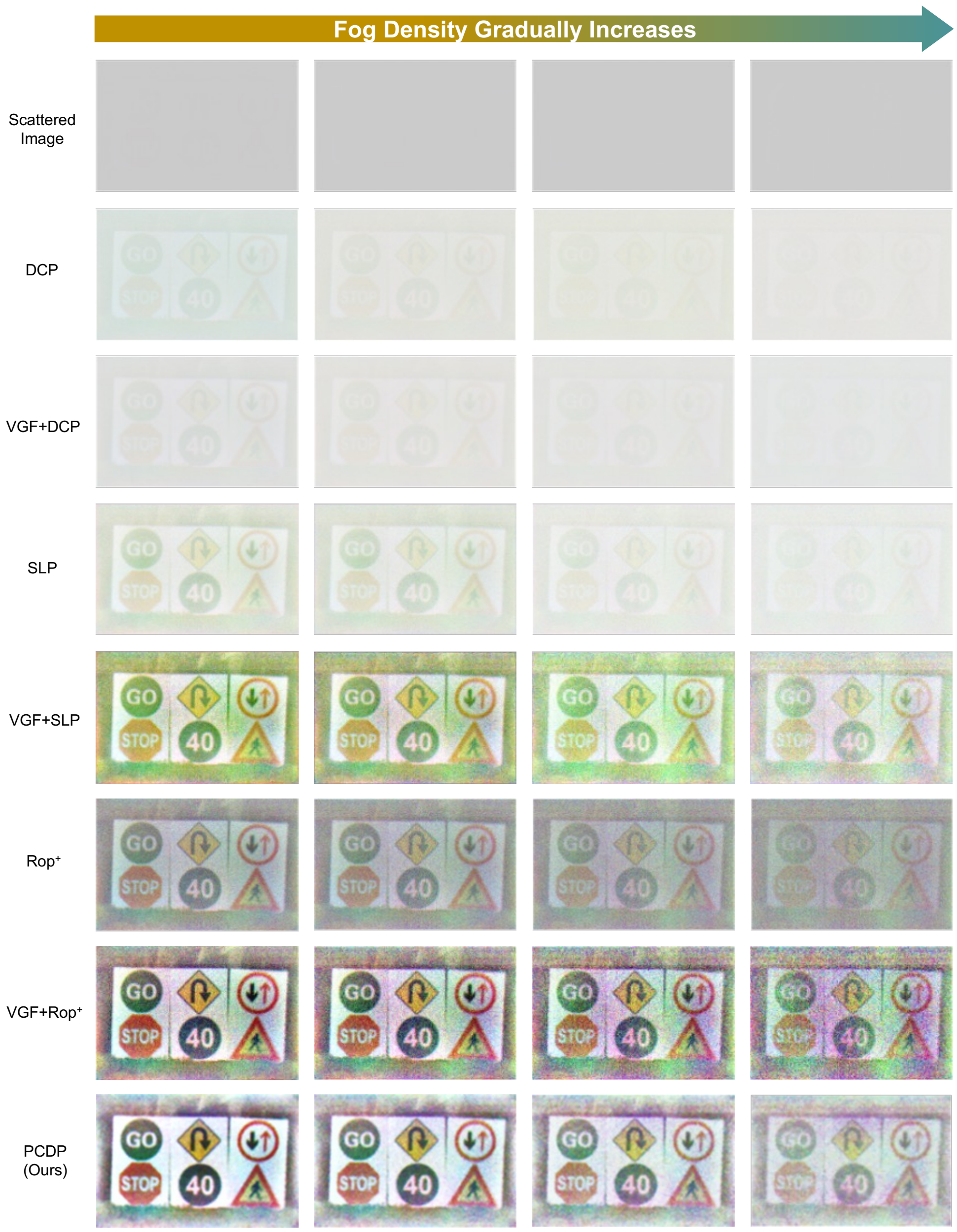


Figure S.39 **Performance improvement with VGF operation for the traffic signs.**

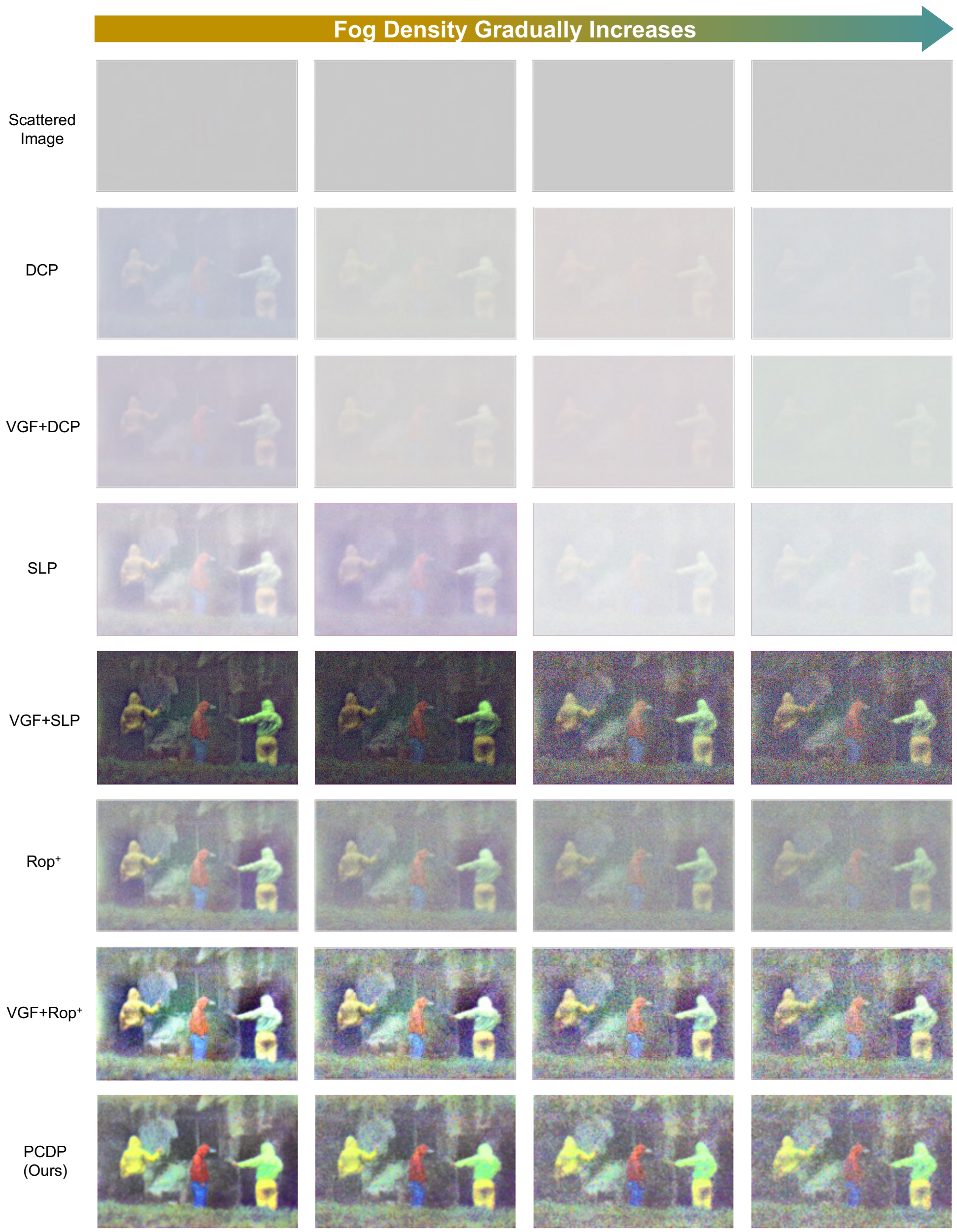


Figure S.40 **Performance improvement with VGF operation for the pedestrian postures.**

# References


1. N. Max, Optical models for direct volume rendering. *IEEE Transactions on Visualization and Computer Graphics* **1** (2), 99–108 (2002).

2. S. K. Nayar, S. G. Narasimhan, Vision in bad weather, in *Proceedings of the seventh IEEE international conference on computer vision* (IEEE), vol. 2 (1999), pp. 820–827.

3. M. Pharr, W. Jakob, G. Humphreys, *Physically based rendering: From theory to implementation* (MIT Press) (2023).

4. T. Chen, *Digital camera system simulator and applications* (stanford university) (2003).

5. B. E. Saleh, M. C. Teich, *Fundamentals of photonics, 2 volume set* (john Wiley & sons) (2019).

6. C. Beenakker, M. Patra, Photon shot noise. *Modern physics letters B* **13** (11), 337–347 (1999).

7. S. W. Hasinoff, Photon, poisson noise, in *Computer vision: a reference guide* (Springer), pp. 980–982 (2021).

8. J. R. Janesick, *Photon transfer* (SPIE press) (2007).

9. G. E. Healey, R. Kondepudy, Radiometric CCD camera calibration and noise estimation. *IEEE Transactions on Pattern Analysis and Machine Intelligence* **16** (3), 267–276 (1994).

10. S. W. Hasinoff, F. Durand, W. T. Freeman, Noise-optimal capture for high dynamic range photography, in *2010 IEEE Computer Society Conference on Computer Vision and Pattern Recognition* (IEEE) (2010), pp. 553–560.

11. W.-T. Kim, C. Park, H. Lee, I. Lee, B.-G. Lee, A high full well capacity CMOS image sensor for space applications. *Sensors* **19** (7), 1505 (2019).

12. A. Ajmal, C. Hollitt, M. Frean, H. Al-Sahaf, A comparison of RGB and HSV colour spaces for visual attention models, in *2018 International conference on image and vision computing New Zealand (IVCNZ)* (IEEE) (2018), pp. 1–6.

13. D. J. Lockwood, Rayleigh and Mie scattering. *Encyclopedia of color science and technology* pp. 1097–1107 (2016).

14. S. G. Narasimhan, S. K. Nayar, Vision and the atmosphere. *International journal of computer vision* **48** (3), 233–254 (2002).

15. K. He, J. Sun, X. Tang, Single image haze removal using dark channel prior. *IEEE transactions on pattern analysis and machine intelligence* **33** (12), 2341–2353 (2010).

16. D. Berman, S. Avidan, *et al.*, Non-local image dehazing, in *Proceedings of the IEEE conference on computer vision and pattern recognition* (2016), pp. 1674–1682.

17. A. Stuart, K. Ord, *Kendall's advanced theory of statistics, distribution theory*, vol. 1 (John Wiley & Sons) (2010).

18. R. C. Elandt-Johnson, N. L. Johnson, *Survival models and data analysis* (John Wiley & Sons) (2014).

19. Z. Zhang, A Flexible New Technique for Camera Calibration. *IEEE Transactions on Pattern Analysis and Machine Intelligence* **22** (11), 1330–1334 (2000), doi:10.1109/34.888718.

20. A. Galdran, A. Alvarez-Gila, A. Bria, J. Vazquez-Corral, M. Bertalmío, On the duality between retinex and image dehazing, in *Proceedings of the IEEE conference on computer vision and pattern recognition* (2018), pp. 8212–8221.

21. E. Boss, W. H. Slade, M. Behrenfeld, G. Dall'Olmo, Acceptance angle effects on the beam attenuation in the ocean. *Optics Express* **17** (3), 1535–1550 (2009).

22. L. Kou, D. Labrie, P. Chylek, Refractive indices of water and ice in the 0.65–2.5 $\mu$m spectral range. *Applied Optics* **32** (19), 3531–3540 (1993).

23. R. M. Pope, E. S. Fry, Absorption spectrum (380–700 nm) of pure water. II. Integrating cavity measurements. *Applied Optics* **36** (33), 8710–8723 (1997).

24. G. M. Hale, M. R. Querry, Optical constants of water in the 200-nm to 200-$\mu$ m wavelength region. *Applied optics* **12** (3), 555–563 (1973).

25. M. Babin, D. Stramski, Light absorption by aquatic particles in the near-infrared spectral region. *Limnology and Oceanography* **47** (3), 911–915 (2002).

26. J. W. Goodman, *Introduction to Fourier Optics* (W. H. Freeman, New York), 4 ed. (2017).

27. R. D. Fiete, *Modeling the Imaging Chain of Digital Cameras* (SPIE Press, Bellingham, Washington) (2010), doi:10.1117/3.868276.

28. D. L. Fried, Optical Resolution Through a Randomly Inhomogeneous Medium for Very Long and Very Short Exposures. *Journal of the Optical Society of America* **56** (10), 1372–1379 (1966), doi:10.1364/JOSA.56.001372.

29. F. Roddier, The Effects of Atmospheric Turbulence in Optical Astronomy. *Progress in Optics* **19**, 281–376 (1981), doi:10.1016/S0079-6638(08)70204-X.

30. I. Boutle, J. Price, I. Kudzotsa, H. Kokkola, S. Romakkaniemi, Aerosol–Fog Interaction and the Transition to Well-Mixed Radiation Fog. *Atmospheric Chemistry and Physics* **18**, 7827–7840 (2018), doi:10.5194/acp-18-7827-2018.

31. M. Pierzyna, S. Basu, R. Saathof, Intercomparison of Flux-, Gradient-, and Variance-Based Optical Turbulence ($C_n^2$) Parameterizations. *Applied Optics* **63** (16), E107–E119 (2024), doi: 10.1364/AO.522538.

32. P. Ling, H. Chen, X. Tan, Y. Jin, E. Chen, Single image dehazing using saturation line prior. *IEEE Transactions on Image Processing* **32**, 3238–3253 (2023).

33. J. Liu, R. W. Liu, J. Sun, T. Zeng, Rank-one prior: Real-time scene recovery. *IEEE Transactions on Pattern Analysis and Machine Intelligence* **45** (7), 8845–8860 (2022).

34. C. O. Ancuti, C. Ancuti, Single image dehazing by multi-scale fusion. *IEEE Transactions on Image Processing* **22** (8), 3271–3282 (2013).
